# Balancing competing effects of tissue growth and cytoskeletal regulation during *Drosophila* wing disc development

**DOI:** 10.1038/s41467-024-46698-7

**Published:** 2024-03-20

**Authors:** Nilay Kumar, Jennifer Rangel Ambriz, Kevin Tsai, Mayesha Sahir Mim, Marycruz Flores-Flores, Weitao Chen, Jeremiah J. Zartman, Mark Alber

**Affiliations:** 1 Department of Chemical and Biomolecular Engineering, University of Notre Dame, Notre Dame, IN USA; 2 Department of Mathematics, University of California, Riverside, CA USA; 3 Interdisciplinary Center for Quantitative Modeling in Biology, University of California, Riverside, CA, USA; 4 Department of Biological Sciences, University of Notre Dame, Notre Dame, IN USA

**Keywords:** Embryogenesis, Computational models, Cytoskeleton

## Abstract

How a developing organ robustly coordinates the cellular mechanics and growth to reach a final size and shape remains poorly understood. Through iterations between experiments and model simulations that include a mechanistic description of interkinetic nuclear migration, we show that the local curvature, height, and nuclear positioning of cells in the *Drosophila* wing imaginal disc are defined by the concurrent patterning of actomyosin contractility, cell-ECM adhesion, ECM stiffness, and interfacial membrane tension. We show that increasing cell proliferation via different growth-promoting pathways results in two distinct phenotypes. Triggering proliferation through insulin signaling increases basal curvature, but an increase in growth through Dpp signaling and Myc causes tissue flattening. These distinct phenotypic outcomes arise from differences in how each growth pathway regulates the cellular cytoskeleton, including contractility and cell-ECM adhesion. The coupled regulation of proliferation and cytoskeletal regulators is a general strategy to meet the multiple context-dependent criteria defining tissue morphogenesis.

## Abstract (web summary)

The authors integrate computational and quantitative approaches to elucidate how organ shape arises through the interplay between multiple growth pathways through regulation of both proliferation and the cytoskeleton.

## Introduction

The final shape of an organ is a result of the dynamic interplay between cell-level developmental processes^1–4^. A major challenge in reverse engineering biological systems arises from the inherent complexity of interactions governing protein regulatory networks and multicellular interactions that occur over multiple spatial and temporal scales^5–8^. In particular, regulating features such as local tissue curvature, cell height, and nuclear positioning is critical for controlling the morphogenesis of multicellular organisms^9–11^. Iteration of morphological changes such as epithelial folding and flattening results in the formation of an organ’s final 3-D structure and function^12–14^. Untangling the regulation of multiple subcellular features that compete with each other and control morphogenesis requires systems-level, multi-scale approaches^13^ that incorporate highly complex biologically calibrated computational model simulations coupled with quantitative experimental analysis^15,16^.

In this paper, we decouple the roles of cell proliferation and spatiotemporal cytoskeletal regulation in facilitating shape changes during organ development by using the *Drosophila* wing imaginal disc as a model system. The wing disc is a pseudostratified epithelial vesicle comprising multiple cell types, including squamous, columnar, and cuboidal cells^17^. In early developmental stages, the wing disc undergoes rapid proliferation, starting from around 30 cells and maturing into an organ with approximately 35000 cells^17^. Wing disc cells are enclosed by an extracellular matrix (ECM) along with several cytoskeletal regulators that vary in structural functions. For instance, interactions between Actin and phosphorylated non-muscle myosin II (pMyoII) generate contractile forces necessary for interkinetic nuclear migration during cell division^11^. Further, $\beta$-Integrin ($\beta$PS) adheres the ECM to the cell basal surface^18^. Collagen IV (Col-IV), a key component of the ECM, maintains tensile strength and adhesion of cells with the ECM^19^. The dynamic regulation of the cytoskeleton is spatially patterned across the tissue. In particular, the wing disc starts as a flat tissue that thickens and acquires a dome shape as it grows in size. Further, shape changes in individual cells can regulate morphogen signaling through feedback to pattern cytoskeletal regulators^20^.

Forces generated in individual cells resulting from single-cell processes, including proliferation and cytoskeletal regulation, combine across a multicellular system to give rise to its shape. For instance, fold formation partially occurs due to differential cell proliferation rates^21^. Also, Nematbakhsh and Levis et al. found that patterning of actomyosin contractility along the apical-basal axis of pouch cells is necessary for generating the dome shape, and the ECM is critical for maintaining the shape^22^. Recently, Harmansa et al. demonstrated that variation between growth rates of the cellular and ECM layers in the *Drosophila* wing imaginal disc contributes to the bending of the tissue^23^. However, biophysical mechanisms that couple cell shape, cell mechanical properties, and proliferation are yet to be established fully^24,25^. In the case of *Drosophila*, a fundamental question lies in determining the systems-level mechanism driving the formation and maintenance of wing disc shape during the larval stage and the connection between such shape acquisition with patterning of cell identities and cellular properties, including growth^12,26,27^. For example, Decapentaplegic (Dpp), a key morphogen that patterns the anterior-posterior (AP axis), activates and patterns the Rho family of small GTPases to generate a gradient in cell height through regulation of actomyosin contractility^9,28^. Proper shape acquisition is critical for the fitness of the organism. For instance, the final wing shape is a crucial determinant in flight performance^29^.

To test how spatial patterning of cell mechanical properties and cell proliferation impact the tissue shape, we developed and calibrated a multi-scale spatial computational modeling approach incorporating the spatial patterning of fundamental subcellular properties, including subcellular mechanics and cell division dynamics. As a basis for our model, we adopted the general Subcellular Element (SCE) modeling approach^22,30,31^, which was previously used to determine the role of basal contractility and spatially varying ECM stiffness in generating the bent shape of the wing disc^22^. In the model, we introduce a cell division module that captures the interkinetic nuclear migration and mitotic rounding process within a pseudostratified epithelium^10,11^. Combining experiments and computational model simulations, we demonstrate that key regulators of tissue curvature include the ratio of apical to basal contractility, ECM stiffness, and cell-ECM adhesion. Additionally, proliferation promotes increased tissue height and basal curvature. Surprisingly, analysis of multiple growth regulators reveals distinct subclasses that result in two very different phenotypic outcomes to growth stimulation: increased basal curvature or flattening of the tissue as growth increases. Consequently, the dual action of growth on proliferation and cytoskeleton generates the ability to regulate tissue size and shape as independent features.

## Results

### The role of proliferation and cytoskeletal regulation in a pseudostratified epithelium

**Fig. 1:**
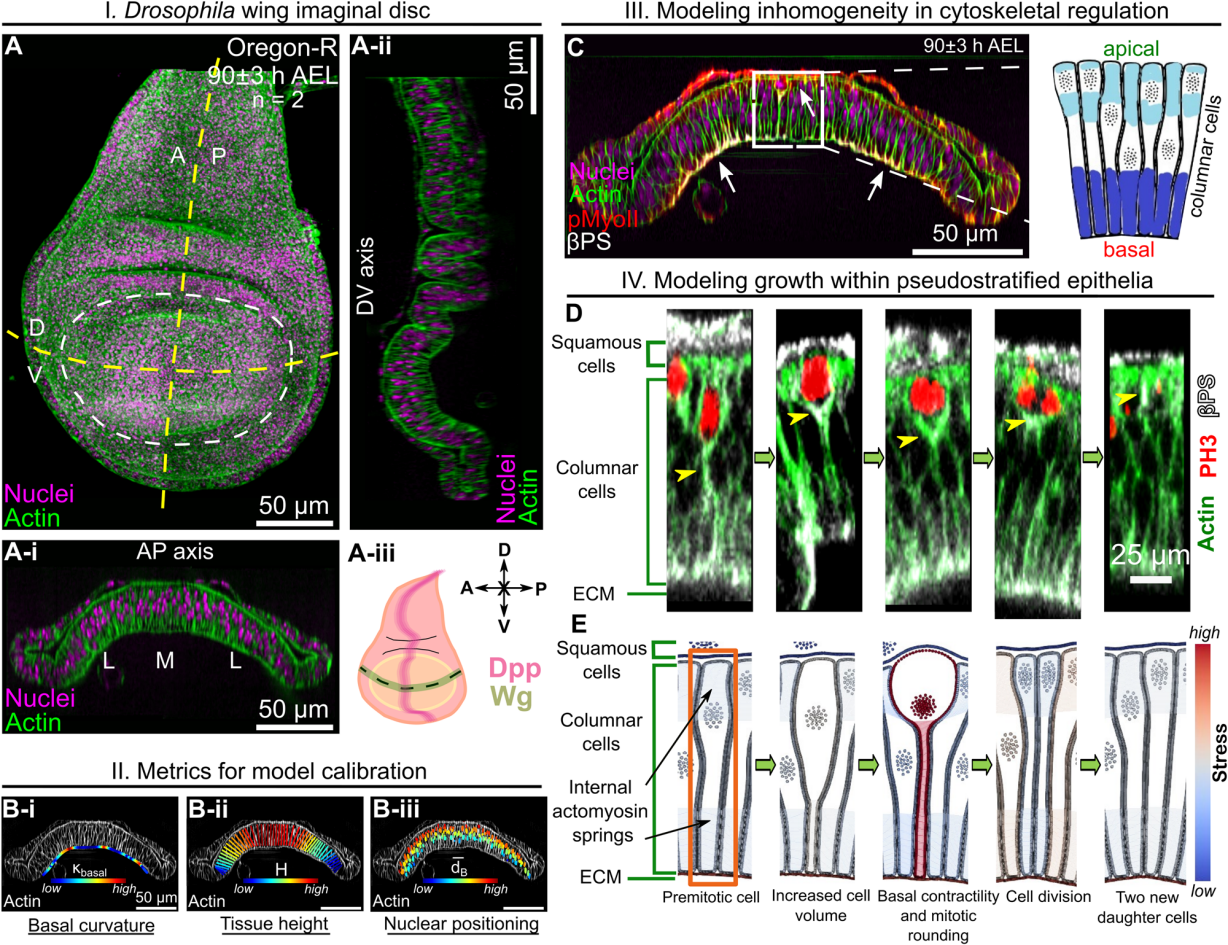
Multi-scale Subcellular Element (SCE) model simulation of proliferation in a pseudostratified epithelium predicts proliferation dynamics and cytoskeletal regulation. **A** Maximum intensity z plane projection showing the apical view of a wing imaginal disc at 90 h after egg laying (AEL). **A-i, A-ii** Cross-sectional views of the wing imaginal disc running parallel along the Anterior-Posterior (AP) and Dorsal-Ventral (DV) boundaries, respectively. The tissue is further divided into three equal parts, and the medial (M) and lateral (L) domains are defined in (**A-i**). **A-iii** A schematic illustrating patterning of the key morphogens, Decapentaplegic (Dpp) and Wingless (Wg). The inset on top-right specifies compartment orientation for data visualization and analysis. **B** Geometrical features defining the global tissue architecture include i. Local basal curvature (κ_basal_), ii. tissue thickness ($H$), and iii. relative nuclear distance from the basal surface ($\bar{{d}_{B}}=\frac{{d}_{B}}{{d}_{A}+{d}_{B}}$). The color code represents low to high numerical quantity. **C** Computational model incorporating spatial inhomogeneity of cell mechanical parameters. Parameters are determined based on experimental quantification. **D** Stages of interkinetic nuclear migration during cell division in wing disc cells. Yellow arrows show observed concentrations of Actin and $\beta$PS below the dividing cell. **E** Computational snapshots of a simulated columnar cell undergoing division. Black arrows point to the various apical and basal actomyosin contractile springs within the columnar cells.

**Fig. 2:**
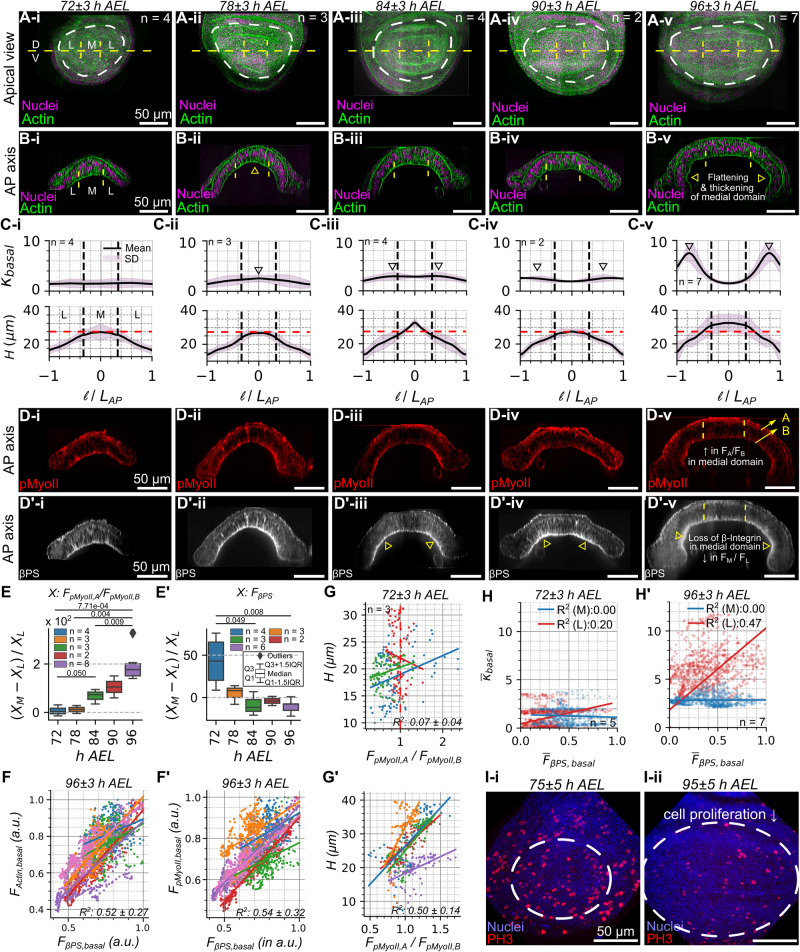
The central domain of the wing disc pouch flattens and thickens as it grows. **A i-v** Apical views of a wild-type wing imaginal disc at five different larval stages with medial (M) and lateral (L) domains defined. Fluorescence signals in green and magenta denote the patterning of Actin and Nuclei. **B i-v** Cross-sectional view of wing imaginal discs. **C** Plot showing quantification of (top panel) local basal curvature (${\kappa }_{{basal}}$) and (bottom panel) cell height (*H*) along the DV axis of the pouch. Here *l* and *L*_*AP*_ denote the distance of the point from center of the pouch along the basal surface of AP axis and the length of the tissue along the AP axis, respectively. **D**, **D’ i-v** Spatial patterning of pMyoII and $\beta$PS across the AP axis. **E** Quantification of the ratio of apical to basal intensities of pMyoII at multiple locations within the pouch’s medial domain is plotted as a box plot for discs belonging to 72-96 h AEL. **E’** Ratio of integrated intensity of $\beta$PS across the medial and lateral domains of the pouch. **F**, **F’** Fluorescence intensity of Actin, pMyoII, and $\beta$PS. Several points sampled in the basal surface for discs belonging to 96 h AEL larval stage. Average R^2^ values for multiple samples are indicated. Different colors represent data from individual samples. **G**, **G’** Plot showing a correlation between the ratio of apical to basal levels of pMyoII and tissue height belonging to 72 and 96 h AEL. R^2^ values calculated over multiple samples have been reported (right bottom inset). The different colors in each plot represent different samples. **H**, **H’** Fluorescence intensity of *β*-Integrin is plotted against the local basal curvature across several points sampled in the basal surface for discs belonging to 72 and 96 h AEL larval stage. Different colors represent the location of points within the pouch (medial/lateral). Region-specific R^2^ values calculated using multiple samples are indicated (sample sizes, right bottom inset). **I i-ii** Apical view of pouch showing that the fraction of dividing cells marked by phospho-histone 3 (PH3) decreases as terminal organ size approaches.

Here, we developed a computational model, combined with experiments, to test hypothesized mechanisms of shape changes in the wing imaginal disc during growth. In particular, we developed an SCE model that captures the shape of individual cells and overall wing disc morphology along the AP direction near the DV boundary (Fig. 1A-i). We also quantified important metrics defining the cross-sectional shape of the wing along the anterior-posterior axis (Fig. 1 Bi-iii). In our previous work^22^, it was assumed that actomyosin contractility is patterned uniformly across the apical and basal surfaces of the pouch. However, our experimental analysis (Figs. 1C and 2) of major cytoskeletal regulators reveals the nonuniform spatial patterning of mechanical regulators such as Actin, pMyoII, and Integrin (specifically $\beta$PS) across the AP axis. In particular, Actin, pMyoII, and $\beta$PS are more concentrated at the lateral basal ends than in the medial domain of the pouch at later stages of development (84 h AEL and later) (Fig. 1C). To test the significance of this asymmetry in cytoskeletal regulation in defining tissue morphology, we developed simulations that include spatial variation of cell-level mechanical properties. This spatial patterning of parameters across the AP axis for each cell is the first innovation of our previous work^22^.

Previous studies only allowed investigations of shape regulation at a single developmental stage and without spatial patterning of individual cell properties. As a second key innovation, we created the first SCE model that provides detailed simulations of cell proliferation and growth within a pseudostratified epithelium throughout the stages of organ development. The model of cell division includes multiple stages of interkinetic nuclear migration (Fig. 1D, E). During mitosis, the cell experiences narrowing of the basal section due to actomyosin contractility and depolymerization of the apical contractile springs. Consequently, the mitotic nucleus migrates towards the apical surface of the pouch. After division, the apical and basal contractile springs of the two new daughter cells are restored to pre-division values (Fig. 1E). Interestingly, both $\beta$PS and Collagen IV (Col-IV), key components of ECM-cell adhesion, form prominent tails below dividing cells (Fig. 1D, Supplementary Fig. 1). This may suggest that the basolateral surface of mitotic cells is pulled close to the bottom of the rounded cells during cell division to maintain a continuous structure for the epithelium to prevent delamination or excessive cell rearrangement.

Next, we developed a pipeline to calibrate the SCE model with the quantified experimental data for model calibration using local shape features such as basal curvature (${\kappa }_{{basal}}$), tissue thickness ($H$), and nuclear positioning ($\bar{{d}_{B}}$) (Fig. 1B-i, B-ii, B-iii). The computational model, along with the quantification of experimental data, enables us to reverse engineer mechanisms governing organ shape and size regulation.

### Both cell height thickening and tissue flattening occur towards the end of larval wing disc maturation

Experimental analysis of staged wing discs reveals cell height thickening and flattening of the medial basal surface of the pouch. In particular, shape changes are correlated with changes in cytoskeletal regulation. To understand the interrelationships between tissue growth and morphology, we quantified wing disc shapes over multiple time points leading up to pupal stages (72-96 h AEL Fig. 2A, B). Basal curvature (${\kappa }_{{basal}}$) along the DV boundary shows transitions from a relatively flat tissue (<72 h AEL) (Supplementary Fig. 2) to acquire a curved shape with almost uniform curvature (72-84 h AEL) (Fig. 2B, Ci-ii, top panel). The disc then continues to grow in size with increased bending in the lateral regions and flattening in the medial domain (84-96 h AEL) (Fig. 2B, Ciii-v, top panel). This central flattened section increases in width as development progresses (Fig. 2C, top panel). Moreover, cell height varies (Fig. 2C, bottom panel) across both the medial and lateral domains of the pouch.

**Fig. 3:**
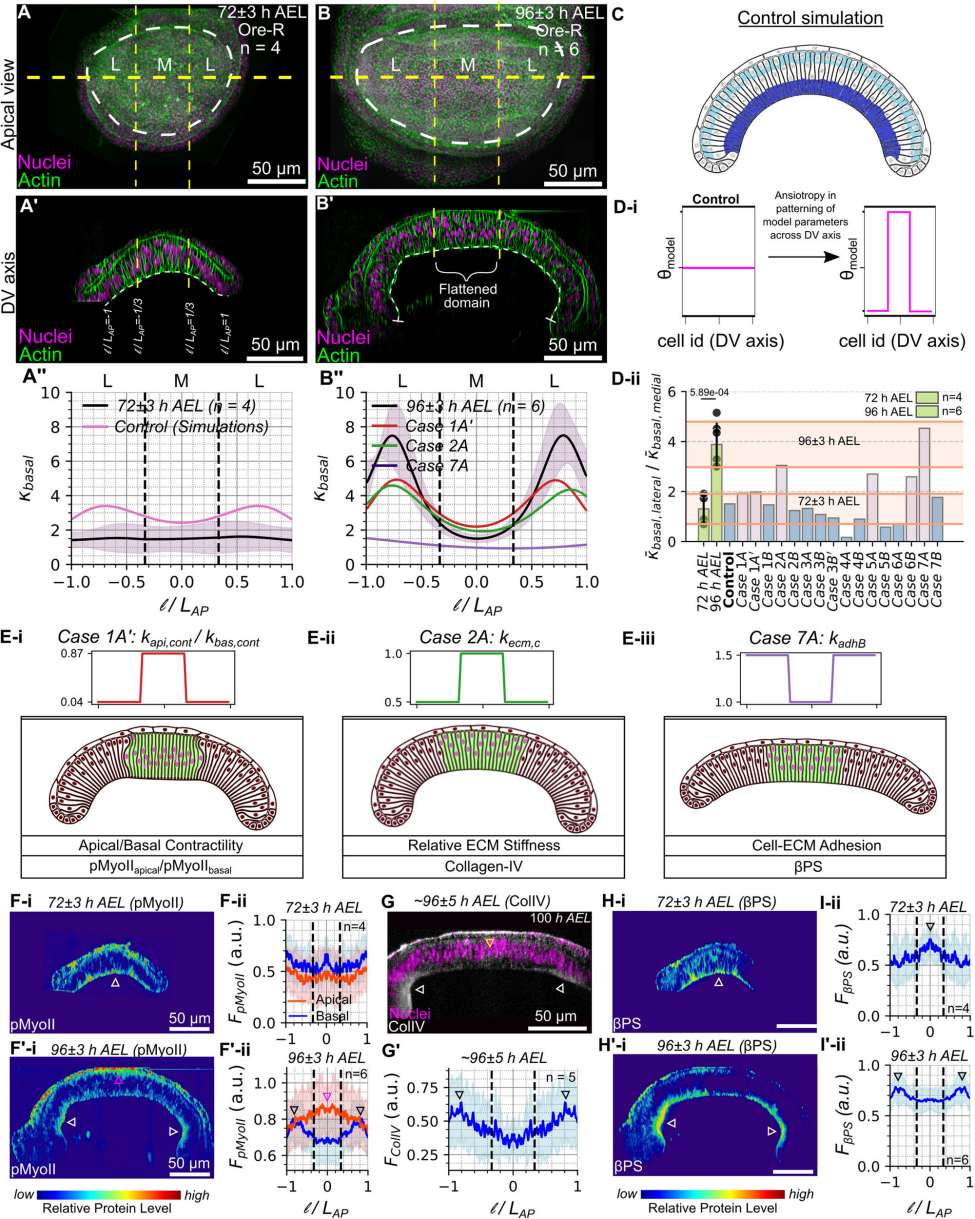
The evaluation of computational model scenarios validates cytoskeletal impact on tissue curvature via biomechanics. **A**, **B** Reference apical and (**A’-B’**) cross-sectional views of a wild-type (WT) disc stained with DAPI and Phalloidin at (**A**) 72 h and (**B**) 96 h AEL. **A”-B”) **Ratio of average curvature over the lateral and medial domain for staged discs (72 and 96 h AEL) compared with simulation results. Plots show the normalized curvature of the tissue basal surface (${\kappa }_{{basal}}$) calculated for samples corresponding to 72 h and 96 h, respectively. The red, green, and purple solid lines in (**B**”) correspond to the normalized basal curvature from simulation cases Ei-iii. **C** Control simulation output. **D**-**i** Perturbing model parameters (${\theta }_{{model}}$) that correspond to the cell cytoskeleton and cell-ECM adhesive energies. ${\theta }_{{model}}$ was patterned as a step function. **D**-**ii** Quantification of the ratio of average curvature in the lateral-to-medial pouch domains for staged discs (72 and 96 h AEL) and simulation results. Green bars denote the experimental data. Blue and pink bars represent the simulation data. The pink bars denote cases that capture medial flattening. Shaded regions represent the mean plus or minus one standard deviation. **E** **i**-**iii** Simulation results from varying model parameters representing mechanical properties of cell cytoskeleton. Parameter profiles are plotted. The green-colored region represents the medial domain of the simulated tissue. Case 1 A’ in **B”**, **D**-**ii** and **E**-**i** is Case 1D from Supplementary Fig. 7. See Supplementary Fig. 8. For details (**F**-**F**’ **i**) Heat maps of pMyoII distribution across the DV section for 72 and 96 h AEL discs. Color code represents lower (blue) to higher (red) intensity of pMyoII. **F**-**F**’ **ii**) Variation of pMyoII localization across the pouch apical and basal surfaces. Plots show intensity profiles across the AP axis. **G** Col-IV antibody staining in AP cross-section of 90-100 h AEL disc (**G’**) Spatial localization of Col-IV in the pouch basal surface. **H-H’ i** Heat maps of $\beta$PS distribution across the DV section of 72 and 96 h AEL discs. **I-I’ ii** Variation of $\beta$PS localization across the pouch basal surface. Plots show intensity profiles across the AP axis.

Our results also indicate a change in the spatial patterning of cytoskeletal regulators throughout development (Fig. 2D-F’). In particular, the relative levels of pMyoII shift apically in the pouch’s medial region as the discs grow (Fig. 2D i-v, E). Similar to pMyoII, $\beta$PS also undergoes temporal changes across the pouch basal surface (Fig. 2D’ i-v). During the initial stages of development, $\beta$PS is localized along the basal surface with increased localization in the medial regions. As the pouch grows in size, it becomes more dominantly localized across the lateral regions of the pouch (Fig. 2E’). The strong correlation of Actin, pMyoII, and $\beta$PS along the basal surface of pouch cells suggest potential functional associations between the different cytoskeletal components (Fig. 2F, F’, Supplementary Fig. 4a, b). Additionally, the ratio of apical to basal levels of pMyoII strongly correlates with tissue height at later stages of morphogenesis (Fig. 2G, G’). Further, $\beta$PS localization correlates with the basal tissue curvature. Overall, the regions within the basal surface of epithelia that have higher localization of $\beta$PS have higher basal curvature (Fig. 2H, H’). While local cell height exhibits a strong correlation with the ratio of apical to basal pMyoII for discs at later stages of development (Fig. 2G’), it cannot be conclusively asserted that this relationship is the exclusive mechanism of cell height regulation across the pouch AP axis. This point stems from the observation that columnar cell height decreases when transitioning away from the medial pouch domain in younger discs (Fig. 2C-I, bottom panel), despite a relatively constant apical to basal pMyoII ratio across the pouch AP axis (Figs. 2E, 3Fi-ii). Further, the increase in cell height between the lateral and medial domains becomes more pronounced as the disc grows in size (Supplementary Fig. 3B, C). Collectively, these observations suggest that multiple independent mechanisms likely play integrative roles in orchestrating cell height and ensuring robust tissue morphogenesis.

Along with these cytoskeletal dynamics, cell proliferation decreases toward the end of larval development, in agreement with previous studies^27,32,33^ (Fig. 2I, Supplementary Fig. 5). These observations of dynamic changes in cell-level processes such as contractility, cell-ECM adhesion, and proliferation suggest possible coordinated regulation between cell growth, proliferation, and the cell mechanics that define subcellular features of cells and the shape of the tissues.

### Key regulators of basal curvature include the ratio of apical to basal contractility, ECM stiffness and cell-ECM adhesion

We next tested several proposed mechanisms driving the flattening of the medial domain. From the normalized basal curvature (${\kappa }_{{basal}}$) calculations, we confirmed that ${\kappa }_{{basal}}$ is almost uniform at 72 h AEL larval stage but it becomes patterned with higher local ${\kappa }_{{basal}}$ at the lateral ends at 96 h AEL (Fig. 3 A-B”). To test hypothesized mechanisms that can generate medial flattening i.e. less curved than lateral ends (Fig. 3B”), we varied model parameters that correspond to perturbations in the cell cytoskeleton, the apical (${k}_{{api},{cont}}$) and basal (${k}_{{bas},{cont}}$) actomyosin contractility, the basal ECM stiffness (${k}_{{ecm},c}$), the membrane tension (${k}_{{memb},{basal}},{k}_{{memb},{lateral}},{L}{0}_{{memb},{lateral}},{L}{0}_{{memb},{basal}}$) and the Integrin-based adhesion between the cells and the basal ECM (${k}_{{adhB}}$) (Supplementary Figs. 6, 7). All simulations started with the same flat tissue shape as the initial condition. For comparison, our control simulation assumed homogeneous contractility across the AP axis on both apical and basal sides of individual cells with higher strength at the basal side while all other parameters remained constant. Under these conditions, the tissue evolved into a bent shape with a globally uniform curvature (Fig. 3C). For the in silico tests, parameters were patterned differently as step functions to increase or decrease the parameter values in the medial and lateral domains of the pouch (Fig. 3D-i, details in Supplementary Fig. 6, 7).

Next, we calculated the ratio of the average basal curvature of the lateral ends to the medial domain for both the experimental data and model simulations. This quantitative comparison (Fig. 3D-ii, Supplementary Fig. 8) revealed that the key regulators that can explain the flattening of the pouch’s medial domain are the ratio of apical-to-basal contractility (${k}_{{api},{cont}}/{k}_{{bas},{cont}}$), basal ECM stiffness (${k}_{{ecm},c}$) and the adhesion between pouch cells and the basal ECM (${k}_{{adhB}}$) (Fig. 3E i-iii, Supplementary Movies 1-3). The prescribed parameter profiles for these cases (Fig. 3E i-iii, top panel) resulted in medial tissue flattening compared to the lateral ends.

To corroborate our model results, we employed a quantification pipeline for fixed and stained samples for pMyoII, Col-IV, and $\beta$PS (Fig. 3F–H). We observed that late-stage wing imaginal discs (96 h AEL) have a nonuniform spatial patterning of pMyoII along the AP axis across both the apical and basal surfaces. In particular, pMyoII is more localized at the apical-medial and basal-lateral domains (Fig. 3F). This variation generates a decreasing trend in the ratio of the apical to basal pMyoII as one moves away from the center of the pouch along the AP axis. A similar patterning of the parameters ${k}_{{api},{cont}}$ and ${k}_{{bas},{cont}}$ can generate the flattening in the medial domain as observed in the experimental data (Fig. 3E-i). In fact, a similar patterning of pMyoII across the basal surface is reported for later staged discs (Fig. 3F’-ii).

Next, our model demonstrates that a graded stiffness of ECM across the AP axis for ECM surrounding pouch cells generates a ${\kappa }_{{basal}}$ profile similar to that of wing discs belonging to late developmental stages (96 h AEL). More specifically, a stiffer ECM at the pouch central region induced by increasing ${k}_{{ecm},c}$ in this region promotes flattening (Fig. 3E ii). The underlying reason why this phenomenon is observed in Fig. 3E-ii can be attributed to the dynamics between the model columnar cells under contractile force and the ECM. While the contractile forces originating from actomyosin contractile springs situated in each columnar cell promote tissue curvature, the equilibrium angle, $\pi$, prescribed to the bending potential energy function would lead to the ECM preferring a flat shape. By increasing the ECM stiffness (i.e. an increase in the resistance to stretching by increasing the linear spring coefficient) the ECM’s contribution to the overall model tissue shape becomes more pronounced. Therefore, the angles between nodes in the model ECM will tend toward the equilibrium value reducing the basal curvature. Our result suggests that under certain conditions, the ECM’s increased stiffness can promote a flatter basal curvature. Based on our observations, wing disc morphogenesis can be a joint effort of the locally stiffening ECM and the dynamics of actomyosin contractility. Apart from the graded ECM stiffness along the DV axis, the ECM appeared less taut at the basal region of squamous cells as compared to pouch columnar cells.

Our experimental results show increased Col-IV localization in the lateral bends of the wing disc (Fig. 3G, G’). This localization may result from two possible phenomena. First, it may result from spatially nonuniform local growth, which is greater in the z-direction (greater ECM thickness), increasing the local growth mismatch between the cell layer and the ECM in the planar AP direction. Alternatively, or additionally, this increased localization is due to greater basal contractility, shrinking the basal region resulting in a higher density of Col-IV compared to the flatter medial basal domain of the wing disc. Our data also shows higher Col-IV localization in the region surrounding the squamous epithelia, confirming the difference in stiffness of ECM surrounding the squamous and columnar cells. The latter finding is consistent with the results presented in previous work^22,23^. This result is also consistent with fold generation inhibited in the vicinity of a stiff ECM. Future experiments are required to estimate the mechanical properties of ECM to validate predictions made by the computational model.

Lastly, our model simulations show that increasing the adhesion strength between the ECM and basal surface of columnar cells at the lateral domains compared to the medial domain flattens the tissue (Fig. 3E-iv). Similar to the input parameter profile, we observe high Integrin localization in the lateral domains of the pouch compared to the medial region for discs belonging to late developmental stages (Fig. 3H, H’-ii).

In summary, our experimental data, together with the simulation results, suggest that nonhomogeneous actomyosin contractility, basal ECM stiffness, and cell-ECM adhesion all can generate a tissue shape with non-uniform curvature where the medial section is flat. These correlations in non-homogeneous spatial patterns suggest the possibility of mechanistic redundancy and crosstalk between multiple cellular processes. A high degree of correlation between $\beta$PS and pMyoII at the pouch basal surface across multiple developmental stages (Fig. 2F) suggests that spatiotemporal regulation in one of the cytoskeletal regulators affects the patterning of another.

### A balance in the patterning of forces generated by actomyosin contractility, basal cell tension, and cytoplasmic pressure maintains a spatial profile in cell height

**Fig. 4:**
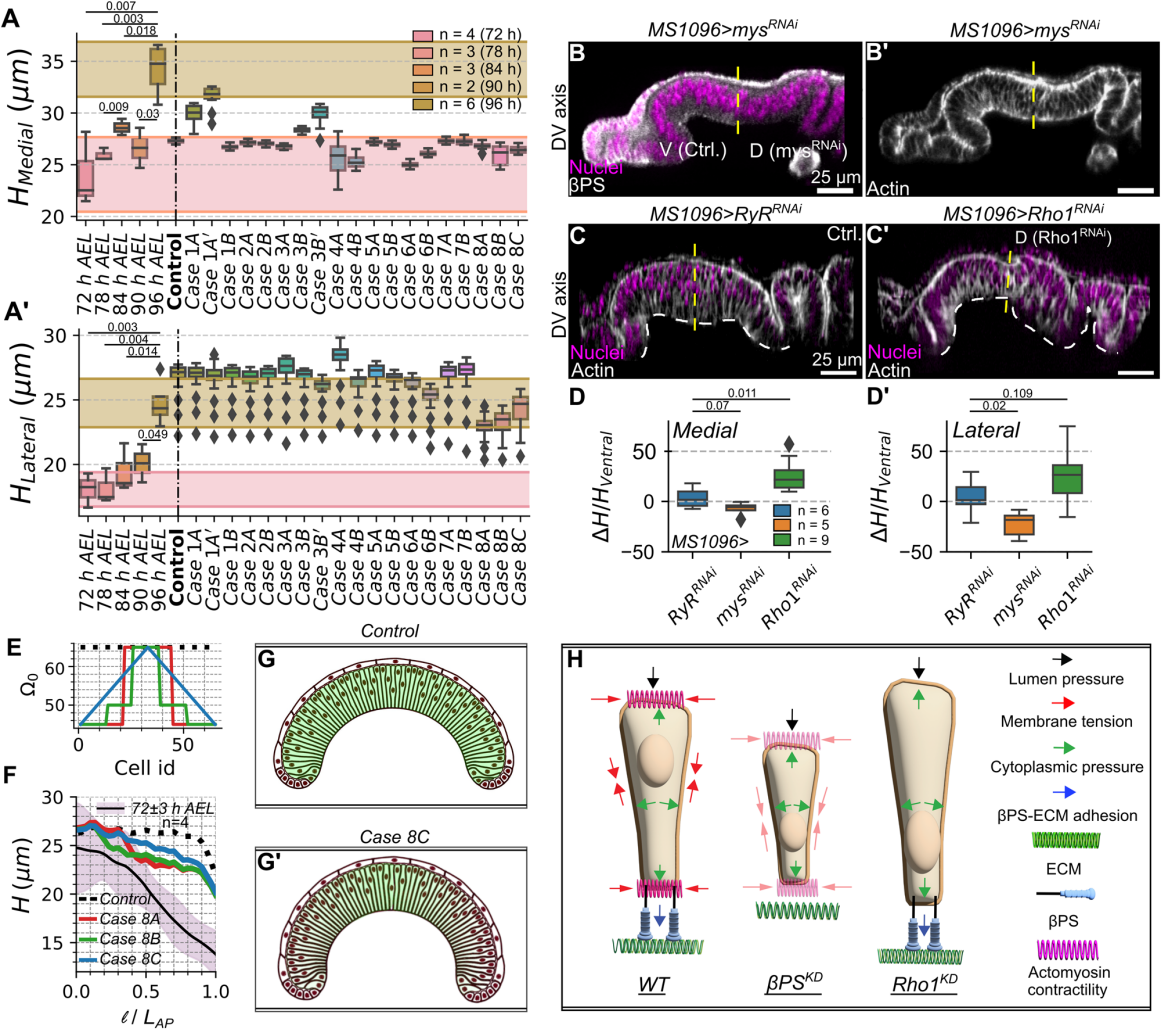
A balance in actomyosin-mediated contractility, surface tension, Integrin-ECM adhesion, and cell pressure patterns tissue thickness. **A**, **A’** Quantification of medial and lateral heights for discs belonging to 72-96 h AEL larval stages and model simulations (Reference parameter profiles are found in Supplementary Fig. 6). The error bars represent points within the 1.5 interquartile range of the lower and upper quartile, and diamonds represent the values outside this range, respectively. Shaded regions represent the standard deviation of the 72 h and 96 h experimental data. **B-B’** $\beta$-Integrin was knocked down in the dorsal compartment of the wing (*MS1096-Gal4 x UAS-mys*^*RNAi*^*)*. Visualization of the cross-section along the DV axis. **C-C’** pMyoII was knocked down in the dorsal compartment of the wing disc (*MS1096-Gal4* x *UAS-Rho1*^*RNAi*^). Control for the experiment has been generated by crossing the same Gal4 driver with a *UAS-RyR*^*RNAi*^ line, previously validated as a negative control for wing morphology. **D, D’** Quantification of medial and lateral heights for the $\beta$-Integrin and pMyoII genetic perturbations. **E** The target cell volume (${\varOmega }_{0}$) in the Lagrange Multiplier was varied across the DV axis for pouch cells. Variations of parameter profiles are included. **F** Plot showing the height of discs at 96 h AEL. The black solid line indicates the average experimental height. The shaded region represents the standard deviation across multiple samples. Lines in green and blue represent the control and the case where the ${\varOmega }_{0}$ was patterned. **G**, **G’** Model simulation output for (**F**). **H** A schematic summary of height regulation in pouch cells. Here, WT and KD stand for wild-type and knockdown, respectively.

As described previously, the average tissue height increases as the discs age from 72 h to 96 h of larval development (Fig. 2) across both medial and lateral domains of the pouch (Fig. 4A, A’). Our experimental data, which is validated by previous studies^9^, also shows a gradient in the patterning of cell height across the AP axis (Fig. 2, Supplementary Fig. 3). Cells in the central region of the pouch are more elongated as compared to cells in the lateral ends (Fig. 4A, A’, Supplementary Fig. 3D).

To test the importance of cytoskeletal regulation in defining the tissue height, we analyzed cell height for the simulation outputs generated during the different model scenarios (Supplementary Figs 6, 7). Our model identifies apical and basal contractility (${k}_{{api},{cont}}$ and ${k}_{{bas},{cont}}$, respectively) and basal cell tension (${k}_{{memb},{basal}},{L}{0}_{{memb},{basal}}$) as crucial regulators of cell height in the middle domain of the tissue (Fig. 4A, A’). These parameters were patterned across the AP axis as previously described. An increase in the ${k}_{{api},{cont}}/{k}_{{bas},{cont}}$ ratio within the pouch medial domain causes the cells to elongate (Fig. 4A, Supplementary Fig. 9). Moreover, our model also suggests that an increase in the contractility values in both the apical and basal surfaces of the pouch while (1) keeping the ratio constant and (2) increasing the ratio led to an increase in average tissue thickness (Supplementary Fig. 9). This increase in cell height is limited to the medial pouch domain where the increase in the apical and basal contractility is introduced. (Additional details regarding the variation in the ${k}_{{api},{cont}}/{k}_{{bas},{cont}}$ ratio can be found in the Supplementary Section 2.7). Finally, our model suggests that modulating the basal tension of the cells changes tissue thickness. In particular, lowering basal tension in the center of the pouch by increasing $L{0}_{{memb},{basal}}$ shrinks the height in the medial domain (Fig. 4A, Case 6B).

Next, we analyzed experimental perturbations to test the role of the aforementioned contractility and basal tension parameters on basal curvature and height maintenance. First, we perturbed tension in the basal surface of columnar cells by RNAi-mediated downregulation of *myospheroid* (*mys*), a $\beta$-subunit of the Integrin dimer (Fig. 4B). Perturbation in the cell-ECM adhesion through downregulation of *mys* reduced basal $\beta$PS levels and cell height. Notably, the cell height reduction was pronounced across the lateral section of the pouch (Fig. 4D, D’) as denoted by the p-values of the student t-test for the comparison of height (H) between RyR^RNAi^ control and mys^RNAi^ (p-value of 0.06 and <0.05 in the lateral and medial sections, respectively). Interestingly, the loss of Integrins also caused a loss in basal expression of pMyoII, suggesting a positive regulation of pMyoII through Integrin localization (Supplementary Fig. 10E, E’). Next, we varied the apical to basal contractility ratio, associated with the ${k}_{{api},{cont}}/{k}_{{bas},{cont}}$ ratio from simulations, in the dorsal compartment of the pouch by expressing Rho1^RNAi^ using an MS-1096 Gal4 driver (Fig. 4C, C’). Inhibiting Rho1 increases cell height and inhibits basal curvature (Fig. 4D, D’, Supplementary Fig. 10).

Although the computational model scenarios identified the parameters that can explain the control of cell height within the medial domain of the tissue, it remained unclear if they were sufficient to generate the gradient in cell height across the AP axis as observed in the experimental data (Fig. 4A, A’). Except for the variation of ${k}_{{api},{cont}}/{k}_{{bas},{cont}}$, the other case study simulations did not generate the same difference in average cell heights compared to the experimental data. Since the loss of actomyosin contractility in Rho1 genetic perturbations increased cell height, we hypothesize that cytoplasmic pressure within the cell must be one of the factors causing the tissue to increase in volume with reduced cortical actomyosin tension. Moreover, the increase in cell height was non-uniform and increased more in the medial than the lateral region (Fig. 4D, D’). This suggests that a non-uniform distribution of cell pressure across the AP axis may potentially explain the varying degrees of cellular height changes across the AP axis when Rho1 is inhibited. Testing this hypothesized spatial gradient in cell pressure across the tissue requires future experiments. In our simulations, we varied cell pressure by changing the control volumes (${\varOmega }_{0}$) of cells such that ${\varOmega }_{0}$ decreases away from the center of the pouch (Fig. 4E-G’, Supplementary Fig. 11). A patterning of ${\varOmega }_{0}$ increased the gradient in cell height, as is observed within the experimental data (Fig. 4F, G’, Supplementary Movie 4). Hence, the cell height in the lateral domains is more sensitive to variations in cell volume, while the cell height in the medial domain of the tissue is more sensitive to non-homogenous patterning of actomyosin contractility. In conclusion, our computational model simulations show that changing the relative ratio between apical and basal actomyosin contractility by modifying ${k}_{{api},{cont}}$ and ${k}_{{bas},{cont}}$, varying the tension in the basal membrane by changing ${k}_{{memb},{basal}}$ or patterning cell pressure^34^ through the target volume term (${\varOmega }_{0}$) can impact local tissue height (Fig. 4H).

### The relative levels of apical contractility and basal surface tension determine nuclear positioning in pseudostratified epithelial cells

**Fig. 5:**
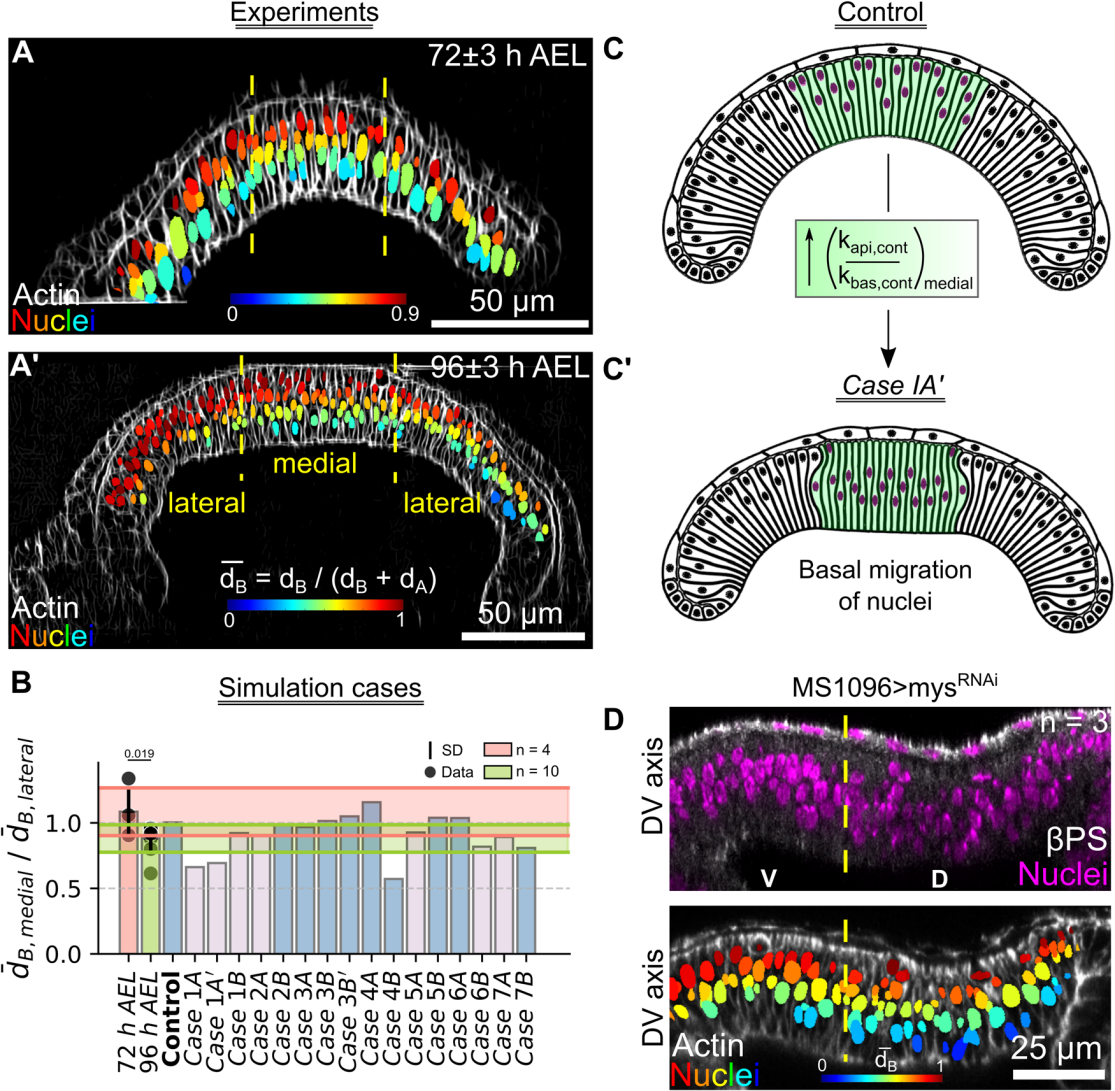
Higher levels of apicocentral contractility results in a basal bias of the position of nuclei. **A-A’** Optical sections along the AP axis for discs belonging to 72 and 96 h AEL were used to mimic the patterning of contractility in the model simulations. Nuclei have been color-coded with respect to their distance from the basal surface ($\bar{{d}_{B}}$). **B** Bar graph visualizing ratio of $\bar{{d}_{B}}$ between the medial and lateral pouch domains for the experimental data (orange and green bars) and the model simulations (pink and blue bars). **C** Nuclear positioning in a uniformly patterned actomyosin profile is distributed uniformly within each columnar cell. **C’** A non-uniform actomyosin patterning generates a flatter midsection, resulting in a shift of nuclear positions toward the basal side in the midsection. **D** (Top panel) Optical section along the DV axis for pouch expressing mys^RNAi^ predominantly in the dorsal compartment using an *MS1096-Gal4* driver. Fluorescent labels have been indicated as an inset within the plot. (Bottom panel) Nuclei within the genetically perturbed cross-section have been color-coded based on $\bar{{d}_{B}}$, where 0 and 1 denote a more basal or apical location, respectively.

Next, we analyzed the positioning of nuclei within pouch cells of wing imaginal discs from 72 h and 96 h AEL (Fig. 5 A-A’). In the initial stages, the distribution of nuclear positioning is uniform across the AP cross-section (Fig. 5A, B). However, with age, nonmitotic nuclei in the medial domain are more basally located than those in their lateral counterparts (Fig. 5A’, B). To study the relative importance of individual cytoskeletal regulation components, we measured nuclei positioning across the pouch medial and lateral domains for all of the different simulation case scenarios performed in the previous sections (Figs 2, 3). We define $\overline{{d}_{B}}$ as the relative distance of the nuclei from the basal surface (Fig. 5A’). The ratio of $\bar{{d}_{B}}$ across the medial to lateral domains was computed and compared with the experimental data (Fig. 5B).

In the case of a uniform patterning of the actomyosin profile, the relative nuclear positioning along the apicobasal axis shows marginal differences across the whole wing disc (Fig. 5B, C). A higher ratio of apical to basal contractility $({k}_{{api},{cont}}/{k}_{{bas},{cont}})$ in the medial domain (Case 1 A’), where the apical contractility is comparable to the basal contractility, pushed the nuclei to the basal surface of the pouch (Fig. 5B, C, C’). The simulated shift in contractility is similar to the experimentally reported change of pMyoII (Fig. 2). Based on our experimental observations and predictions generated by our model, we initially targeted the inhibition of actomyosin contractility in the dorsal compartment of the wing disc by inhibiting Rho1. This inhibition resulted in the migration of columnar cell nuclei towards the basal surface, as detailed in Supplementary Fig. 12. Thus, Rho1 mediates actomyosin contractility in maintaining the proper nuclear arrangement within wing discs.

Further, our model simulation results suggest a patterning of basal tension (Case 6B) and the adhesion of cells with the ECM (Case 7 A) (Fig. 3D-i, Supplementary Fig. 6), which can also impact nuclear positioning (Fig. 5B). To validate this experimentally, we knocked down *myospheroid* (*mys*) predominantly in the dorsal compartment of the wing disc. As a result, we observed more migration of nuclei to the basal surface of cells in the dorsal compartment compared to the ventral side, semi-quantitatively validating the model predictions (Fig. 5D’).

Both our experimental data and computational simulations suggest that nuclei are shifted basally in the center of the wing disc as development progresses. In summary, our results in this section indicate that the apical-basal polarization of myosin and the patterning of basal tension and cell-ECM adhesion influence nuclear positioning within the epithelium.

#### Increasing cell proliferation enhances local basal curvature

**Fig. 6:**
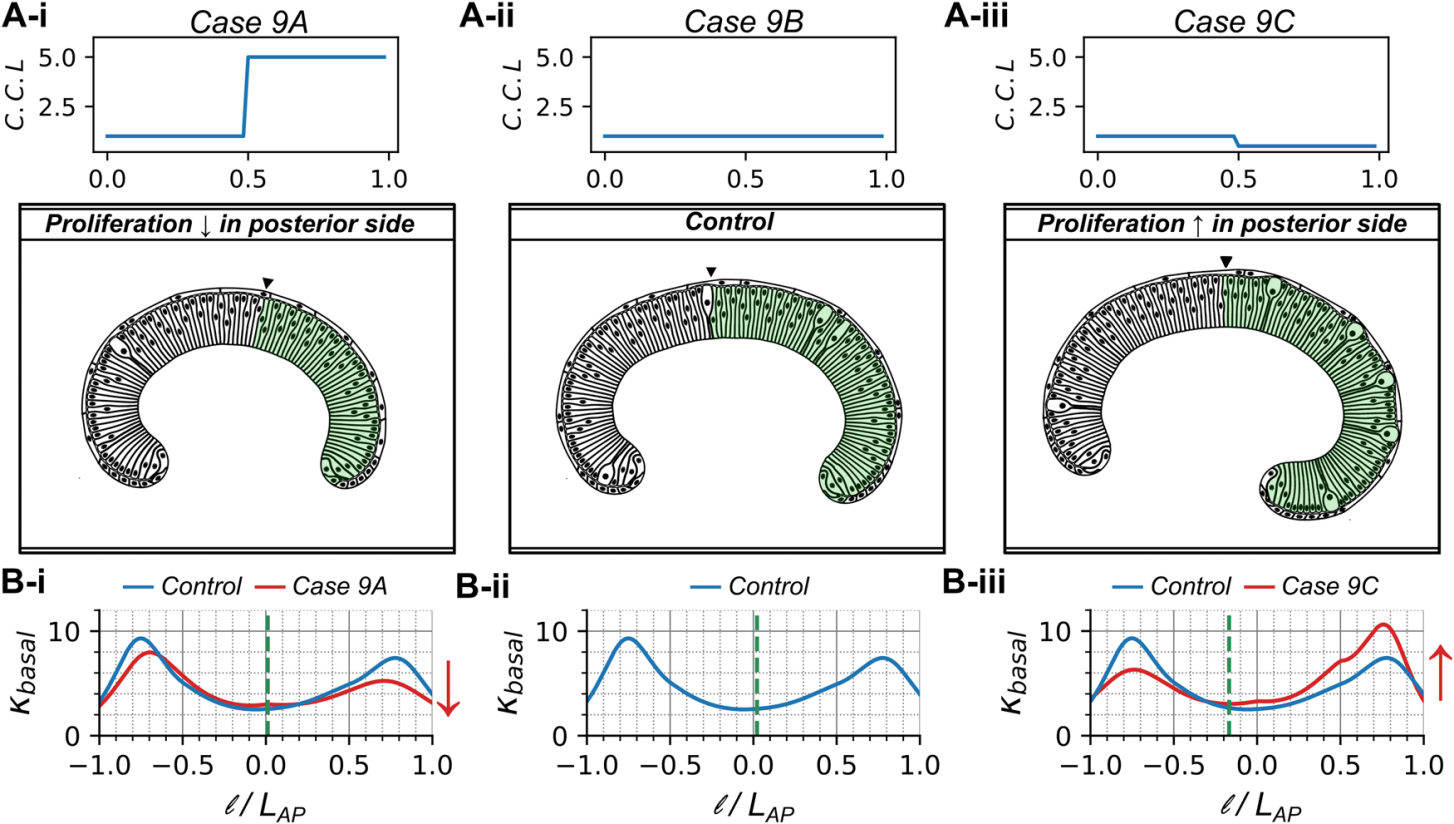
Stimulating cell proliferation increases tissue curvature. Comparison of the local basal curvature between simulated wing disc cross sections with compartment-specific variation of proliferation rates. These simulations assumed increased actomyosin contractility on the basal region of the columnar cells. **A i-iii** Cell cycle duration in the posterior compartment is varied compared to the constant anterior compartment. **B i-iii** Plots quantifying and comparing the distribution of local basal curvature corresponding to cases (A i-iii). Red arrows denote a decrease or increase in curvature.

To study how cell proliferation impacts tissue geometry along the AP axis, we incorporated the proliferation of the columnar cells in our computational model (Fig. 6). For these simulation scenarios, the initial geometry of the wing disc specified in simulations reflects the curvature observed in experimental wing discs at approximately 72 h AEL. We varied cell proliferation rates by increasing or decreasing the cell cycle length (C.C.L.) in the posterior compartment of the wing imaginal disc (Fig. 6Ai-iii, Supplementary Movies 5-7). Note that an increase in C.C.L. induces a decrease in proliferation, while a decrease in C.C.L. increases proliferation. In particular, we set the posterior-to-anterior cell cycle length ratio of 400% for Fig. 6A-i or 50% for Fig. 6A-iii, which was compared to the “wild type” control simulation that exhibits a spatially homogeneous cell division rate (Fig. 6A-ii). A decrease in proliferation reduced basal curvature and cell height (Fig. 6A-i, B-i, Supplementary Fig. 15A, B). In contrast, increasing cell proliferation in the posterior compartment increased the local basal curvature (Fig. 6B-iii). In other words, an increase in proliferation rate results in increased inwards bending of the pouch (Fig. 6B-iii, Supplementary Movie 7). Finally, cell height slightly increases in a compartment-specific manner if cell proliferation is increased (Supplementary Fig. 15A, B).

### Targeting proliferation via different signaling pathways results in distinctive tissue morphologies

**Fig. 7:**
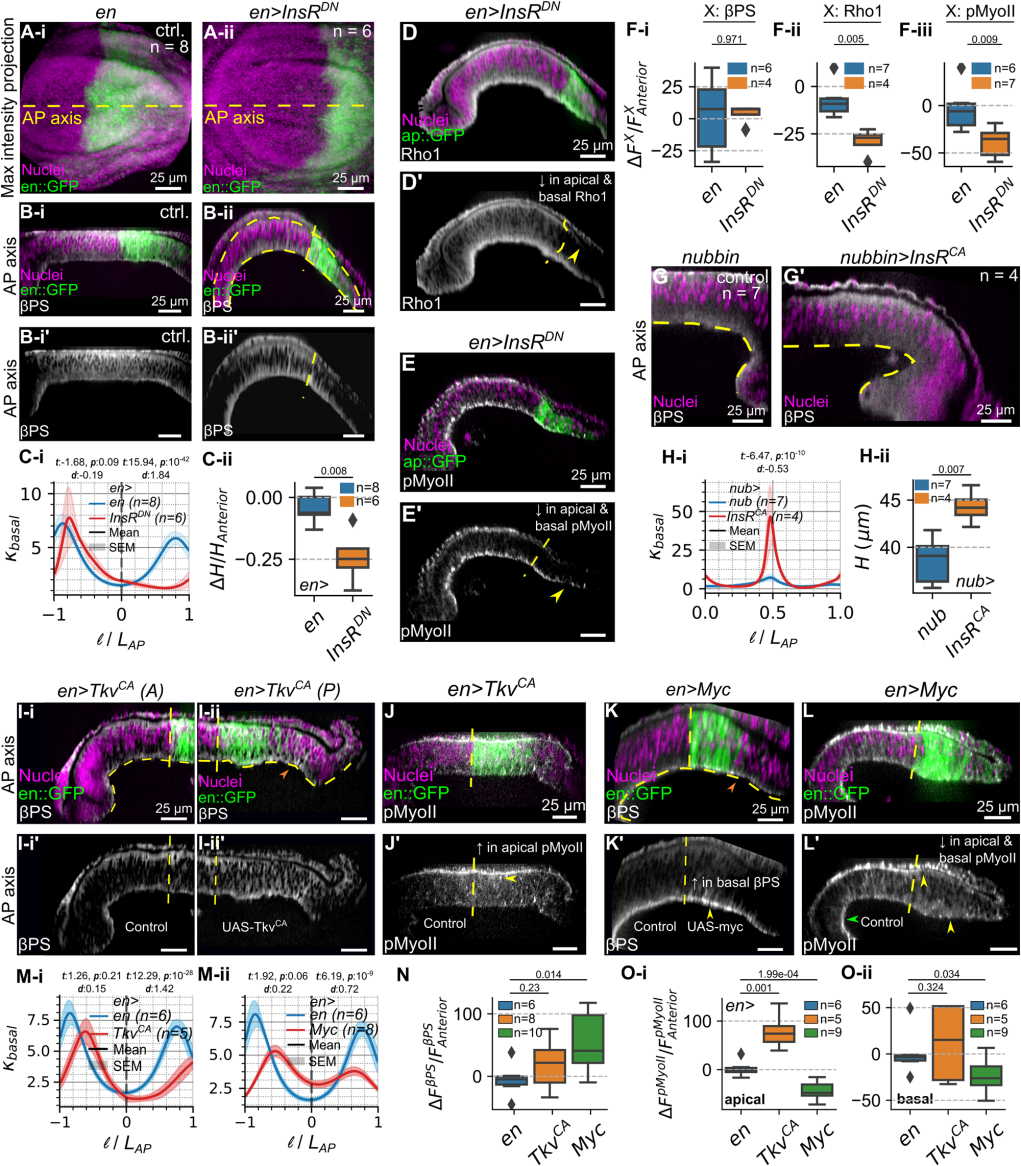
Stimulating cell proliferation by different growth-promoting pathways results in two distinct phenotypes. **A** Maximum intensity projection of **(i-i’)** control and **(ii-ii’)** samples expressing dominant negative insulin receptor InsR^DN^ in the posterior compartment driven by en-Gal4. Proliferating cells are marked by PH3. **B** Cross-section along AP-axis for **(i-i’)** control and **(ii-ii’)** InsR^DN^. **C** Plots quantifying the **(i)** basal curvature profile and **(ii)** difference in average cell heights between the perturbed posterior and control anterior compartment (ΔH). ΔH is normalized by the average height of the control anterior compartment. **D**, **E** (**D-D’**) Rho1 and (**E-E’**) pMyoII expression across the AP axis for pouches expressing InsR^DN^ in the posterior compartment. **F** Quantification of the differences in average fluorescence intensities (ΔF^X^) of (i) x: βPS, (ii) x: Rho1 and (iii) x: pMyoII between the posterior and anterior compartment for *en>Ins*^*DN*^ in the wing disc posterior compartment. ΔF^X^ is further normalized by average fluorescence intensity of x in the control anterior compartment (**G-G’**) Cross section along AP axis (medial to lateral, half the pouch) for discs expressing nubbin Gal4 driver and nubbin>InsR^CA^. **H** Quantification of (i) basal curvature and cell height (ii) for disc expressing *InsR*^*CA*^. **I-J’** Optical section along the dorsal-ventral compartment boundary taken in the anterior (left) and posterior (right) compartments of the wing disc (**I-I’**) expressing a constitutively active form of Tkv receptor (*Tkv*^*CA*^) and (**J-J’**) AP cross sections of wing discs expressing *en>Tkv*^*CA*^ stained with pMyoII. Fluorescent labels have been indicated within the figure. **K-K’** overexpressing *Myc* in the posterior compartment. Scale bars correspond to 25 $\mu m$ unless indicated. **L-L’** AP cross sections of wing discs expressing *en>Myc* stained with pMyoII. Fluorescent labels have been indicated within the figures. **Mi-ii** Quantification of local basal curvature $({\kappa }_{{basal}})$ for discs overexpressing *Tkv*^*CA*^ and *Myc*. **N** Boxplot visualizing differences in average βPS (ΔβPS) expression between posterior (perturbation) and anterior (control) compartments on en>*Tkv*^*CA*^ or en>*Myc*. ΔβPS was normalized by average fluorescence intensity computed within the anterior compartment. **O** Similar quantification for pMyoII was carried out at the pouch apical and basal surfaces for *en>Tkv*^*CA*^ and *en>Myc*.

To qualitatively validate the model simulation’s predictions, we investigated the impact of genetically perturbing the growth signaling pathways in a compartment-specific fashion on the morphology of the wing disc pouch. First, we inhibited proliferation in the posterior compartment by expressing the dominant-negative (DN) form of the *Drosophila* insulin receptor (InsR^DN^) in the posterior compartment (Fig. 7A). We confirmed that downregulation of insulin signaling activity through the expression of InsR^DN^ reduced the number of mitotic cells^35^ (Supplementary Fig. 13). Strikingly, inhibiting insulin signaling in the posterior compartment reduced tissue bending (Fig. 7B, C-i). Additionally, we observed that the tissue height decreased (Fig. 7C-ii). We also expressed constitutively active forms of insulin receptors (InsR^CA^) in the whole pouch. We found that such discs showed a significant increase in the inwards bending (Fig. 7G, G’, H-i) and tissue height (Fig. 7H-ii).

Insulin signaling can also regulate contractility via a PI3K-mediated activation of pMyoII^36,37^. Consequently, we assessed if there were changes in the spatial patterning of cytoskeletal regulators such as Integrin or Rho1, an upstream activator of pMyoII. We found that the relative Integrin levels measured across multiple samples did not change significantly (Fig. 7F-i). However, there is a statistically significant decrease in Rho1 (Fig. 7D, D’, F-ii). We also report a compartment-specific reduction in pMyoII levels upon expression of InsR^DN^, suggesting that insulin signaling interacts with Rho GTPases to regulate contractility within the tissue (Fig. 7F-iii). As a loss in contractility can also result in a loss of bending, it is difficult to decouple the individual role of proliferation in generating bending across the tissue.

To further test how growth regulators impact tissue geometry, we studied the effect of downregulating *mTOR*, a direct regulator of the cell cycle and cellular growth in the wing disc^38^ (Supplementary Fig. 14). First, we expressed the dominant negative form of *mTOR* in the posterior compartment with engrailed*-Gal4*. However, the progeny was lethal. Hence, we expressed *mTOR*^*DN*^ in the dorsal compartment of the wing disc with *apterous-Gal4*. The expression of *mTOR*^*DN*^ resulted in the loss of bending in a compartment-specific manner compared to the control Gal4 driver (Supplementary Fig. 14B-ii, E-ii). We also report a decrease in cell height upon expression of *mTOR*^*DN*^ (Supplementary Fig. 14E-i). Finally, while we did not observe changes in βPS (Supplementary Fig. 14D-i), we report a compartment-specific decrease in both basal Rho and basal pMyoII along the AP axis upon expression of *ap* > *mTOR*^*DN*^ in the dorsal compartment (Supplementary Fig. 14D-ii).

Similar to the findings from our computational model, our experimental data captures the trends predicted by our model, that an overall increase or decrease in cell proliferation can result in an increase and decrease of both cell height and basal curvature, respectively. Additionally, quantitative analysis of the experimental data shows that changing proliferation through InsR or *mTOR* can also cause changes in cytoskeletal regulators such as pMyoII. Cytoskeletal regulation and cell proliferation are correlated, which makes it impossible to decouple the relative importance of the two through experiments. As a result, we ran additional simulations where we decreased both cell proliferation and basal contractility in half of the pouch (Supplementary Fig. 15C, D). The simulations qualitatively recapitulate the tissue flattening observed within the InsR and *mTOR* genetically perturbed samples. Moreover, our simulations show that decreased basal contractility further decreases the tissue’s ability to fold. However, by genetically downregulating mTOR in an 84 hr AEL wing discs, we observed that the tissue geometry for loss of mTOR seems similar to the control during the early stages of development, suggesting that there are no noticeable initial alterations in tissue morphology (Supplementary Fig. 16). Based on this evidence, it appears that our simplifying assumptions for different developmental stages are suitable for the present study.

To further test the effect of increasing cell proliferation on tissue morphology, we perturbed other growth signaling pathways. We next overexpressed the constitutively active form of DPP receptors, Tkv^CA^, in the posterior compartment^39^ (Fig. 7I-I’). Finally, we also overexpressed Myc in the posterior compartment (Fig. 7K-K’), since it is a known regulator of cellular growth in developing *Drosophila* wing imaginal discs^40^. Since both constitutive active Dpp signaling and Myc stimulate the overgrowth phenotype in the pouch^28,41^, an increase in local basal curvature upon expression of Tkv^CA^ and Myc was expected. Surprisingly and in contrast to perturbations in insulin signaling, both the Tkv^CA^ and Myc overexpression conditions decreased inward bending in the posterior compartment (Fig. 7Mi-ii).

Based on these counter-intuitive results, we reasoned that both Tkv^CA^ and Myc may also impact the relative concentration levels of cytoskeletal regulators. Hence, we next explored how perturbing cell proliferation through the expression of Tkv^CA^ and Myc affects cytoskeletal regulation. In particular, we examined changes in cell-ECM adhesion (βPS) and actomyosin contractility (Rho1, pMyoII) (Fig. 7I-L, Supplementary Fig. 17,18). We found no statistically significant change in the expression of βPS upon expression of Tkv^CA^ in the posterior compartment when compared to the internal control, which is the anterior compartment (Fig. 7I, N). However, an increase in both Rho1 and pMyoII was observed in the posterior compartment when Tkv^CA^ was expressed therein (Fig. 7J, J’, Supplementary Fig. 17). More specifically, there is a statistically significant increase in pMyoII in the apical surface of the posterior compartment where Tkv^CA^ was expressed (Fig. 7Oi-ii). Conversely, overexpression of Myc in the posterior compartment resulted in a statistically significant increase in βPS levels (Fig. 7K-K’, N). Nevertheless, we documented a decrease in Rho1 and pMyoII at the pouch lateral domain upon Myc overexpression (Fig. 7L, L’, Oi-ii, Supplementary Fig. 18). Additionally, as a check for our Rho1 antibody staining studies, we also tested Rho1 biosensors and observed similar expressions proving that the Rho1 antibody staining likely provide a reasonable proxy for activity. (Supplementary Fig. 21). However, in the future additional studies will be needed to understand how growth regulators control Rho1 dynamics and activity. In the subsequent section, we computationally validate whether the changes as mentioned earlier are capable of rescuing the increased epithelial bending associated with enhanced cell proliferation.

### A dual regulation of proliferation and mechanical regulators causes a loss in tissue bending

**Fig. 8:**
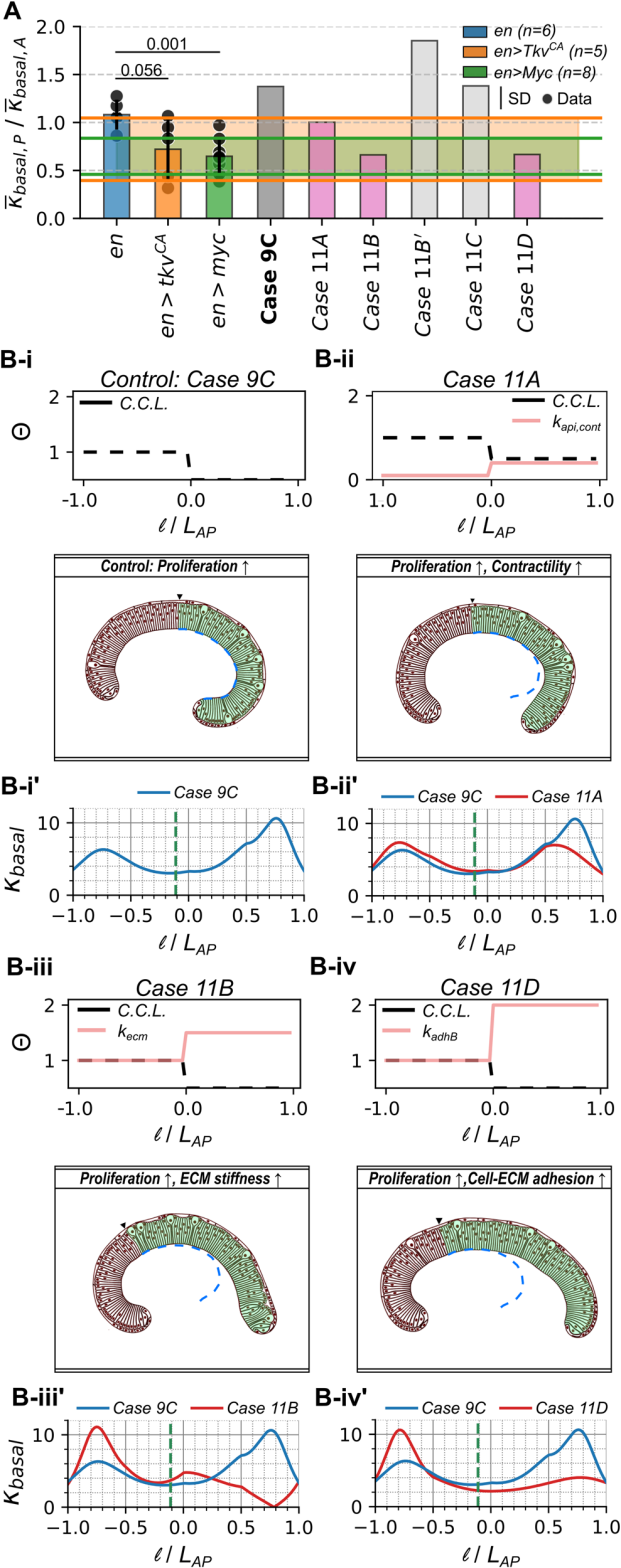
Effects of increased proliferation can be buffered by concomitant changes in cytoskeletal regulation. **A** Comparison of ratio of average basal curvature in the posterior to anterior compartment in Fig. 7 (I-K) and for (**B i-iv**)**. B i-iv** The simulated cases closely replicate shape changes in the form of ${\kappa }_{{basal}}$ reduction as observed in *Tkv*^*CA*^ and *Myc* genetic perturbations. Two perturbations were performed in these simulations. First, cell proliferation was increased in the posterior (P) compartment of the model wing disc compared to the anterior (**A**) compartment. Second, parameters controlling cytoskeletal regulators were varied in the region of increased proliferation. **B i’-iv’** Quantification of the basal curvature for each case corresponding to (**B i-iv**).

To resolve the apparent contradiction of the prediction that increases in cell proliferation enhance basal curvature, we ran additional simulations where we changed both cell proliferation and cell mechanics. More specifically, we divided the in silico tissue into anterior and posterior compartments and computationally increased proliferation in one half of the pouch by 50% (posterior side) and then systematically varied the parameters controlling cytoskeletal regulators in the region in the region of perturbation (Fig. 8A, B). Based on a comparison of the ratio of average basal curvature of the posterior to the anterior compartment (Fig. 8A), we identified that an increase in basal cell-ECM adhesion (${k}_{{adhB}}$, 50% increase in the posterior compartment), ECM stiffness (${k}_{{ecm},c}$, 100% increase in the posterior compartment) or apical actomyosin contractility (${k}_{{api},{cont}}$, 300% increase in the posterior compartment) (Fig. 8Bii-iv) could effectively counter the increase in bending upon increased proliferation (Fig. 8B-i) (see Supplementary Movies 7-10). Similar to the Tkv and Myc perturbation data, our simulations capture the trend of loss in tissue bending in the compartment of perturbation.

Through a comparison between experimental data on cytoskeletal regulation in *en>Tkv*^*CA*^ genetic perturbations (Fig. 7I-J’) and the best simulation cases identified (Fig. 8A), we conclude that an increase in apical to basal ratio of pMyoII (Fig. 7O) serves as one of the essential driving forces for flattening of the pouch basal surface despite of an increase in proliferation. An increase in apical pMyoII can be attributed to an increase in Rho, as observed in *ap>Tkv*^*CA*^ genetic perturbations and supported by previous studies^9^. Further, downregulation of *Tkv* in the posterior compartment reduced both Rho1 levels and basal curvature (Supplementary Fig. 17). This supports a mechanism where Dpp regulates pMyoII through Rho GTPases to control contractility^9^. In addition, Rho1-mediated phosphorylation of pMyoII can increase contraction along the apical and basal surfaces of the pouch, and this further promotes the local increase in cell height. Hence, the analysis of the experimental data also suggests an increase in cell height upon expression of *Tkv*^*CA*^ in the wing imaginal disc^9^ (Supplementary Fig. 17G). This agrees with simulations that show an increase in columnar cell height upon an increase in the apical to basal contractility ratio (Fig. 4A-A”).

An analysis of experimental data on cytoskeletal regulation in *en>Myc* genetic perturbation reveals that an increase in βPS mediated cell-ECM adhesion (Fig. 7K, N) and a decrease in pMyoII mediated basal contractility (Fig. 7L, O) can result in pouch flattening despite the concurrent increase in proliferation. Currently, there is limited experimental data on how Myc regulates cytoskeletal dynamics during development. To investigate the pMyoII mechanism, we carried out a Rho antibody staining, revealing a similar decrease in Rho levels as observed for pMyoII upon Myc overexpression (Supplementary Fig. 18). Additionally, previous studies have also found that c-Myc suppresses the activity of RhoA affecting Actin dynamics in cancer cells^42^. Previous literature findings also demonstrate a strong correlation between ITGA1 (Integrin subunit alpha 1) and c-Myc expression in colorectal cancer cells^43^. This suggests that the changes in βPS observed in *en>Myc* genetic perturbations may be generalized to multiple biological contexts.

Our model also predicts that an increase in ECM stiffness by increasing ${k}_{{ecm},c}$ can reduce the folding of tissue along the AP axis (Fig. 8B-iii). One of the scenarios where the stiffness of the tissue can increase is when the rate of ECM production is less than the growth rate in the cellular layer. The effect of Tkv and Myc mutations on ECM stiffness remains a point for future inquiry.

In summary, our experimental data, along with computational simulations, reveal that a balance in cell proliferation and differential patterning of cytoskeletal regulators determines the overall shape of the wing imaginal disc. A temporal change in both facilitates shape changes during morphogenesis, and different growth-promoting pathways lead to divergent changes in cytoskeletal regulation. This flexibility leads to the ability to tune both overall organ shape and size.

## Discussion

One of the most important unresolved problems in developmental biology is how tissue morphogenesis is coordinated through the regulation of both cell proliferation and the cellular cytoskeleton. The relative contributions of cell proliferation and cell mechanics to the final morphology of an organ are context-dependent. In some situations, proliferation and morphogenesis are separated into nonoverlapping temporal stages. For example, cell proliferation halts before gastrulation begins in the fly^44^. This strategy is likely important when developmental speed is of the essence, and the tissue cannot reach a pseudo-steady state before a new developmental event occurs. In other contexts, proliferation and cell shape changes occur simultaneously, as in the developing vertebrate optic cup^45^.

In this paper, a multi-scale (in space) SCE computational model, closely integrated with experiments, was used to quantitatively investigate the emergent features of tissue morphogenesis. The biologically calibrated SCE model describing both tissue growth and morphogenesis incorporates the spatial patterning of fundamental subcellular properties (Fig. 1). Additionally, the model implements for the first time the dynamics of interkinetic nuclear migration within the simulated pseudostratified epithelium. This includes the basal to apical motion of the nucleus, mitotic rounding, and cell division dynamics (Fig. 1). Key characteristics of global tissue architecture, such as the local curvature of the basal wing disc epithelium, cell height, and nuclear positioning, serve as metrics for model calibration. Our experiments show how these physical features are jointly regulated through spatiotemporal dynamics in the localization of pMyoII, $\beta$-Integrin, and ECM stiffness (Fig. 2). As the disc grows in size, there are progressive changes in the patterning of key subcellular features such as actomyosin contractility (Fig. 2).

Here, we show that the patterning of actomyosin contractility, ECM stiffness and cell-ECM adhesion along the AP axis near the DV compartment boundary are vital regulators of tissue shape changes, specifically driving the flattening of the midsection (Fig. 3) and increasing the cell height (Fig. 4). Moreover, changes in cell shape across the AP axis pattern the positioning of nuclei (Fig. 5). The predictions made by the model simulations agree with the observed changes in contractility and cell-ECM adhesion during wing disc morphogenesis. In fact, they are validated through perturbations of either actomyosin contractility (Rho1^RNAi^) or basal cell tension and cell-ECM adhesion (mys^RNAi^).

Through multiple case studies, our computational model identifies the primary regulators of cell height to be the apical to basal ratio of actomyosin contractility, basal membrane tension, and cell volume (Fig. 4). In particular, we show that variation in pressure (modeled as target volume) is sufficient to generate a gradient of cell height across the pouch AP axis^34^. To further validate the model’s predictions, we demonstrate that dysregulation in either actomyosin contractility or cell-ECM adhesion through *Rho* and *mys* perturbations cause severe morphological defects (Fig. 4B-E).

Our experimental studies also show that changes in proliferation using multiple different pathways also substantially regulate the cytoskeleton. Using the computational model simulations, we show that a decrease or increase in proliferation alone can decrease or increase the tissue basal curvature and height, respectively. We experimentally validated these findings by perturbing *InsR* and *mTOR* signaling in specific compartments. Downregulation of either pathway caused a reduction in basal curvature and tissue height (Fig. 7C). However, perturbing cell proliferation via other cell signaling pathways of growth also cause changes in cytoskeletal regulation. For example, inhibition of *InsR* signaling causes a reduction in pMyoII through a *Rho* signaling pathway (Fig. 7D, E).

**Fig. 9:**
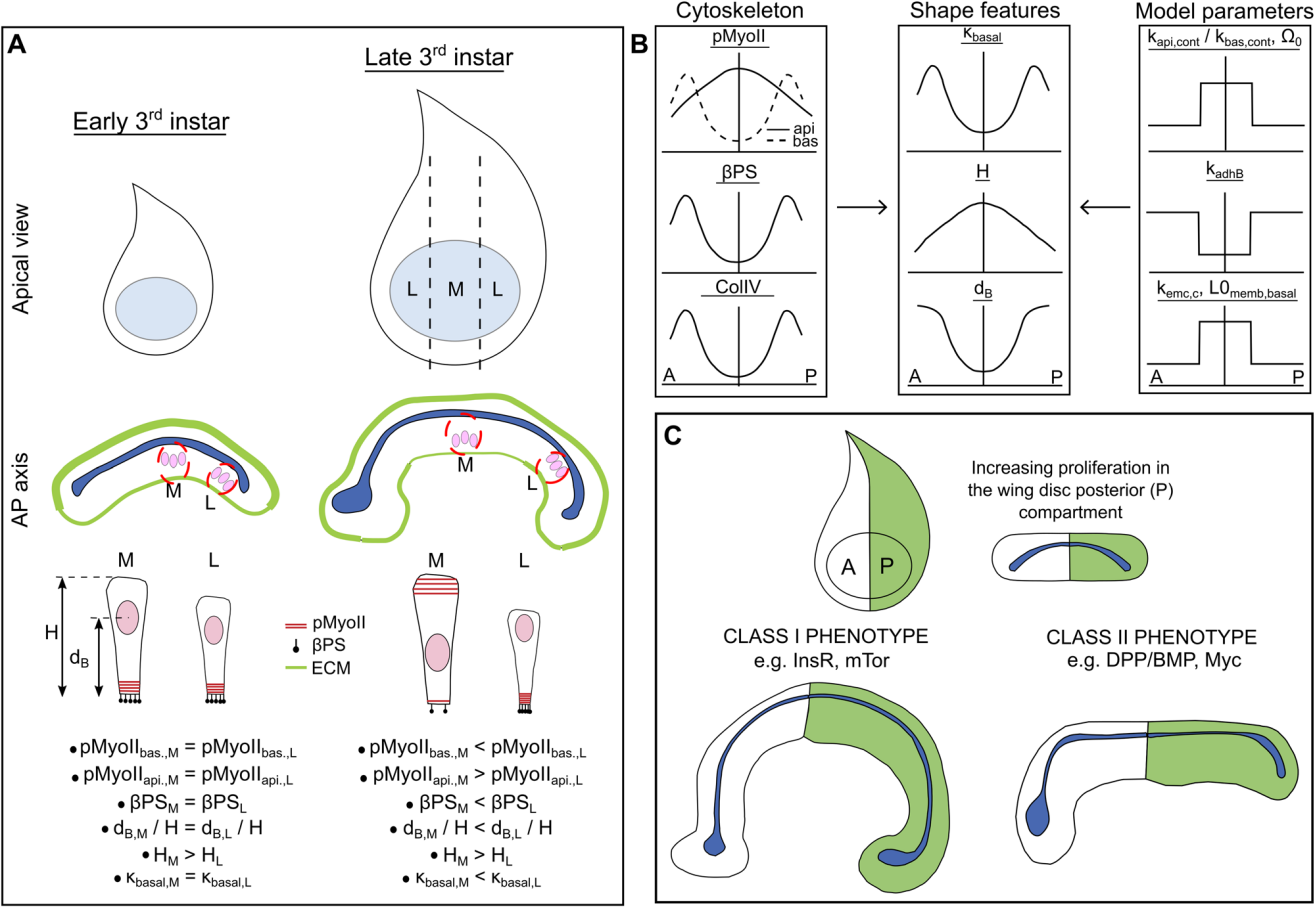
Summary of mechanistic insights into how perturbations to various growth signaling pathways impact tissue shape. **A** As the wing disc grows, the central, medial (M) domain of the pouch flattens and thickens while cells in the lateral (L) region increase their curvature. The cytoskeletal dynamics and morphological parameters are consistently adjusted with the age of the tissue. **B** Patterning of model parameters that produce shape features consistent with patterning of cytoskeletal components. **C** Increasing the proliferation rate through genetic perturbation in a compartment-specific manner resulted in distinct classes of morphological phenotypes.

Notably, we show that increasing proliferation through distinct mechanisms results in two very different wing disc phenotypes (Fig. 9). While increasing proliferation through the expression of *InsR*^*CA*^ causes an increase in basal curvature of the tissue, expressions of *Tkv*^*CA*^ and *Myc* flatten the tissue even with increased proliferation. Model simulations show that upregulation of either apical contractility or basal cell-ECM adhesion in the genetic perturbations can drive tissue flattening despite increasing proliferation. Thus, our study shows that a combination of spatial patterning of cytoskeletal regulators and proliferation shapes the organ locally during development to prepare the wing imaginal disc for later pre-pupal and pupal stages of development^46^.

Our experimental data and model simulations also highlight the existence and role of spatial heterogeneity in ECM stiffness (Fig. 3). The experimentally observed flattening of the pouch medial domain can be explained by patterning the local stiffness of ECM associated with the columnar pouch cells along the AP axis. This result extends our previous findings, where we showed that a difference of stiffness between the two ECM layers, either adhering to the peripodial squamous cells or columnar disc pouch cells, maintains the bent shape of wing imaginal disc^30^. Recently, Harmansa et al.^23^ further showed that a difference in the growth rate of the pouch cells and their corresponding ECM leads to the variation of stiffness of ECM across pouch cells and squamous cells^23^. However, in that model, the stiffness ECM associated with the pouch is assumed to be homogeneous throughout the entire tissue and does not explore the spatial patterning of ECM stiffness in the planar direction or near the lateral bends. Collectively, these studies suggest that future investigations are needed to understand the spatial patterning of ECM stiffness across all anatomical axes and to perform biophysical measurements.

Future developments of our multi-scale computational model will include a more detailed, microscale stochastic description of the interaction between the actin filaments and myosin motors such that the directionality of the contractile forces can be explicitly incorporated and the mitotic rounding process along with cell division be extended to include a complete representation of mechanistic details. Developing fully chemical-mechanical models based on the approach from Ramezani et al. will also enable a fully integrated perspective of organ size control^47^. The experimentally calibrated computational framework opens avenues for exploring feedback loops between tissue shape and cell proliferation. Moreover, it will facilitate exploring further the independent modulation of various parameters controlling the cell mechanical properties.

Our findings reflect similar mechanisms of morphogenesis in other developmental contexts. For example, the dynamic and autonomous morphogenetic process of formation of the optic cup, i.e., retinal primordium, is reminiscent of the tissue flattening that occurs before subsequent eversion of the wing imaginal disc^45^. During the formation of the mammalian optic cup, the invagination happens in four stages. Flattening of the distal region of the initially hemispherical vesicle is observed in the first two stages. The angle at the hinge then begins to become narrower, following which, in phase four, the neural retina epithelium starts to expand as an apically convex structure, forming a cup via progressive invagination. This morphogenetic process, specifically the last two phases, resembles the tissue bending and flattening in the wing disc. Balancing mutually antagonistic morphogenetic processes is also seen in mammalian development, including the developing mouse lens^48^. Overall, the principal findings identified in our work can be generalized and incorporated into our understanding of development across multiple organ systems in animals due to the underlying biochemical and biomechanical similarities that drive morphogenesis^14,49^.

## Methods

### Experimental and image analysis methods

#### Fly stocks and culture

*Drosophila* were raised in a 25 °C incubator with a twelve-hour light cycle unless specified otherwise. Virgins from Gal4 lines were collected twice a day from the bottles. Virgins were crossed with males carrying the indicated UAS line constructs in a ratio of females to males 15:5. The crosses were staged for 4 h to collect the correct aged larvae. The wild-type Oregon-R fly line is a long-standing stock in the Zartman lab acquired from the N. Yakoby lab. The following transgenic stocks were obtained from Bloomington Drosophila Stock Center (BDSC): UAS-RyR^RNAi^ (BDSC#31540), UAS-InsR^DN^(BDSC#8253), UAS-InsR^CA^(BDSC#8263), UAS-Tkv^RNAi^ (BDSC#31041), UAS-Tkv^CA^(BDSC#36537), Nubbin-Gal4 (BDSC#25754), Engrailed-Gal4 (BDSC#25752), UAS-Myc (BDSC#9674), UAS-mTOR^RNAi^ (BDSC#33951), UAS-Myc^RNAi^ (BDSC#25783), and UAS-mTOR^DN^ (BDSC#7013).

#### Genotypes by figure

Figures 1, 2, 3: *Oregon-R: D. melanogaster (Oregon-R)*

Figure 4: (B) *MS1096-Gal4* > *UAS-mys*^*RNAi*^, (C) *MS1096-Gal4* > *UAS-Ryr*^*RNAi*^, (C’) *MS1096-Gal4* > *UAS-Rho1*^*RNAi*^

*MS1096-Gal4: w[1118] P{w[+mW.hs]=GawB}Bx[MS1096]; P{w[+mC]* = *UAS-Dcr-2.D}2*

*UAS-mys* ^*RNAi*^ *: y[1] v[1]; P{y[+t7.7] v[+t1.8]=TRiP.HMS00043}attP2*

*UAS-RyR* ^*RNAi*^ *: y[1] v[1]; P{y[+t7.7] v[+t1.8]=TRiP.JF01100}attP2*

*UAS-Rho* ^*RNAi*^ *: y[1] v[1]; P{y[+t7.7] v[+t1.8]=TRiP.JF02809}attP2*

Figure 5: (D) *MS1096-Gal4* > *UAS-mys*^*RNAi*^

Figure 7: (A-i, B-i, B’-i) *Engrailed-Gal4*, (A-ii, B-ii, B’-ii, D, E) *Engrailed-Gal4* > *UAS-InsR*^*DN*^, (G) *Nubbin-Gal4*, (G’) *Nubbin-Gal4* > *UAS-InsR*^*CA*^, (I, J) *Engrailed -Gal4* > *UAS-Tkv*^*CA*^, (E, F-i) *Engrailed -Gal4* > *UAS-Myc*

*Engrailed-Gal4: P{w[+mC]* = *UAS-Dcr-2.D}1, w[1118]; P{w[+mW.hs] = en2.4-GAL4}e16E, P{w[+mC]* = *UAS-2xEGFP}AH2* > *UAS-mys*^*RNAi*^*: y[1] v[1]; P{y[+t7.7] v[+t1.8]=TRiP.HMS00043}attP2*

*Nubbin Gal4: P{w[+mC]* = *UAS-Dcr-2.D}1, w[1118]; P{w[+mW.hs] = GawB}nubbin-AC-62*

*UAS-InsR* ^*DN*^ *: y[1] w[1118]; P{w[+mC]=UAS-InR.K1409A}3*

*UAS-InsR* ^*CA*^ *: y[1] w[1118]; P{w[+mC]=UAS-InR.A1325D}2*

*UAS-Tkv* ^*CA*^ : w[*]; P{w[+mC]=UAS-tkv.CA}3

*UAS-Myc: w[1118]; P{w[+mC]=UAS-Myc.Z}132*

Supplementary Fig. 1, 2, 3, 4, 23, 24, 25: *Oregon-R*

Supplementary Fig. 10: (A) MS1096-Gal4>UAS-Ryr^RNAi^ (B) MS1096-Gal4>UAS-Mys^RNAi^, (C, D) MS109-Gal46>Rho^RNAi^, (E) Engrailed-Gal4>Mys^RNAi^

Supplementary Fig. 12: (A) MS1096-Gal4>UAS-Rho1^RNAi^

Supplementary Fig. 13: (A-i) Engrailed-Gal4, (A-ii) Engrailed-Gal4>InsR^DN^ (A-iii, B, C) *Engrailed-Gal4* > *UAS-InsR*^*CA*^, (D) *Apterous-Gal4* > *UAS-Myc*

Supplementary Fig. 14: (A-i, B-i) Apterous-Gal4>Ryr^RNAi^, (A-ii, B-ii, C, F) Apterous-Gal4>mTOR^DN^

*UAS-mTOR*^*DN*^*:* y[1] w[*]; P{w[+mC]=UAS-mTOR.TED}II

Supplementary Fig. 16: (A) Apterous-Gal4 x White, (A’) Apterous-Gal4>UAS-mTOR^RNAi^

Supplementary Fig. 17: (A) *Engrailed-Gal4*, (B) *Engrailed-Gal4* > *UAS-Tkv*^*RNAi*^,(C, D, E) *Engrailed-Gal4* > *UAS-Tkv*^*CA*^

*UAS-Tkv* ^*RNAi*^ *: y[1] v[1]; P{y[+t7.7] v[+t1.8]=TRiP.JF01486}attP2*

Supplementary Fig. 18: Engrailed-Gal4>Myc

Supplementary Fig. 20: (B) *MS1096-Gal4* > *UAS-mys*^*RNAi*^

Supplementary Fig. 21: Ani.RBD-EGFP

#### Immunohistochemistry

Wing imaginal discs were dissected as described in previous publications^22^. Details of methods and reagents are found in Supplementary Methods

#### Confocal Microscopy

The wing imaginal discs were imaged using a Nikon Eclipse Ti confocal microscope with a Yokogawa spinning disc and MicroPoint laser ablation system, and a Nikon A1R-MP laser scanning confocal microscope. For the two confocal microscopes, image data were collected on an IXonEM+colled CCD camera (Andor Technology, South Windsor, CT) using MetaMorph v7.7.9 software (Molecular Devices, Sunnyvale, CA) and NIS-Elements software, respectively. Discs were imaged throughout the entire depth of z-planes with a step size of 0.8-1 μm, depending on sample thickness, with a 40x and 60x oil objective with 200 ms exposure time, and 50 nW, 405 nm, 488 nm, 561 nm, and 640 nm laser exposure at 44% laser intensity. The imaging was performed from apical to basal surface so that peripodial cells were imaged first, followed by the columnar cells of the wing disc. Optical slices were taken at distances equaling half the compartment length. Tiling was performed on images to get the entire sample in the field of view, and QuickStich^50^ was utilized to stitch individual tiles.

#### Image analysis

For the Oregon-R staging data, CSBDeep^51^ was used on the Actin fluorescent channel for denoising (Figs. 1–5). For visualization purposes, the background subtraction of the pMyoII channel data was done using a rolling ball algorithm in ImageJ^52^. Intensity values for quantifications in this paper were measured using the raw data without any image corrections. Brightness and contrast adjustments were made to minimize saturation during the visualization of Rho1 data for InsR and Tkv genetic perturbations (Figs. 7 and 8). Analysis of curvature at the basal surface of a *Drosophila* wing imaginal disc cross-section was carried out using an in-house Python-based pipeline described in Supplementary subsection S4.1 (Supplementary Fig. 23). StarDist^53^, an open-source ImageJ plugin, was used to segment the nuclei. Details about quantifications of cell height and nuclear positioning can be found in the supplementary information of the text (Supplementary Figs. 24, 25).

#### Statistics and reproducibility

We performed a two-sided two sample t-test to compare the significance of differences between the means of any two groups of data. For each comparison, we have reported the following statistics: the t-statistic, p-value, mean difference, and the lower and upper bound of the 95% confidence interval for the mean difference^54^. For comparison of curvature profiles, a t-test was additionally followed by a Cohen’s d^55^ measure to account for the large number of points in averaged curvature profile. We report t-statistic, p-value and Cohen’s d measure for estimating the practical significance of comparison. All the statistical tests were carried out using Python’s Scipy^56^ module. Please see Supplementary subsection S8.1 for detailed statistics tables corresponding to specific figures within this manuscript.

### Description of the computational model

#### Computational modeling background

Various computational modeling approaches, ranging from continuum to discrete cell-based models^57^, have been introduced to study tissue development and growth^4^. In the context of studying the *Drosophila* wing imaginal disc, continuum models employing the finite element method have been recently used to study the bending of the wing disc^23^. On the other hand, discrete cell-based models, classified as lattice-based or off-lattice models, have been successfully utilized to investigate different aspects of tissue growth and development^16,58–62^. For example, the Cellular Potts (CPM) modeling method, a lattice-based approach, describes cells as clusters of lattice sites that change shape via energy-based Monte Carlo algorithms. This modeling approach has been used to study fundamental biological processes, including but not limited to morphogenesis, cell aggregation, cell sorting, and thrombus formation^63–67^. Off-lattice vertex-based models have been implemented to investigate tissue morphogenesis and the regulation of tissue size, among other biological applications^58,68–73^. Although vertex and CPM models have been successfully used to study various biological systems, it is often hard to calibrate representations of cellular and subcellular mechanical properties in these models using micro-scale experimental data^58,63,64^. In this paper, we use the general off-lattice Subcellular Element (SCE) modeling approach, initially developed by Newman et al.^74^ and based on the coarse-grained molecular dynamics method. It allows us to combine submodels at the micro-scale into a model by choosing appropriate levels of resolution, as well as represent mechanical properties and shapes of cellular membranes, nuclei, and the ECM in detail and calibrate the multi-scale model using experimental data at the subcellular, cellular and tissue scales. (For a more detailed review of different models, please see the Supplementary Information section S3.1 Overview of computational modeling approaches.)

#### Description of the SCE model

The general SCE modeling approach^22,30,31^ was used as the basis for the model presented here. Here, our SCE-type multi-scale model in detail represents cell deformation and proliferation and integrates mechanical properties, including cell membrane elasticity, cell pressure, polarized actomyosin contractility with nuclear movement, and cell-cell and cell-ECM adhesivity. Another key advantage of this SCE modeling approach is that most of the cell mechanical properties considered can be directly or indirectly calibrated based on experimental data^30,75^. This computational modeling framework is used to simulate a two-dimensional (2D) cross-sectional profile of the *Drosophila* wing imaginal disc along the anterior-posterior axis adjacent to the DV compartment boundary. Using a detailed 2D model allows for simulating large numbers of cells with high resolution and with particular attention to mechanical cell properties and small changes in tissue structure and shape. Given that the cross-section is composed of boundary cells, squamous cells, and columnar cells, the model includes a separate description for each cell type (Supplementary Fig. 22). Important distinctions from our previous model^22^ are detailed representations of cell division and actomyosin contractility in different parts of a cell. The model includes membrane nodes, nucleus nodes, and ECM nodes. Energy potentials are used to describe interactions between different nodes, as in Nematbakhsh and Levis et al.^22^.

The total potential energy in the general SCE model is calculated across node representations of different components of individual cells, and the position of each node of interest is determined via the following Langevin equations of motion:

1 $${C}_{{nuc}}\frac{d}{{dt}}{{{{{{\boldsymbol{x}}}}}}}_{{nuc}}=-(\nabla {E}_{{nuc}}+\nabla {E}_{v})+{\xi }_{s}$$

2 $${C}_{{memb}}\frac{d}{{dt}}{{{{{{\boldsymbol{x}}}}}}}_{{memb}}=	 -(\nabla {E}_{{memb}}+\nabla {E}_{{memb},{bend}}+\nabla {E}_{{api},{cont}}+\nabla {E}_{{bas},{cont}} \\ 	+\nabla {E}_{v}+\nabla {E}_{{vol}}+\nabla {E}_{{adhL}}+\nabla {E}_{{adhB}}+\nabla {E}_{{adhA}})+{\xi }_{s}$$

3 $${C}_{{ecm}}\frac{d}{{dt}}{{{{{{\boldsymbol{x}}}}}}}_{{ecm}}=-(\nabla {E}_{{ecm}}+\nabla {E}_{{ecm},{bend}}+\nabla {E}_{v}+\nabla {E}_{{adhB}})+{\xi }_{s}$$

where ${C}_{{nuc}}$, ${C}_{{memb}}$ and ${C}_{{ecm}}$ are damping coefficients and ${\xi }_{s}$ is the stochastic force satisfying the Fluctuation-Dissipation Theorem^76^. In this paper, we neglect the stochastic force term on the right-hand side of Eqs. (1)–(3) by setting ${\xi }_{s}=0$ for two important reasons. First, the cells in the wing disc cross-section are part of a tightly packed tissue where the level of fluctuation of membranes is negligible. The second reason is that we use the coarse-graining approximation. Instead of modeling at the molecular level, a single node in our model represents a portion of cell nuclei, membrane, or ECM. Therefore, ${\xi }_{s}$ is assumed to be zero as the mean behavior. Although for this specific application, we neglect the stochastic term, we plan to include the non-zero stochastic term and study its effect in our future extended model.

In the SCE model, a linear spring potential, ${E}_{{memb}} = {\frac{1}{2}k}_{{memb}}{\left({L}_{{memb}}-L{0}_{{memb}}\right)}^{2}$ where ${L}_{{memb}}$ and $L{0}_{{memb}}$ are the lengths between connected nodes and the resting length respectively, is used to describe mechanical properties of the cell membrane (Supplementary Fig. 22A-F). The E-cadherin mediated cell-to-cell adhesion between cells of the same type is captured by a linear spring potential ${E}_{{adhL}}={\frac{1}{2}k}_{{adhL}}{\left({L}_{{adhL}}-L{0}_{{adhL}}\right)}^{2}$ (Supplementary Fig. 22E). Similarly, the adhesion between squamous and columnar cell membrane nodes is defined by the energy potential ${E}_{{adhA}}={\frac{1}{2}k}_{{adhA}}{\left({L}_{{adhA}}-L{0}_{{adhA}}\right)}^{2}$ while the membrane-to-ECM adhesion mediated by Integrin is defined by ${E}_{{adhB}}={\frac{1}{2}k}_{{adhB}}{\left({L}_{{adhB}}-L{0}_{{adhB}}\right)}^{2}$ (Supplementary Fig. 22 D, F). To account for membrane bending resistance, a bending spring potential ${E}_{{memb},{bend}}={\frac{1}{2}k}_{{memb},{bend}}{({\theta }_{{memb}}-{\theta }_{0})}^{2}$ is defined where ${\theta }_{{memb}}$ is the angle between two connected linear springs, and *θ*_0_ is the resting angle between two connected linear springs^22,30^. The ECM (${E}_{{ecm}}$) is also modeled similarly via placing a spring between adjacent ECM nodes (Supplementary Fig. 22D-F, Supplementary Information S-3.4) to account for both stretching and bending resistances. Note that for the ECM, ${E}_{{ecm},{bend}}={k}_{{ecm},{bend}}(1-\cos ({\theta }_{{ecm}}-{\theta }_{0}))$ where ${\theta }_{{ecm}}$ and ${\theta }_{0}$ represent the current and resting angles between two connected ECM linear springs^22^. Volume-exclusion $({E}_{v})$ between different types of nodes is described by the Morse potential ${E}_{v}={U}_{v}\exp (\frac{-|{{{{{{\boldsymbol{x}}}}}}}_{i}-{{{{{{\boldsymbol{x}}}}}}}_{j}|}{{\xi }_{v}})-{W}_{v}\exp (\frac{-|{{{{{{\boldsymbol{x}}}}}}}_{i}-{{{{{{\boldsymbol{x}}}}}}}_{j}|}{{\gamma }_{v}})$ where ${{{{{{\boldsymbol{x}}}}}}}_{i}$ and ${{{{{{\boldsymbol{x}}}}}}}_{j}$ are vectors representing the positions of nodes $i$ and $j$ and ${U}_{v}$, ${\xi }_{v}$, ${W}_{v}$ and ${\gamma }_{v}$ are the Morse coefficients^22,30^.

Since we introduce apical actomyosin contractility in this paper, an additional linear spring energy potential term is introduced in Eq. (2) and denoted by ${E}_{{api} , {cont}}$. In this way, we capture actomyosin contractility in the apical (${E}_{{api},{cont}}$) and basal $({E}_{{bas},{cont}})$ parts of a columnar cell (Supplementary Fig. 22D). Note that ${E}_{{api},{cont}} = {\frac{1}{2}k}_{{api},{cont}}{(L-L{0}_{{api},{cont}})}^{2}$ and ${E}_{{bas},{cont}}={\frac{1}{2}k}_{{bas},{cont}}{(L-L{0}_{{bas},{cont}})}^{2}$ where $L$ represents the lengths between connected nodes while $L{0}_{{api},{cont}}$ and $L{0}_{{bas},{cont}}$ are the resting lengths of the apical and basal contractile springs, respectively. It is important to highlight several contractile springs in the apical and basal regions of columnar cells and that the number of springs is cell-dependent. For a detailed explanation of how the number of springs is determined, please see Supplementary Information section S-3.3. For completeness, we reproduced the table of energy potentials (Table 1) from our previous publication^22^, with the addition of the description of the new potential ${E}_{{api},{cont}}$, in order to provide a detailed description of the potential functions in the right-hand side of Eqs. (1)–(3).

**Table 1:** Energy potentials used in the SCE model

Potential function	Type	Biological representation
${E}_{{nuc}}$	Morse potential	Size of a cell’s nucleus
${E}_{v}$	Morse potential	Volume exclusion
${E}_{{memb}}$	Spring	Elasticity of the cell membrane
${E}_{{memb},{bend}}$	Bending Spring	Bending elasticity of the cell membrane
${E}_{{api},{cont}}$	Spring	Apical actomyosin contractility inside the cells and above the nucleus
${E}_{{bas},{cont}}$	Spring	Basal actomyosin contractility inside the cells and below the nucleus
${E}_{{vol}}$	Lagrange Multiplier	Volume conservation of the cytoplasm for each cell
${E}_{{adhL}}$	Spring	Cell-cell adhesion mediated by E-cadherin
${E}_{{adhB}}$	Spring	Cell-ECM adhesion mediated by Integrin
${E}_{{adhA}}$	Spring	Adhesion between columnar and squamous cells
${E}_{{ecm}}$	Spring	Extracellular matrix stiffness
${E}_{{ecm},{bend}}$	Bending Spring	Extracellular matrix bending stiffness

Given that it is valid to assume that cellular motion is under an overdamped regime for most biological systems^22,30,77,78^, we assume that the nodes in our wing model are also in an overdamped regime. In this way, the inertia force acting on each node is neglected. Moreover, Eqs. (1)–(3) include a damping coefficient (${C}_{{nuc}}$, ${C}_{{memb}}$, ${C}_{{ecm}}$) to account for the viscous drag acting on each node due to the surrounding environment. (A detailed description of the types of potential energies and model construction can be found in Table 1, Nematbakhsh and Levis et al.^22^ and Supplementary Information S-3.5). The parameter ranges of this computational model have been calibrated using a combination of experimental data from this study and from literature (Supplementary Table 1). Various parameter values were taken from our previous publications (Nematbakhsh and Levis et al.^22^, Nematbakhsh et al.^30^), where they were calibrated based on specific experimental results. Parameters such as spring constants, which are not biological, have been calibrated by comparison with specific experiments and experimental data. In addition, we used actin and pMyoII intensities quantified in this study to calibrate the number of springs and spring coefficients, respectively, associated with the apical and basal contractility. To ensure that the parameter values were in agreement with experimentally quantified data, we relied on three metrics for calibration: (1) local basal curvature, (2) tissue thickness, and (3) nuclear positioning (Fig. 1B). (A detailed description of our calibration pipeline can be found in the Supplementary Information section S-3.5 Model calibration pipeline).

#### Nonuniform apical and basal actomyosin contractility

A feature of the current model represents the nonuniform spatial patterning of actomyosin contractility in the apical and basal parts of a cell. Namely, interactions between actin and myosin lead to the formation of a contractile force that creates constriction in the apical and basal portions of the columnar cells. In our computational model, this constriction effect is represented by the contractile springs linking the lateral sides of a columnar cell. Note that the interaction between actin and myosin is mostly membrane-bound, and therefore, the contractile force is exerted on the membrane surface, altering the circumference of the cross-sections of each columnar cell.

While such contractile forces may manifest themselves in multiple directions, we only considered the apical and basal contractile forces that are perpendicular to the apical-basal direction, which constrict the lateral sides of the cells, as the dominant factor giving rise to the elongated rectangular geometry of the columnar cells. Therefore, in a 2D setting, this circumferential contractile force is projected on the 2D plane where our cross-sectional model tissue resides in the form of linear springs. Thus, there is no direct interaction between the nuclei and the contractile springs, although some model simulations may falsely suggest otherwise. The contractile force exerted by the actomyosin network on the cell membrane is therefore described by the linear spring potential, ${E}_{*,{cont}}={\frac{1}{2}k}_{*,{cont}}{\left(\left|x\right|-{x}_{0}\right)}^{2}$, where $x$ is the distance between a pair of membrane nodes connected by the contractile spring. A detailed description of the application for the nonuniform actomyosin patterning can be found in the following section and the last section.

#### Spatial representation of the model of the imaginal wing disc

The cross-sectional view of a wing disc can be separated into approximately left, middle, and right regions. The middle section is often found to be relatively flat in comparison to the left and right sections. At the same time, significant bending is observed at the junctions between the three sections (Fig. 2). Based on this observation, we first simplified our assumption of the nonuniform basal and apical contractility patterns as a step function. For details of the computational model related to membrane stiffness and membrane-to-membrane adhesion, please see^22^. To systematically study the effect of perturbing both apical and basal actomyosin contractility, we divided the columnar cells of the model tissue into three uniformly spaced sections, for example, the apical contractility profile is set to be $(a,1.0,a)$ and the basal contractility profile is set as $(1.0,a,1.0)$ where $0\le a\le 1.0$ (Fig. 3E-i, Supplementary Fig. 6, 7). These manually chosen nonuniform patterns of actomyosin contractility are utilized to perform model validation. Such validation is necessary to confirm that the resulting physical behavior of the model is plausible and helps narrow down the range of possible perturbations to evaluate. These simulation results are in good agreement with experimentally observed tissue shapes (Fig. 3).

#### Cell growth and mitotic rounding

Several key features were developed to study the effect of cell growth and division on the shape generation of the wing disc. Such features include cell growth via the increase of cell volume, mitotic rounding events, cell division, and an increase of the ECM nodes within the model. The volume conservation (or constraint) in the existing model utilizes a Lagrange multiplier energy function, ${E}_{{vol}}={k}_{{vol}}{(\varOmega -{\varOmega }_{0})}^{2}$, where ${k}_{{vol}}$, $\varOmega$, ${\varOmega }_{0}$ are the strength of the enforcement of volume constraint, current cell volume, and target (equilibrium) cell volume, respectively. To represent the increase in cell volume during cell growth, the equilibrium cell volume is increased linearly by a fixed value per simulation time step, namely ${{\varOmega }_{0}}^{{new}}={{\varOmega }_{0}}^{{init}}+\epsilon T$. Here $\epsilon < < 1$ is a small positive real number representing the increment in volume per simulation time step, $T$ is the current total simulation time, and ${{\varOmega }_{0}}^{{init}}$ is the equilibrium cell volume at the starting time point of cell growth. Furthermore, the maximal value ${{\varOmega }_{0}}^{\max }$ for ${{\varOmega }_{0}}^{{new}}$ is set based on the expected cell volume before cell division occurs. Note that ${{\varOmega }_{0}}^{\max } < 2{{\varOmega }_{0}}^{{init}}$ since the cross-section of the cell does not double even if the 3D volume is doubled. New ECM nodes are added locally when a certain portion of the ECM exceeds a fixed tension threshold, which arises due to individual cellular growth and division. This growth mismatch ensures that the cellular growth in the columnar epithelial layer is always greater than the ECM growth because the addition of ECM nodes depends upon the ECM’s stretching due to the addition of new daughter cells from cell division. This model assumption is equivalent to growth in the columnar cell layer, which is upstream (and faster) than the ECM, following the mechanism described in Harmansa et al.^23^.

#### Modeling the basal constriction effect and the dynamics of actomyosin patterning during mitotic rounding

Mitotic rounding in the *Drosophila* wing disc is special because the columnar cells experience an increased basal constriction that eventually pushes the majority of the cell interior content apically. This leads to an enlarged sphere at the apical side whose diameter is roughly a four-fold increase compared to the non-growing cell^30^. During this process, it was experimentally observed that the enrichment of actin and myosin concentration occurred near the basal section of the cell. This increased constriction effect is modeled as a time-dependent gradual increase in the number of actomyosin contractile springs in the existing model (Fig. 3E-i) using the equation ${{H}_{0}}^{{new}}={{H}_{0}}^{{init}}+{hT}$. Here ${{H}_{0}}^{{init}}$ is the value at the basal point calculated as the average of the membrane node coordinates in the basal portion of the cell, $h < < 1$ is a positive real number representing the increment per simulation time step in actomyosin network buildup, and $T$ is the current total simulation time. $h$ is approximated by using the experimentally observed mitotic rounding time duration and the portion of a mitotic rounding cell with high intensity of actomyosin presence at the onset of cell division (Fig. 1D, E). In this way, a contractile spring is active when the distances from the membrane nodes connected by such contractile spring to the basal point (${H}_{{cont}}$) satisfy ${H}_{{cont}} < {{H}_{0}}^{{new}}$. Therefore, as ${{H}_{0}}^{{new}}$ increases, the number of contractile springs also increases. The strength of the contractile springs is also increased to increase the constriction effect further. This combined effect would correspond to the increased actin and myosin concentration observed during the mitotic rounding process. Finally, at the end of the mitotic rounding process, we restore the value of the contractile spring constant to the initially prescribed value and assume that the actin concentration undergoes a restoration from a depolymerized state back to a pre-mitotic state.

#### Modeling cell division process

Cell division is modeled by constructing a division plane based on the centerline of each cell. Since the cell geometry can become rather distorted when simulating the mitotic rounding process, the division plane has to be carefully crafted. This was achieved by calculating the midpoints of the cell. These midpoints are based on the corresponding pair of nodes positioned at the two lateral sides of a given model cell. Given such a centerline, new cell membranes in the model can be constructed by shifting the centerline slightly toward the two lateral sides, leading to two separate cells. This algorithm also corresponds to the scenario in that the planar orientation of the mitotic spindle is established as observed in the wild-type tissue, leading to the division along the lateral direction.

Each cell that can undergo cell growth and division was assigned an initial growth progress (${GP}$) value ${{GP}}_{0} < 1$. Such value was increased by a fixed value $\delta < < 1$ per simulation time step. A cell in the model is considered to enter the mitotic rounding process when $({{GP}}_{0}+\delta T) > {{GP}}_{{mit}}$, where $T$ is the current total simulation time, and ${{GP}}_{{mit}}$ satisfying ${{GP}}_{0} < {{GP}}_{{mit}} < 1$ is a threshold value. When $({{GP}}_{0}+\delta T)\ge 1$, the cell is considered to reach the end of the M phase of the cell cycle leading to subsequence cell division. To ensure that premature cell division with respect to cell volume increase does not occur, the small increments used in the cell volume and actomyosin contractile spring are calibrated so that the cell growth and division process can be completed in a timely manner. After a division event completes, the resulting cells are assigned a new ${GP}$ value to indicate how fast such cells will undergo the mitotic rounding and cell division process again.

To ensure numerical stability in our simulations, the advancement in the cell cycle of a given cell is suspended if its immediate neighboring cell is already undergoing mitotic rounding and cell division. In terms of biological relevance, this is equivalent to the assumption that cells under high external stress are prevented from entering the mitotic phase. Lastly, in the simulation results presented in this paper, each cell can perform a maximal number of 10 cell divisions.

#### Modeling the introduction of a new cell in the cross-section

At the end of cell division, a random number is generated to determine whether a new cell is introduced into the same plane (or cross-section). Since cell division can produce a new cell that would lie within the same cross-section or outside of the cross-section, it is necessary to consider both in-plane and out-of-plane cell division. The probability of this event can be experimentally approximated by calculating the frequency of new cells occurring in the same cross-section post-division. If a new cell is not introduced in the same cross-section post-cell division, the equilibrium volume is restored to the original value ${{\varOmega }_{0}}^{{init}}$ by a method discussed in an earlier section, except we use expression ${{\varOmega }_{0}}^{{new}}={{\varOmega }_{0}}^{{postdiv}}-\epsilon T$ where ${{\varOmega }_{0}}^{{new}}\ge {{\varOmega }_{0}}^{{init}}$.

Due to the unique mitotic rounding process, special care was taken to ensure numerical stability (Fig. 1D, E). Since columnar cells in the model are tightly packed and experience an increase in cell volume during mitotic rounding, volume exclusion is manipulated to prevent membranes from different cells from overlapping. This is done via a temporary but significant increase in the magnitude of the volume exclusion energy potential coefficient which becomes active as a cell enters the mitotic process and briefly post cell division.

As described earlier, the number of contractile springs corresponds to the presence of actin filaments found throughout the wing disc. In this model, we use the actin intensity (or concentration) observed in experiments to determine the number of contractile springs per cell in the model. An additional simplification was applied by calculating the average value of the recorded intensity according to its corresponding spatial position on the wing disc (Fig. 1). However, this simplification can be discarded if the actomyosin intensity is governed by a time-dependent chemical signaling mechanism, and individual intensity can be prescribed to each cell.

#### SCE model of nuclear and cell shape dynamics during organ growth

How morphogenesis arises from the interplay between mechanical contraction and signaling networks remains unclear. To fill this knowledge gap, we specifically explore the relative contributions of proliferation and actomyosin contractility in controlling curvature, height, and nuclear positioning. During actomyosin contraction, pMyoII is responsible for bringing actin filaments closer to each other, generating the contractile force. Such a force also depends on the availability of actin filaments^79^. The model incorporates actin density by utilizing a certain number of (linear) contractile springs in different parts of each cell. The impact of myosin on actin is represented by the spring constant of a contractile spring. In this paper, we consider both apical and basal contractility.

#### Determination of the number of springs in non-mitotic cells

The number of apical and basal actomyosin contractile springs within a non-mitotic cell depends on the height of the cell (${H}_{{cell}}$) and the local actin intensities. Supplementary Information section S-3.3 further details the determination of the number of contractile springs.

### Reporting summary

Further information on research design is available in the Nature Portfolio Reporting Summary linked to this article.

### Supplementary information

Supplementary Information

Description of Additional Supplementary Files

Supplementary Movie 1

Supplementary Movie 2

Supplementary Movie 3

Supplementary Movie 4

Supplementary Movie 5

Supplementary Movie 6

Supplementary Movie 7

Supplementary Movie 8

Supplementary Movie 9

Supplementary Movie 10

Reporting Summary

### Source data

Source Data

## Data Availability

Due to data size constraints, the 3D confocal microscopy data are available on request from the corresponding authors. This includes all of the representative source data and analysis files for experiments within this manuscript. Source data are provided with this paper.

## Code availability

An open-source C + + and CUDA implementation of the computational model is available on GitHub (https://github.com/jenniferrangel/Episcale_CrossSectionalView.git) or in Zenodo (https://zenodo.org/badge/latestdoi/468540858)^80^. Source data are provided with this paper.

## References

[1] Urdy, S., Goudemand, N. & Pantalacci, S. Chapter Seven - Looking Beyond the Genes: The Interplay Between Signaling Pathways and Mechanics in the Shaping and Diversification of Epithelial Tissues. in *Current Topics in Developmental Biology* (ed. Orgogozo, V.) vol. **119** 227–290 (Academic Press, 2016). 10.1016/bs.ctdb.2016.03.005 27282028

[2] Iskratsch T, Wolfenson H, Sheetz MP (2014). Appreciating force and shape—the rise of mechanotransduction in cell biology. Nat. Rev. Mol. Cell Biol..

[3] Gorfinkiel N, Martinez Arias A (2021). The cell in the age of the genomic revolution: Cell Regulatory Networks. Cells Dev..

[4] Velagala, V., Chen, W., Alber, M. & Zartman, J. J. Chapter 4.1 - Multiscale models coupling chemical signaling and mechanical properties for studying tissue growth. In *Mechanobiology* (ed. Niebur, G. L.) 173–195 (Elsevier, 2020) 10.1016/B978-0-12-817931-4.00010-8.

[5] Narciso C, Zartman J (2018). Reverse-engineering organogenesis through feedback loops between model systems. Curr. Opin. Biotechnol..

[6] Wu Q, Kumar N, Velagala V, Zartman JJ (2019). Tools to reverse-engineer multicellular systems: case studies using the fruit fly. J. Biol. Eng..

[7] Villaverde AF, Banga JR (2014). Reverse engineering and identification in systems biology: strategies, perspectives and challenges. J. R. Soc. Interface.

[8] Csete ME, Doyle JC (2002). Reverse Engineering of Biological Complexity. Science.

[9] Widmann TJ, Dahmann C (2009). Dpp signaling promotes the cuboidal-to-columnar shape transition of Drosophila wing disc epithelia by regulating Rho1. J. Cell Sci..

[10] Kirkland NJ (2020). Tissue mechanics regulate mitotic nuclear dynamics during epithelial development. Curr. Biol..

[11] Meyer EJ, Ikmi A, Gibson MC (2011). Interkinetic nuclear migration is a broadly conserved feature of cell division in pseudostratified epithelia. Curr. Biol. CB.

[12] Neto-Silva RM, Wells BS, Johnston LA (2009). Mechanisms of growth and homeostasis in the Drosophila wing. Annu. Rev. Cell Dev. Biol..

[13] Hannezo E, Prost J, Joanny J-F (2014). Theory of epithelial sheet morphology in three dimensions. Proc. Natl Acad. Sci..

[14] Zartman JJ, Shvartsman SY (2010). Unit operations of tissue development: epithelial folding. Annu. Rev. Chem. Biomol. Eng..

[15] Walpole J, Papin JA, Peirce SM (2013). Multiscale computational models of complex biological systems. Annu. Rev. Biomed. Eng..

[16] Brodland GW (2015). How computational models can help unlock biological systems. Semin. Cell Dev. Biol..

[17] Tripathi BK, Irvine KD (2022). The wing imaginal disc. Genetics.

[18] Domínguez-Giménez P, Brown NH, Martín-Bermudo MD (2007). Integrin-ECM interactions regulate the changes in cell shape driving the morphogenesis of the Drosophila wing epithelium. J. Cell Sci..

[19] Pastor-Pareja JC, Xu T (2011). Shaping cells and organs in drosophila by opposing roles of fat body-secreted Collagen IV and Perlecan. Dev. Cell.

[20] Nelson CM (2009). Geometric control of tissue morphogenesis. Biochim. Biophys. Acta.

[21] Tozluoǧlu M (2019). Planar differential growth rates initiate precise fold positions in complex epithelia. Dev. Cell.

[22] Nematbakhsh A (2020). Epithelial organ shape is generated by patterned actomyosin contractility and maintained by the extracellular matrix. PLoS Comput Biol..

[23] Harmansa S, Erlich A, Eloy C, Zurlo G, Lecuit T (2023). Growth anisotropy of the extracellular matrix shapes a developing organ. Nat. Commun..

[24] Etournay R (2015). Interplay of cell dynamics and epithelial tension during morphogenesis of the Drosophila pupal wing. eLife.

[25] Guirao B (2015). Unified quantitative characterization of epithelial tissue development. eLife.

[26] Diaz de la Loza MC, Thompson BJ (2017). Forces shaping the Drosophila wing. Mech. Dev..

[27] Gou J, Stotsky JA, Othmer HG (2020). Growth control in the Drosophila wing disk. WIREs Syst. Biol. Med..

[28] Capdevila J, Guerrero I (1994). Targeted expression of the signaling molecule decapentaplegic induces pattern duplications and growth alterations in Drosophila wings. EMBO J..

[29] Ray RP, Nakata T, Henningsson P, Bomphrey RJ (2016). Enhanced flight performance by genetic manipulation of wing shape in Drosophila. Nat. Commun..

[30] Nematbakhsh A (2017). Multi-scale computational study of the mechanical regulation of cell mitotic rounding in epithelia. PLOS Comput. Biol..

[31] Sweet CR (2011). Modelling platelet–blood flow interaction using the subcellular element Langevin method. J. R. Soc. Interface.

[32] Wartlick O (2011). Dynamics of Dpp Signaling and Proliferation Control. Science.

[33] Hamaratoglu F, Lachapelle AM, de, Pyrowolakis G, Bergmann S, Affolter M (2011). Dpp Signaling Activity Requires Pentagone to Scale with Tissue Size in the Growing Drosophila Wing Imaginal Disc. PLOS Biol..

[34] Chugh M, Munjal A, Megason SG (2022). Hydrostatic pressure as a driver of cell and tissue morphogenesis. Semin. Cell Dev. Biol..

[35] Straus DS (1981). Effects of insulin on cellular growth and proliferation. Life Sci..

[36] Hopkins BD, Goncalves MD, Cantley LC (2020). Insulin–PI3K signalling: an evolutionarily insulated metabolic driver of cancer. Nat. Rev. Endocrinol..

[37] Orlova I, Silver L, Gallo G (2007). Regulation of actomyosin contractility by PI3K in sensory axons. Dev. Neurobiol..

[38] Zhang H, Stallock JP, Ng JC, Reinhard C, Neufeld TP (2000). Regulation of cellular growth by the Drosophila target of rapamycin dTOR. Genes Dev..

[39] Ducuing A, Keeley C, Mollereau B, Vincent S (2015). A DPP-mediated feed-forward loop canalizes morphogenesis during Drosophila. Dors. Clos. J. Cell Biol..

[40] Johnston LA, Prober DA, Edgar BA, Eisenman RN, Gallant P (1999). Drosophila myc Regulates Cellular Growth during Development. Cell.

[41] Nellen D, Burke R, Struhl G, Basler K (1996). Direct and Long-Range Action of a DPP Morphogen Gradient. Cell.

[42] Sauzeau V, Berenjeno IM, Citterio C, Bustelo XR (2010). A transcriptional cross-talk between RhoA and c-Myc Inhibts the RhoA/Rock-dependent cytoskeleton. Oncogene.

[43] Boudjadi S, Carrier JC, Groulx J-F, Beaulieu J-F (2016). Integrin α1β1 expression is controlled by c-MYC in colorectal cancer cells. Oncogene.

[44] Seher TC, Leptin M (2000). Tribbles, a cell-cycle brake that coordinates proliferation and morphogenesis during Drosophila gastrulation. Curr. Biol..

[45] Eiraku M (2011). Self-organizing optic-cup morphogenesis in three-dimensional culture. Nature.

[46] Aldaz S, Escudero LM, Freeman M (2013). Dual role of myosin II during Drosophila imaginal disc metamorphosis. Nat. Commun..

[47] Ramezani A (2023). A multiscale chemical-mechanical model predicts impact of morphogen spreading on tissue growth. Npj Syst. Biol. Appl..

[48] Chauhan BK, Lou M, Zheng Y, Lang RA (2011). Balanced Rac1 and RhoA activities regulate cell shape and drive invagination morphogenesis in epithelia. Proc. Natl Acad. Sci..

[49] Aydin O (2022). Principles for the design of multicellular engineered living systems. APL Bioeng..

[50] Brodskiy, P. A. et al. QuickStitch for seamless stitching of confocal mosaics through high-pass filtering and recursive normalization. *bioRxiv* 075440 10.1101/075440 (2016).

[51] Weigert M (2018). Content-aware image restoration: pushing the limits of fluorescence microscopy. Nat. Methods.

[52] Schneider CA, Rasband WS, Eliceiri KW (2012). NIH Image to ImageJ: 25 years of image analysis. Nat. Methods.

[53] Schmidt U, Weigert M, Broaddus C, Myers G (2018). Cell Detection Star.-convex Polyg..

[54] Snecdecor, G. & Cochran, W. *Statistical Methods*, *8th Edition | Wiley*. (Iowa State University Press, Ames, 1989).

[55] Cohen, J. *Statistical Power Analysis for the Behavioral Sciences*. (Academic Press, 2013).

[56] Virtanen P (2020). SciPy 1.0: fundamental algorithms for scientific computing in Python. Nat. Methods.

[57] Honda, H. & Nagai, T. *Mathematical Models of Cell-Based Morphogenesis: Passive and Active Remodeling*. (Springer Nature, Singapore, 2022) 10.1007/978-981-19-2916-8.

[58] Fletcher AG, Osterfield M, Baker RE, Shvartsman SY (2014). Vertex Models of Epithelial Morphogenesis. Biophys. J..

[59] Osborne JM, Fletcher AG, Pitt-Francis JM, Maini PK, Gavaghan DJ (2017). Comparing individual-based approaches to modelling the self-organization of multicellular tissues. PLOS Comput. Biol..

[60] Buchmann A, Alber M, Zartman JJ (2014). Sizing it up: the mechanical feedback hypothesis of organ growth regulation. Semin. Cell Dev. Biol..

[61] Mirams GR (2013). Chaste: An Open Source C++ Library for Computational Physiology and Biology. PLOS Comput. Biol..

[62] Camley BA, Rappel W-J (2017). Physical models of collective cell motility: from cell to tissue. J. Phys. Appl. Phys..

[63] Chen N, Glazier JA, Izaguirre JA, Alber MS (2007). A parallel implementation of the Cellular Potts Model for simulation of cell-based morphogenesis. Comput. Phys. Commun..

[64] Chaturvedi R (2005). On multiscale approaches to three-dimensional modelling of morphogenesis. J. R. Soc. Interface.

[65] Glazier JA, Graner F (1993). Simulation of the differential adhesion driven rearrangement of biological cells. Phys. Rev. E Stat. Phys. Plasmas Fluids Relat. Interdiscip. Top..

[66] Izaguirre JA (2004). CompuCell, a multi-model framework for simulation of morphogenesis. Bioinforma. Oxf. Engl..

[67] Xu Z, Chen N, Kamocka MM, Rosen ED, Alber M (2008). A multiscale model of thrombus development. J. R. Soc. Interface.

[68] Okuda S, Inoue Y, Adachi T (2015). Three-dimensional vertex model for simulating multicellular morphogenesis. Biophys. Physicobiology.

[69] Osterfield M, Du X, Schüpbach T, Wieschaus E, Shvartsman SY (2013). Three-dimensional epithelial morphogenesis in the developing Drosophila egg. Dev. Cell.

[70] Farhadifar R, Röper J-C, Aigouy B, Eaton S, Jülicher F (2007). The influence of cell mechanics, cell-cell interactions, and proliferation on epithelial packing. Curr. Biol. CB.

[71] Aegerter-Wilmsen T (2012). Integrating force-sensing and signaling pathways in a model for the regulation of wing imaginal disc size. Dev. Camb. Engl..

[72] Yu JC, Fernandez-Gonzalez R (2017). Quantitative modelling of epithelial morphogenesis: integrating cell mechanics and molecular dynamics. Semin. Cell Dev. Biol..

[73] Sussman DM (2017). cellGPU: massively parallel simulations of dynamic vertex models. Comput. Phys. Commun..

[74] Newman TJ (2005). Modeling multicellular systems using subcellular elements. Math. Biosci. Eng. MBE.

[75] Sandersius SA, Chuai M, Weijer CJ, Newman TJ (2011). Correlating Cell Behavior with Tissue Topology in Embryonic Epithelia. PLOS ONE.

[76] Kubo R (1966). The fluctuation-dissipation theorem. Rep. Prog. Phys..

[77] Newman TJ, Grima R (2004). Many-body theory of chemotactic cell-cell interactions. Phys. Rev. E Stat. Nonlin. Soft Matter Phys..

[78] Sandersius SA, Weijer CJ, Newman TJ (2011). Emergent cell and tissue dynamics from subcellular modeling of active biomechanical processes. Phys. Biol..

[79] Murrell M, Oakes PW, Lenz M, Gardel ML (2015). Forcing cells into shape: the mechanics of actomyosin contractility. Nat. Rev. Mol. Cell Biol..

[80] Tsai, K. & Rangel Ambriz, J. jenniferrangel/Episcale_CrossSectionalView: Episcale_CrossSectionalView. Zenodo 10.5281/zenodo.8045712 (2023).

