# Supplementary material for: Balancing competing effects of tissue growth and cytoskeletal regulation during Drosophila wing disc development
Source: Nat Commun. 2024 Mar 20;15:2477. doi: 10.1038/s41467-024-46698-7 (PMC10954670; doi:10.1038/s41467-024-46698-7)
Supplement: Supplementary file 1 — Supplementary Information [file 41467_2024_46698_MOESM1_ESM.pdf]

Supplementary Information:
Balancing competing effects of tissue growth and cytoskeletal regulation during *Drosophila* wing disc development

Nilay Kumar[1,#], Jennifer Rangel Ambriz[2,3,#], Kevin Tsai[2,3,#], Mayesha Sahir Mim[1], Marycruz Flores Flores[1], Weitao Chen[2,3], Jeremiah J. Zartman[1,4,*], Mark Alber[2,3,*]

[1]Department of Chemical and Biomolecular Engineering, University of Notre Dame, Notre Dame, Indiana, United States of America,
[2]Department of Mathematics, University of California, Riverside, California, United States of America,
[3]Interdisciplinary Center for Quantitative Modeling in Biology, University of California, Riverside, United States of America,
[4]Department of Biological Sciences, University of Notre Dame, Notre Dame, Indiana, United States of America

[#]These authors contributed equally to this work.
*Authors for correspondence: malber@ucr.edu (MA), jzartman@nd.edu (JJZ)

Content

S1. Supplemental details of methods and image analysis
S2. Supplementary results and figures
  - S2.1. Integrin (βPS) and Collagen IV (ColIV) co-localize with Actin within the cortical region of a dividing cell.
  - S2.2. The wing imaginal disc is flat across the AP axis at earlier stages of development (<72 h AEL).
  - S2.3. Correlation between $pMyoII_{apical}/pMyoII_{basal}$ and local cell height (H) increases with age of the disc. The gradient of H across the DV axis increases with the age of the disc.
  - S2.4. Colocalization of Actin, pMyoII and βPS increases with the age of the disc.
  - S2.5. Proliferation decreases with an increase in pouch size.
  - S2.6. In silico model scenarios of patterned cytoskeletal regulation across the AP axis.
  - S2.7. Tissue local height is regulated by the difference in apical-basal contractility.
  - S2.8. Knockdown of Integrin increases basal curvature, while knockdown of Rho1 reduces basal curvature.
  - S2.9. A cell-specific increase in control volumes (Ω_0) causes an increase in cell height (H) without changing its gradient along the DV axis.
  - S2.10. Loss of Rho1 pushes the nuclei basally.
  - S2.11. Compartment-specific expression of InsR and Myc to increase proliferation results in two distinct phenotypes.

S2.12. Inhibition of *mTOR* reduces cell proliferation and inhibits actomyosin contractility resulting in a decrease in cell height and basal curvature.
S2.13. Inhibition of *mTOR* does not affect the tissue geometry during the initial stages of development.
S2.14. Inhibiting Dpp signaling activity decreases Rho1 expression and reduces inwards bending at the pouch lateral domains.
S2.15. Overexpression of *Myc* causes a reduction in pMyoII and Rho1 fluorescence peaks along the AP axis.
S2.16. Increasing proliferation beyond the biological limits causes severe morphological changes in the shape of the simulated wing imaginal disc.
S2.17. Loss of cell-ECM adhesion causes apical constriction within the tissue.
S2.18. Expression levels of Rho1 quantified using Rho1 Biosensor ANI.RBD-EGFP and Rho1 antibody correlate.

S3. Computational modeling methods
S4. Image analysis and data quantification pipelines
S5. Experimental methods
S6. Supplementary videos
S7. Tables
S8. Statistical tests
S9. SI References (referring to citations in the SI text)

S1. Supplemental details of methods and image analysis

Additional method details for Fig. 2. *Drosophila* wing imaginal discs belonging to varying stages of larval development were dissected, fixed, and stained with Phalloidin and DAPI to mark Actin and nuclei, respectively (Fig. 2A). Principally, cross-sections along the AP axis were analyzed (Fig. 2B). An in-house pipeline for quantifying local basal curvature (κ_{basal}) (Supplementary text S-4.1) and tissue height (H) (Supplementary text S-4.2) was used to measure shape changes as the disc ages. For each stage, κ_{basal} was quantified for samples whose number has been indicated as an inset within the plot (Fig. 2C, top panel). The average κ_{basal} is plotted against the distance of the point of calculation from the center of the disc (ℓ). This is further normalized using half of the length of the basal surface (L_{AP}) along the AP axis. The standard deviation in prediction of κ_{basal} is shown as the shaded region within the plots. A similar analysis for H was performed, as shown in Fig. 2C (bottom panel).

Drosophila wing imaginal discs belonging to varying stages of larval development were dissected, and antibody staining of pMyoII and βPS, respectively, were carried out to measure the spatiotemporal dynamics of cytoskeletal regulation within the developing epithelia (Fig. 2D, D’). First, we analyzed how the ratio of apical to basal levels of pMyoII ($pMyoII_{apical}$ / $pMyoII_{basal}$) varies across the pouch medial and lateral domains (Fig. 2E). The pouch was discretized into 90 regional cells as described in Supplementary section S-4.2. The middle 30 cells constitute the medial domain, while the remaining 60 cells on the anterior/posterior ends constitute the lateral ends of

the pouch. Then, the difference between the average of $pMyoII_{apical}$ / $pMyoII_{basal}$ across the pouch medial (M) and lateral (L) domains was computed and normalized by the average value of $pMyoII_{apical}$ / $pMyoII_{basal}$ in the pouch lateral (L) domain. This quantity was calculated for multiple discs (sample sizes indicated in the inset of the plot) belonging to different stages of development (72-96 h AEL). Next, a box plot was used to visualize the variation of this ratio ($(X_M - X_L)/X_L$ where X represents $pMyoII_{apical}$ / $pMyoII_{basal}$) across different stages of wing disc development (Fig. 2E). Since βPS is primarily localized in the pouch basal surface, an in-house pipeline (Supplementary text S-4.2) was used to quantify the expression of βPS across the basal surface of the AP axis. The data was then exported to MATLAB[1] where the AP axis was split into three equal parts and at each of these sections, the averaged βPS fluorescence was evaluated. Next, we used these averaged values to compute the difference of βPS in the medial to lateral domain of the pouch (βPS_{medial} / $\beta PS_{lateral}$). The difference is further normalized by $\beta PS_{lateral}$. The ratio is next plotted for multiple samples belonging to different stages of development as a box plot (Fig. 2E').

Using the same pipeline (Supplementary subsection S-4.2), we calculated the fluorescence intensity of Actin, pMyoII and βPS for discs stained with the aforementioned cytoskeletal regulators. We plotted the values of Actin, pMyoII and βPS for multiple samples belonging to 72 and 96 h AEL larval stages of development (Fig. 2F, F'). A straight line was fit, and the R^2 value was calculated to measure the correlation strength. We first fit straight lines (color-coded) to the correlations for individual samples. We also fit a line to the aggregated data to measure the global correlation.

We also used the image analysis pipeline (Supplementary text S-4.2) on discs belonging to different stages of development for quantifying correlations between the ratio of $pMyoII_{apical}$ to $pMyoII_{basal}$ and local tissue height (*H*). A 2D scatter plot was first plotted with the x-axis and y-axis representing $pMyoII_{apical}$ / $pMyoII_{basal}$ and H, respectively (Fig. 2G, G'). Data extracted from each sample was plotted using unique colors. First, linear regression models were fitted individually to points extracted from each sample using scikit-learn. A solid color-coded line represents the predictions made by the model. Next, a single linear model was fit to the aggregated data from different samples represented by a solid black colored line. Averaged R^2 values for sample-wise model fits, along with the R^2 value of the global model fit, have been reported. A p-value for an F-test was calculated to evaluate the statistical significance of the global fit. Plotting and statistical tests were carried out in Python.

Lastly, we used our in-house codes (Supplementary subsection S-4.1, S-4.2) to calculate the correlation between basal βPS localization and κ_{basal} (Fig. 2H, H'). A scatter plot was used to plot the variation of κ_{basal} with changes in βPS for multiple discs belonging to two different stages of development. Points were color-coded based on their position along the pouch AP axis (medial: blue or lateral: red). Two straight lines were fit to study the correlation between βPS and κ_{basal} specific to pouch medial and lateral domains, and the corresponding domain-specific R^2 values have been indicated as an inset within the plots.

Additional method details for Fig. 3. An in-house pipeline (Supplementary text S-4.1) was used to quantify the basal curvature (κ_{basal}) for multiple discs belonging to 72 and 96 h AEL of larval

development (Fig. 3A", B"). The solid line indicates the averaged κ_{basal} value for multiple discs, while the shaded area indicates the standard deviation in prediction. The AP axis was further subdivided into three equal regions based on the overall length of the basal surface. The middle region corresponds to the medial domain, while the remaining two regions correspond to the lateral domains. Next, we calculated the ratio of the average curvature in the lateral ends to the medial domain ($\bar{\kappa}_{basal,lateral}/\bar{\kappa}_{basal,medial}$, Fig. 3D-ii). This ratio was computed over multiple discs whose average value was plotted as a bar graph. The ratio for individual samples is plotted as a scatter plot overlaid over the bar. Further, we use the same pipeline to calculate the same ratio ($\bar{\kappa}_{basal,lateral}/\bar{\kappa}_{basal,medial}$) for the simulation test cases that have been plotted as a bar graph on the right of the experimental data (Fig. 3D-ii).

For Fig. 3F-I, fluorescent intensities along the apical, basal, or lateral cell sides were plotted against the distance of the point from the center of the pouch. It was further normalized by averaging the overall length of the measured axis. Averaged fluorescence intensities and the standard deviation in prediction were plotted to visualize the spatial patterning. Analysis of several samples belonging to multiple stages of development shows temporal changes in spatial patterning of cytoskeletal regulators along the AP axis. Detailed steps for image normalization and intensity quantification can be found in Supplementary text S-4.2.

Additional method details for Fig. 4. To downregulate βPS expression, the GAL4/UAS[2] system was used to express an RNAi against *mys*, a beta subunit of the integrin dimer. The MS1096-Gal4 driver was used to drive the expression of mys^{RNAi} spatially, leading to more βPS inhibition in the dorsal compartment than the ventral side of the wing imaginal disc. A βPS antibody staining was carried out to validate the mutation. DAPI and Phalloidin dyes were used to visualize changes in cell shape (Actin) and nuclear positioning (Nuclei). Cross sections along the indicated DV axis were analyzed for these genetic perturbations (Fig. 4B, B').

We also used an MS1096-Gal4 driver to knock down Rho1 expression in the dorsal compartment of the wing disc with a commercially available UAS-$Rho1^{RNAI}$ line. DAPI and Phalloidin dyes labeled the nuclei and Actin, respectively. The cross-section along the DV axis was analyzed (Fig. 4C, C'). We also looked into how perturbations in βPS and Rho1 expression affect pMyoII. The details for these perturbations can be found in text corresponding to Supplementary Fig. 10.

An in-house MATLAB[1] code (Supplementary text S-4.2) was used to calculate the average height of cells for dorsal and ventral compartments of the cross-sections analyzed. Differences between the average height of dorsal and ventral compartments (ΔH) was computed and then normalized by the average height of the lateral compartment ($H_{Lateral}$) for multiple samples across all the genetic perturbations analyzed (Figure 4D, D').

Additional method details for Fig. 5. Unless otherwise indicated, cross sections along the AP axis for wing imaginal discs belonging to 72 h and 96 h AEL larval stages were analyzed. The proximity of nuclei with respect to the basal surface ($\bar{d}_B$) has been defined as the ratio of the distance of nuclei from the basal surface (d_B) over the sum of the distances of nuclei from apical (d_A) and basal surfaces (d_B). Details about the quantification pipeline are mentioned in

Supplementary section S-4.3 (Supplementary Fig. 25). As described in previous sections, the cross-section was subdivided into three regions, i.e., one medial and two lateral regions. Nuclei in each subregion were identified, and their proximity to the basal surface was calculated. We then calculated the average ($\bar{d}_B$) value for nuclei belonging to the different subregions. We use the ratio of average ($\bar{d}_B$) in the medial to lateral regions of the wing disc as a metric of comparison with results obtained using the computational model. The average value of this ratio calculated over multiple samples for discs belonging to 72 h and 96 h AEL larval stage of development has been plotted as a bar graph (Fig. 5B). The data from individual samples has been plotted as a scatter plot overlaid over the bar graph.

We also used an MS1096-Gal4 driver to express UAS-mys^{RNAi} to knock down βPS predominantly in the dorsal compartment of the wing disc (Fig. 5D). The disc was additionally stained with βPS antibody to validate the mutations. The nuclei for the mutant have been color-coded with their relative distance from the basal surface of the pouch ($\bar{d}_B$). Details about the quantification pipeline for nuclear positioning can be found in Supplementary text S-4.3.

Additional method details for Fig. 7. The GAL4/UAS[2] system was used to express dominant negative form of insulin receptors (InsR^{DN}) in the posterior compartment of the wing imaginal disc using an engrailed-Gal4 driver (Fig. 7 A-ii, B-ii, ii'). The engrailed-Gal4 also expresses a UAS-GFP marker to fluorescently label the posterior half, i.e., the region of perturbation. The parental engrailed-Gal4 driver was used as a control in addition to the internal control (Fig. 7 A-i, B i-i'). Wing imaginal discs belonging to 100-120 h AEL (wandering larvae, early 3rd instar) were dissected and fixed. The AP axis of the pouch was analyzed for these genetic perturbations. For both control and mutant samples, κ_{basal} was quantified using the in-house pipeline described in Supplementary text. S-4.1 (Fig. 7C-i). The average basal curvature (κ_{basal}) along with the standard error in mean have been plotted for points sampled on the basal surface. The x-axis indicates the normalized distance of the point from the pouch center (ℓ / L_{AP}). The normalization was carried out by dividing the distance by half of the length of the basal surface (L_{AP}). A student t-test was used to compare the average curvature profiles for control (en Gal4) and disc expressing en>InsR^{DN} samples in the anterior and posterior compartments respectively. The t-statistic (t), along with the p-value (p) for the test have been indicated on the top panels of the plot. Further, a Cohen's d (d) has also been indicated to measure the effect of sample sizes in statistical analysis. We also used our in-house pipeline to calculate the average height of the anterior ($H_{Anterior}$) and posterior ($H_{Posterior}$) compartments of the control (en-Gal4) and perturbed (en>InsR^{DN}) samples. We next calculated the difference between $H_{Anterior}$ and $H_{Posterior}$ and normalized it by $H_{Anterior}$. The quantity was calculated for multiple samples and has been visualized as a box plot (Fig. 7C-ii). A student t-test was used to compare the average curvature profiles for control (en Gal4) and disc expressing en>InsR^{DN} samples (***:$p<0.001$, **:$0.001<p<0.01$, *:$0.01<p<0.05$). Furthermore, antibody staining for βPS (Fig. 7B ii-ii'), Rho1 (Fig. 7D, D') and pMyoII (Fig. 7E, E') were carried out to measure changes in cytoskeletal regulation. We then calculated the difference in average fluorescence levels of the protein studied between the posterior and anterior compartments. Once this difference was computed, it was normalized by the average fluorescence of the anterior compartment for both the Gal4 control and genetically perturbed en>InsR^{DN} samples (Fig. 7F i-iii). Statistical significance of the differences between the

control and mutant group were assessed using a t-test (***:$p<0.001$, **:$0.001<p<0.01$, *:$0.01<p<0.05$).

We expressed the constitutively active form of InsR ($InsR^{CA}$) in the whole wing imaginal disc using a nubbin-Gal4 driver (Semi AP axis cross section visualized in Fig. 7G'). The progeny was grown at $18^{o}C$ as it was lethal at $25^{o}C$. The control for this experiment were the wing discs belonging to the parental nubbin-Gal4 driver grown at $18^{o}C$ (Fig. 7G). Expression of $InsR^{CA}$ caused a substantial increase in tissue size along with an increase in proliferation. Since the whole pouch was out of the sensor area of the microscope, we imaged half of the disc at a time (medial to anterior and medial to posterior) (Fig. 7G, G'). The discs were stained with βPS to measure cell shape changes. Half of the DV section at a time was analyzed for both control and mutant cases (Fig. 7G-H). Quantification of κ_{basal} (Fig. 7H i) and H (Fig. 7H ii) was carried out as described in the previous paragraph of this section.

GAL4/UAS system was used to express the constitutively active form of Dpp receptors, Thickveins (Tkv), in the posterior compartment of the wing imaginal disc using an en-Gal4 driver (Fig. 7I). Samples dissected from 100-120 h AEL (physiological stage: wandering 3rd instar larvae) were fixed. DAPI was used to label the nuclei. A PMAD antibody staining was performed to validate the UAS-Tkv^{CA} line (Supplementary Fig. 17C, C'). A βPS (Fig. 7I) and pMyoII (Fig. 7J) antibody staining was also carried out to measure changes in cytoskeletal regulation and tissue geometry. The cross-section along the AP axis was analyzed for the genetic perturbations.

We also used the en-Gal4 driver to overexpress Myc[3], a transcription factor contributing towards cell growth, in the posterior compartment of the wing imaginal disc (Fig. 7K). Wing imaginal discs belonging to 100-120 h AEL were physiologically staged. Wandering 3rd instar larvae were dissected, and the wing imaginal disc was fixed. A βPS and pMyoII antibody staining was carried out to measure changes in tissue shape while DAPI was used to label nuclei (Fig. 7K, L). Cross sections along the AP axis were analyzed for the genetic perturbations. For both of the genetic perturbations, quantification of κ_{basal} (Fig. 7M) and fluorescence intensities of cytoskeletal regulators (Fig. 7N, O) were carried outas described in the first paragraph of this section. Additional methods for quantification of basal curvature, tissue height, and expression of cytoskeletal regulators from the imaging data can be found in Supplementary section S4 of the text.

S2. Supplementary results and figures

S2.1 Integrin (βPS) and Collagen IV (ColIV) co-localize with Actin within the cortical region of a dividing cell. Wing imaginal disc belonging to early 3rd instar larval stage of development was dissected and fixed. A phosphohistone H3 (PH3) and a β-Integrin (βPS) antibody staining were carried out to measure the expression of integrins around the dividing cell. Based on the PH3 and nuclei markers (DAPI), cells belonging to different stages of division undergoing interkinetic nuclear migration were selected (Supplementary Fig. 1A). Optical cross-sections along the division axis were analyzed. It can be seen that βPS is initially localized near the filamentous actin tail of the dividing nuclei. As the nuclei move towards the apical surface for

division, its accumulation near the cell cortical region increases. Finally, when the cell has divided βPS localizes more towards the boundary of the daughter cells.

Wing imaginal discs from the early 3rd instar larval stage were dissected and fixed. DAPI and Phalloidin were used to label the nuclei and Actin, respectively (Supplementary Fig. 1B). An antibody staining against anti-Collagen IV was carried out to visualize spatial patterning of Collagen IV (ColIV) within the fixed tissue. Based on the data from Actin and nuclei fluorescence channels, a couple of representative mitotic nuclei were selected within the pouch (Supplementary Fig. 1B). An optical slice along the long axis of the dividing cells was taken to have a lateral perspective of ColIV during mitosis (Supplementary Fig. 1B-ii). A major highlight of a dividing cell is the presence of higher levels of Actin around the cortical region of a dividing cell[5]. Along with Actin and Integrin (Supplementary Fig. 1A), ColIV (Supplementary Fig. 1B-ii) also colocalizes at the cortical region of the dividing cell.

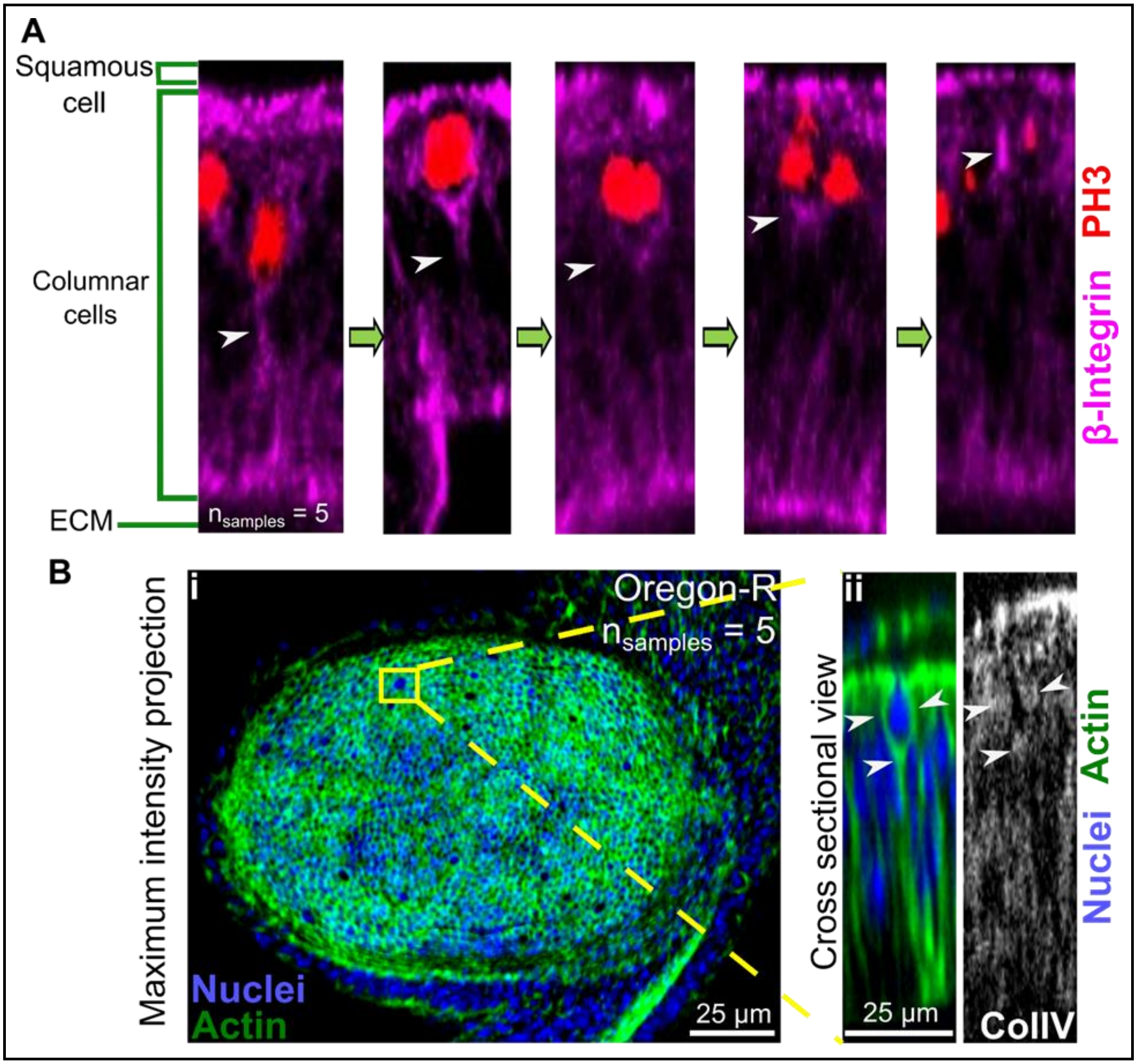

Supplementary Figure 1. Localization of βPS and CollV around a dividing cell. (A) Cross-sectional view of a wing imaginal disc showing localization of βPS around a dividing cell. Different images from left to right represent different stages of interkinetic nuclear migration. Discs are labeled with phosphohistone-h3 (PH3) to mark the dividing cells. Fluorescent labels have been indicated as an inset. Sample sizes have been indicated as a bottom inset. **(B) (i) (ii)** Cross-sectional view of wing cells undergoing mitosis. Discs are labeled with Collagen IV (CollV) to visualize its expression around a dividing cell. Sample sizes have been indicated on the top-right inset.

S2.2 The wing imaginal disc is flat across the AP axis at earlier stages of development (<72h AEL): Wing imaginal discs belonging to earlier stages of development were dissected and fixed. An antibody staining against Actin, pMyoII and βPS was carried out to study cytoskeletal regulation at early developmental stages (Supplementary Fig. 2). Although the tissue is flat, the cell height is qualitatively patterned across the AP axis. In particular, the cell height is higher at the middle as compared to the lateral ends of the pouch (Supplementary Fig. 2A). Moreover, both βPS (Supplementary Fig. 2A') and pMyoII (Supplementary Fig. 2A") are qualitatively polarized to the basal membrane of the pouch cells. Specifically, βPS is more localized near the central domains.

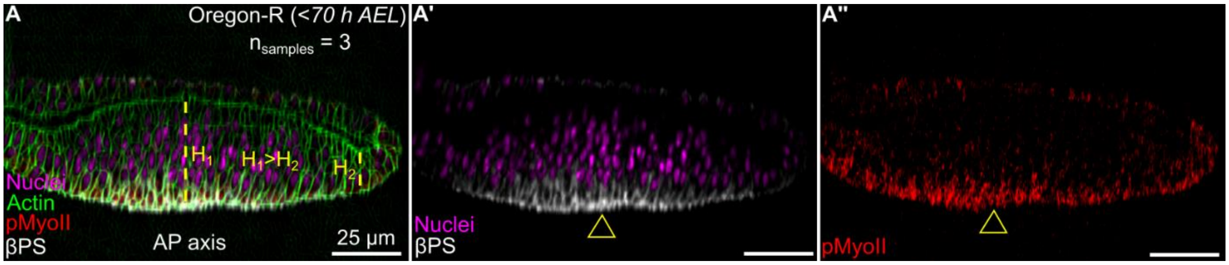

Supplementary Figure 2. Cytoskeletal regulation at earlier stages of development. Wing imaginal discs belonging to <72 h AEL larval stage of development were dissected and fixed. **(A)** Optical slice along the AP axis showing localization of Nuclei, Actin, **(A')** βPS and **(A'')** pMyoII. Sample sizes have been indicated on the top-right inset of (A).

S2.3 Correlation between $pMyoII_{apical}$ / $pMyoII_{basal}$ and local cell height (H) increases with the age of the disc. The gradient of H across the DV axis increases with the age of the disc. We used the image processing pipeline described in Supplementary section S4.2 to calculate the ratio of apical to basal fluorescence of pMyoII for 90 discretized cells along the AP axis. We also calculate the average lateral cell heights for the discretized cells to evaluate the correlation between $pMyoII_{apical}/pMyoII_{basal}$ and local cell height (H) (Supplementary Fig. 3A). A straight line is fit using scikit-learn[4]. An averaged R^2 value calculated over multiple samples denoted by R^2 (l) and an R^2 value for the combined data denoted by R^2 (g) from multiple samples are presented as separate insets in the plot (Supplementary Fig. 3D i-v). Different colors indicate data from different samples. We also carry out an F-test to estimate the statistical significance of the fit model[5,6]. The p-values of the F-test have been indicated as insets. The average R^2 value for discs belonging to late developmental stages (≥ 84 h AEL) is greater than that of younger discs

(< 84 h AEL). Moreover, the linear fits are statistically significant only for discs belonging to 84-96 h AEL.

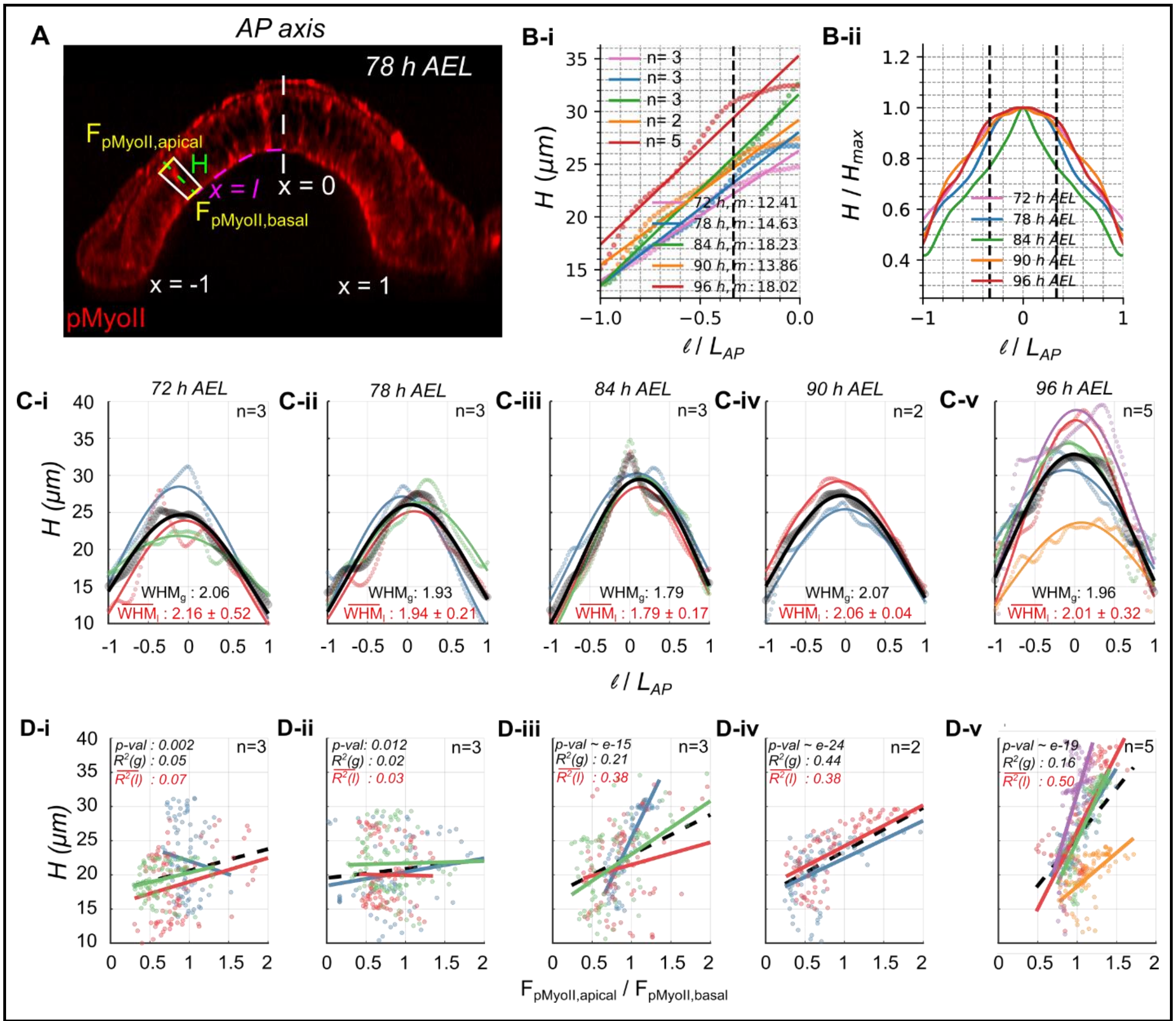

Supplementary Figure 3. (A) The wing disc cross section was discretized into 60 computational cells as described in Supplementary section S-4.2. $F_{Myosin,apical/basal}$ and indicates the fluorescence intensity of pMyoII in the apical and basal cell surface of the computational cell, respectively. l denotes the distance of the cell along the basal surface from the pouch center. The distance is further normalized by dividing l by half of the length of the pouch basal surface (L). **(B-i)** Plot showing the variation of cell height between the lateral and medial pouch domains. Each trend line in different colors represents averaged height values for discs at varying stages of development. The sample size associated with the averaging has been included as the top right inset. Further, a straight line was fitted to the trend, and the slope of the fitted lines across different development times has been reported within the plot. **(B-ii)** Plot showing normalized average height across the AP axis. Lines are color-coded based on the developmental stage. **(C)** Plot showing variation of cell height across the DV axis. Solid lines indicate the Gaussian model fit, while points (transparent) are the actual cell heights derived from the imaging data. The solid black line indicates the model fit for the combined data from multiple samples. WHM represents width of the curve

at half maximum where the height was fitted to a curve to create statistical models of height regulation. Sample sizes have been indicated on the top-right inset of each plot. **(D)** Correlation between $pMyoII_{apical}/pMyoII_{basal}$ and cell height H for discs belonging to different stages of development. Different colors indicate data from different samples. Sample sizes have been indicated on the top-right inset of each plot. A straight line is fit to model correlations for data from individual samples (color lines). Average R^2 values of the fits have been indicated as R^2(l). A linear model is next fit to the aggregated data using multiple samples (black dashed line). R^2(g) represents the R^2 value of the model fit.

Next, we analyzed the variation of cell height across the AP axis (Supplementary Fig. 3B, C). We first plotted the normalized cell height along the pouch AP axis for discs belonging to different developmental stages. The normalization was carried out by dividing the height of each cell by the maximum cell height (Supplementary Fig. 3B-ii). MATLAB[1] was then used to fit Gaussian models for modeling cell height across the AP axis for each sample across different developmental stages (Supplementary Fig. 3C i-v). Different colors are used to indicate data and models for different samples within the same developmental stage. We additionally evaluated the width at half maximum (WHM) for all the fits and reported the averaged WHM across all of the samples and also for a fit across the entire data. There is no statistically significant observed change in WHM across the age of the disc. Lastly, to study how the cell height varies across the AP axis during development, we fit straight lines to averaged cell height profiles across half of the pouch (Supplementary Fig. 3B-i). An analysis of slopes of fits reveals that the slope is greater for discs belonging to late development stages (>= 84 h AEL) as compared to the early development stages (<= 78 h AEL). Thus, the medial tissue height increases at a greater rate as compared to the lateral height of the tissue during development.

S2.4 Colocalization of Actin, pMyoII and βPS increases with the age of the disc. Discs belonging to two different stages of development (72 h AEL (Supplementary Fig. 4a), and 96 h AEL (Supplementary Fig. 4b) larval stages) were fixed, and an immunohistochemistry assay was carried out to label the expression of cytoskeletal regulators, including Actin, pMyoII and βPS. We next discretized the pouch AP axis into 90 cells as described in Supplementary section S4.2. The averaged apical, basal, and lateral fluorescence intensity of Actin, pMyoII and βPS was calculated for the cells. To remove background noise in the imaging data, a rolling ball background subtraction[9] was performed using a 50-pixel ball radius in ImageJ[7]. Further, the location of each discretized cell within the AP axis was defined as its normalized distance from the center of the AP axis. Normalization was carried out by dividing the distance of a particular discretized cell by half the curve length of the basal surface.

We first analyzed Actin, pMyoII and βPS expression profiles across the AP axis at the apical, basal, and lateral sides of cells (Supplementary Fig. 4a, b-D). The x-axis of the plot is the normalized location along the basal surface. The solid line indicates the mean fluorescence of the measured cytoskeletal regulators ((i) βPS, (ii) pMyoII, (iii) Actin), while the shaded region indicates the standard deviation in fluorescence reported over multiple samples. At earlier stages of development, pMyoII and βPS are predominantly basal (Supplementary Fig. 4a-Di, ii). Moreover, integrin is localized primarily at the medial pouch region. In fact, it is like the expression at earlier developmental stages, as shown qualitatively in Supplementary Fig. 2. However, at later stages

of development, pMyoII in the medial pouch domain starts to become apical. In contrast, both pMyoII and βPS start to acquire more lateral expressions in the basal surface of the pouch (Supplementary Fig. 4b-Di, ii).

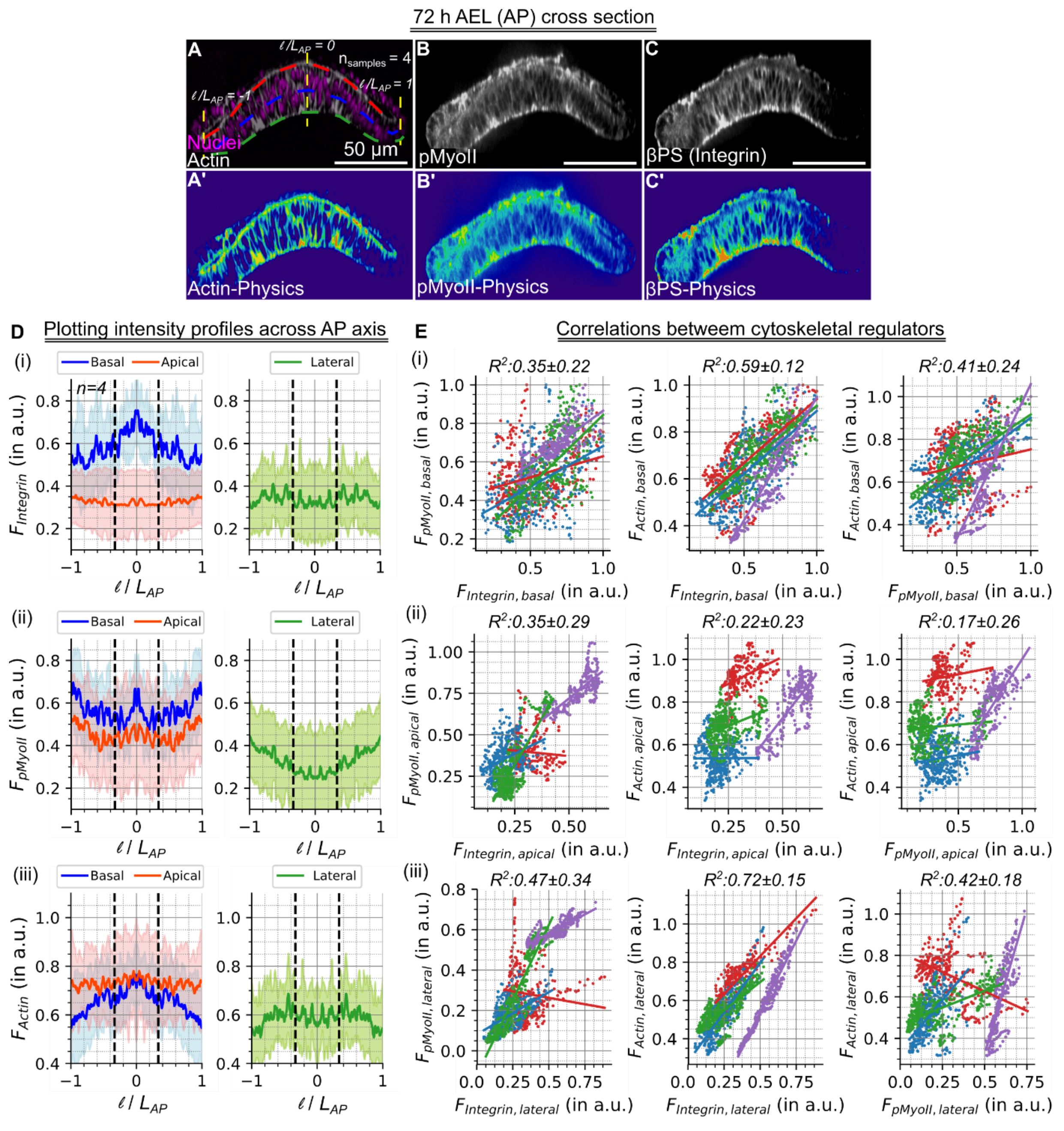

Supplementary Figure 4a. Actin, Myosin, and Integrin at earlier stages of development are expressed predominantly near the basal surface of pouch cells. Integrin correlates strongly with Actin. (A-C) AP cross-section of wing imaginal discs staged to 72 h AEL. Fluorescent labels are indicated in the bottom left panel of each cross-section. Fluorescent intensity of Actin, Myosin, and Integrin were calculated over the apical, basal, and lateral sections of the disc proper (DP) cells for several samples (n = 5) **(D) (i- iii)** Variation of Integrin, Myosin and Actin localization across the pouch apical, basal and

lateral surface. **(E)** Correlations between Actin, Myosin, and Integrin at the pouch cell's apical, basal, and lateral surfaces.

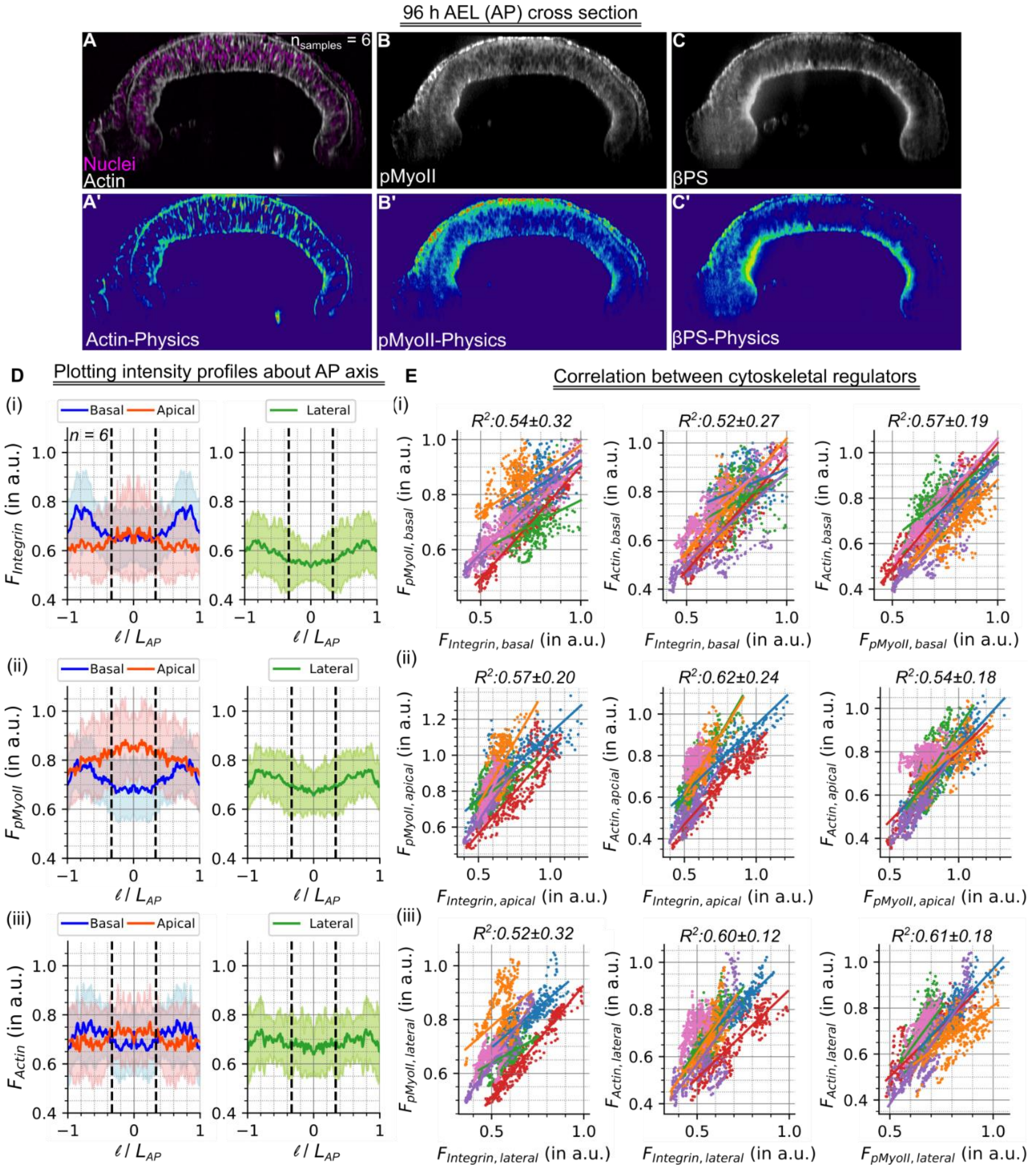

Supplementary Figure 4b. Actin, Myosin, and Integrin correlate strongly and are predominantly expressed in the basal-lateral region of the pouch. (A-C) AP cross-section of a wing imaginal disc dissected from larvae staged to 96 h AEL. Fluorescent labels are indicated in the bottom left panel of

each cross-section. Fluorescent intensity of Actin, Myosin and Integrin were calculated over the apical, basal, and lateral sections of the pouch for several samples (n = 7) **(D) (i - iii)** Variation of Integrin, Myosin and Actin localization across the apical, basal and lateral surface of columnar pouch cells. **(E)** Correlations between Actin, Myosin, and Integrin at the apical, basal and lateral surfaces of columnar pouch cells.

We next evaluated correlations between the extracted fluorescent intensity of Actin, pMyoII, and βPS in the apical, basal, and lateral surfaces of the pouch, respectively (Supplementary Fig. 4a, b-E). Normalized fluorescence intensities of two components at a time ((βPS, pMyoII), (βPS, Actin), (pMyoII, Actin)) were plotted for multiple samples, and a straight line was fit using the LinearRegression library within scikit-learn[4]. R^2 values across multiple samples were averaged and reported on top of each plot and used as a metric to evaluate the strength in colocalization. Different colors within the plot represent data from different samples. We report an increased averaged R^2 values of the fit for all of the correlations along the basal surface as the disc ages (Supplementary Fig. 4a, b-E-i).

S2.5 Proliferation decreases with an increase in pouch size. In this section, we report a decrease in cell proliferation with an increase in the age of the disc. The obtained result agrees with previous literature studies[8]. Wing imaginal discs were dissected from larvae of different sizes belonging to different stages of development and proliferating cells were marked with anti-PH3. The mitotic index was next defined as the ratio of the area of cells that are marked by PH3 over the area of the pouch (Supplementary Fig. 5A). A 2D scatter plot was plotted with the X-axis representing the pouch area and the Y-axis the mitotic index (Supplementary Fig. 5B). An exponential decay model was next fit to the data using MATLAB's[1] fit function. The model details along with the goodness of fit indicated by an R^2 value have been included as a plot inset. The tendency of cells to divide decreases as the size of the pouch increases.

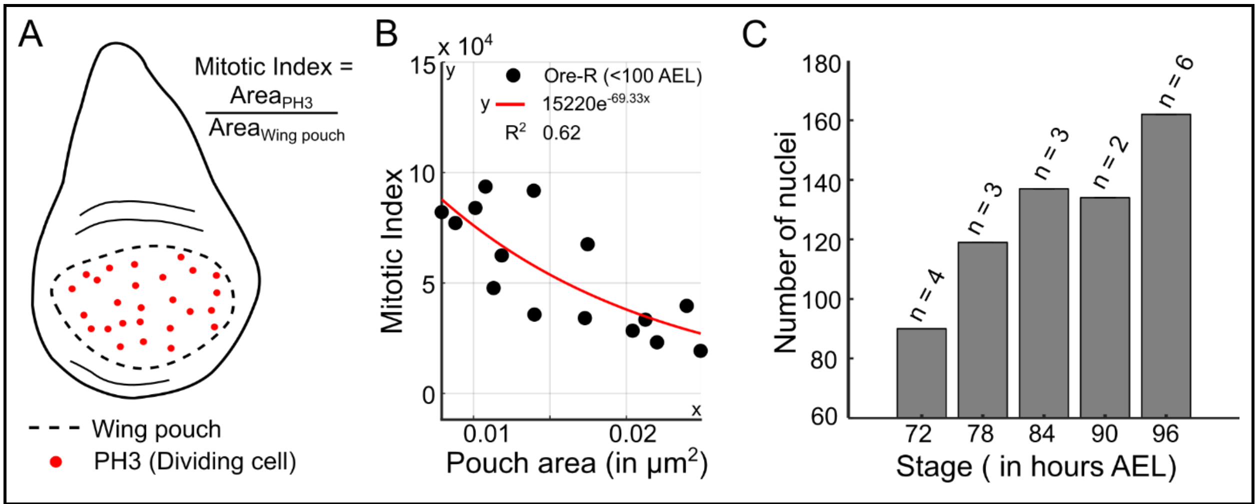

Supplementary Figure 5. Cell proliferation decreases with the age of the pouch. (A) Mitotic index is defined as the ratio of the area acquired by cells expressing Phospho Histone-H3 (PH3) over the area of the wing disc pouch. **(B)** Plot showing the variation of the mitotic index with respect to the pouch size.

The red line indicates an exponential fit obtained through regression in MATLAB. **(C)** Plot showing the distribution of an average number of nuclei along the pouch AP axis with the age of the disc.

S2.6 In silico model scenarios of patterned cytoskeletal regulation across the AP axis. Multiple model parameters representing different functions of cytoskeletal regulation were patterned across the pouch's AP axis. Values of parameters were kept separate for the pouch medial and lateral domains and across the apical and basal surfaces depending upon the parameter varied. In total, we varied eight model parameters generating a total of 15 parameter profiles (Supplementary Fig. 6). For instance, in Case I, we varied the actomyosin contractility in the model by increasing the apical ($k_{api,cont}$) and basal ($k_{bas,cont}$) contractility in the medial domain of the pouch and also increasing $k_{bas,cont}$ more than $k_{api,cont}$in the lateral ends. The increase in the center was higher than that away from the center. This recapitulates the expression of pMyoII at 96 h AEL developmental stage, where we report higher apical contractility in the pouch medial region and a higher basal contractility in the lateral ends of the pouch (Fig. 3F). We also varied the parameters associated with the basal ECM stiffness ($k_{ecm,c}$), the membrane tension ($k_{memb,basal}, k_{memb,lateral}, L0_{memb,lateral}, L0_{memb,basal}$) and the Integrin-based adhesion between the basal region of columnar cells and the ECM (k_{adhB}) (Supplementary Table 2). Cross sections of the simulation output have been shown along with the varying parameter profile across the AP axis on the top of each figure (Supplementary Fig. 6).

S2.7 Tissue local height is regulated by the difference in apical-basal contractility. To test if cell height is driven by the apical-basal stiffness of cells, we ran simulations with a differential patterning of contractility parameters across the apical and basal surfaces. First, we divided the tissue into three domains (a medial domain and two lateral domains) and perturbed $k_{api,cont}$ and $k_{bas,cont}$ in each of these domains (Supplementary Fig. 7A). The contractility multipliers used in each of the cases are listed in the table in Supplementary Fig. 7B. Note that the apical and basal contractility multipliers get multiplied to $k_{api,cont}$ and $k_{bas,cont}$, respectively, to increase or decrease the strength of the actomyosin contractile springs. Cases 1A and 1B are reproduced from Supplementary Fig. 6A, where actomyosin contractility was increased and decreased, respectively, in the medial domain of the pouch. Using Case 1A as a comparison, we increased both $k_{api,cont}$ and $k_{bas,cont}$ in the medial domain while ensuring that the ratio of $k_{api,cont}$ / $k_{bas,cont}$ remained constant (Case 1C) and also increased (Case 1D). Next, we used a multiple linear regression to further perturb $k_{api,cont}$ and $k_{bas,cont}$ (Cases 1E-1J) in the medial domain. Finally, in addition to varying the actomyosin contractility in the medial domain similar to Cases 1E-1J, we tested the effect of decreasing the basal contractility (by decreasing $k_{bas,cont}$) in the lateral domain of the pouch (Cases 1K-1V).

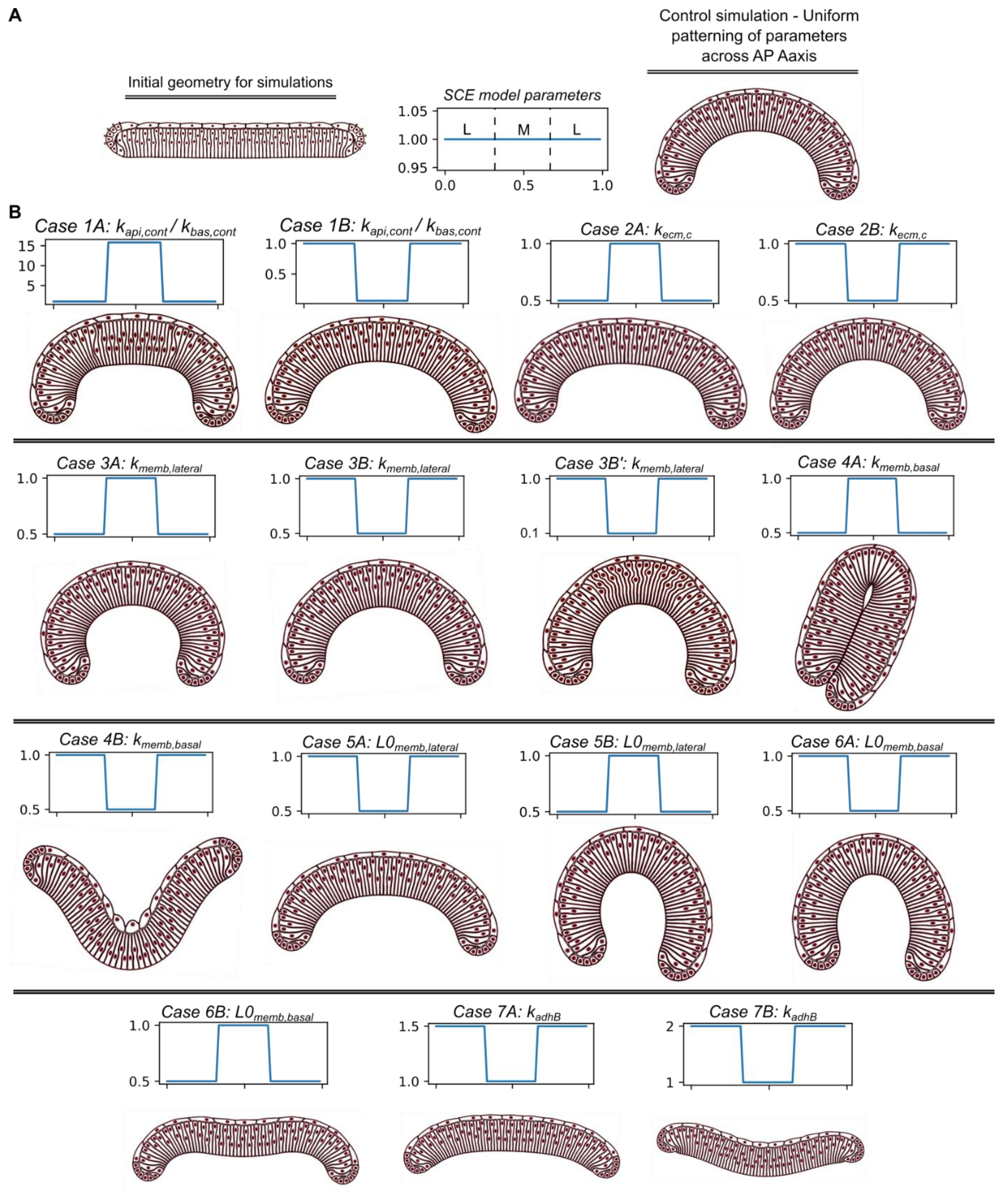

Supplementary Figure 6. Key parameters that impact the basal curvature of the tissue. (A-F) Through a series of cases, model parameters representing mechanical properties of cell cytoskeleton, actomyosin and adhesion were varied across the AP axis. The profiles of parameters are plotted above the final simulated shape.

Next, we carried out a quantitative comparison between phenotypic changes that occurred between the 72 and 96-hour discs and the simulation cases where actomyosin contractility was patterned across the AP axis, in contrast to the uniform patterned contractility in the control simulation (Supplementary Fig. 8). We first visualized metrics, including the ratio of lateral to medial basal curvature (Supplementary Fig. 8A-i), medial tissue height (Supplementary Fig. 8A-ii), and the ratio of nuclear positioning between the medial and lateral compartments (Supplementary Fig. 8A-iii). Compared to the control, all of the simulation cases led to an increase in tissue height and a basal shift of nuclei, consistent with our observations in the experimental data. However, only a few simulations resulted in the flattening of the midsection. We then visualized the differences in these phenotypic traits (Supplementary Fig. 8B). For experiments, the difference was calculated by subtracting these geometric features between the 96 and 72-hour discs. For the simulations, the morphological features of all cases were subtracted from the control case. Errors within individual metrics were normalized by dividing by the maximum reported error. Finally, we quantified the overall error by squaring and summing individual errors. Using this approach, we identified Case 1D as the case that matches all three metrics most accurately. Note that this case is presented as Case 1A' in Figures 3-5 of the main text.

To further explore the role of apical to basal contractility in cell height, we ran additional simulations. This time, the pouch was subdivided into five nested domains, namely: left (L), left medial (LM), medial (M), right medial (RM), and right (R). Perturbation of $k_{api,cont}$ and $k_{bas,cont}$ was done only in the medial domain of the pouch (Supplementary Fig. 9A-A''). The parameter values for the three different cases are listed in a table in Supplementary Fig. 9B. Initially, we assumed the contractility to be localized only on the basal surface. We first increased both $k_{api,cont}$ and $k_{bas,cont}$ without changing the ratio of $k_{api,cont}$ / $k_{bas,cont}$. Analysis of the height profiles showed an increase in levels of contractilities in the apical and basal surface without changing the ratio of the two increased cell height (Supplementary Fig. 9A', 9C). Next, we increased both $k_{api,cont}$ and $k_{bas,cont}$ in such a way that the increment also increased the ratio between the two. A slight change in height was observed as compared to the previous case (Supplementary Fig. 9A'', 9C).

A

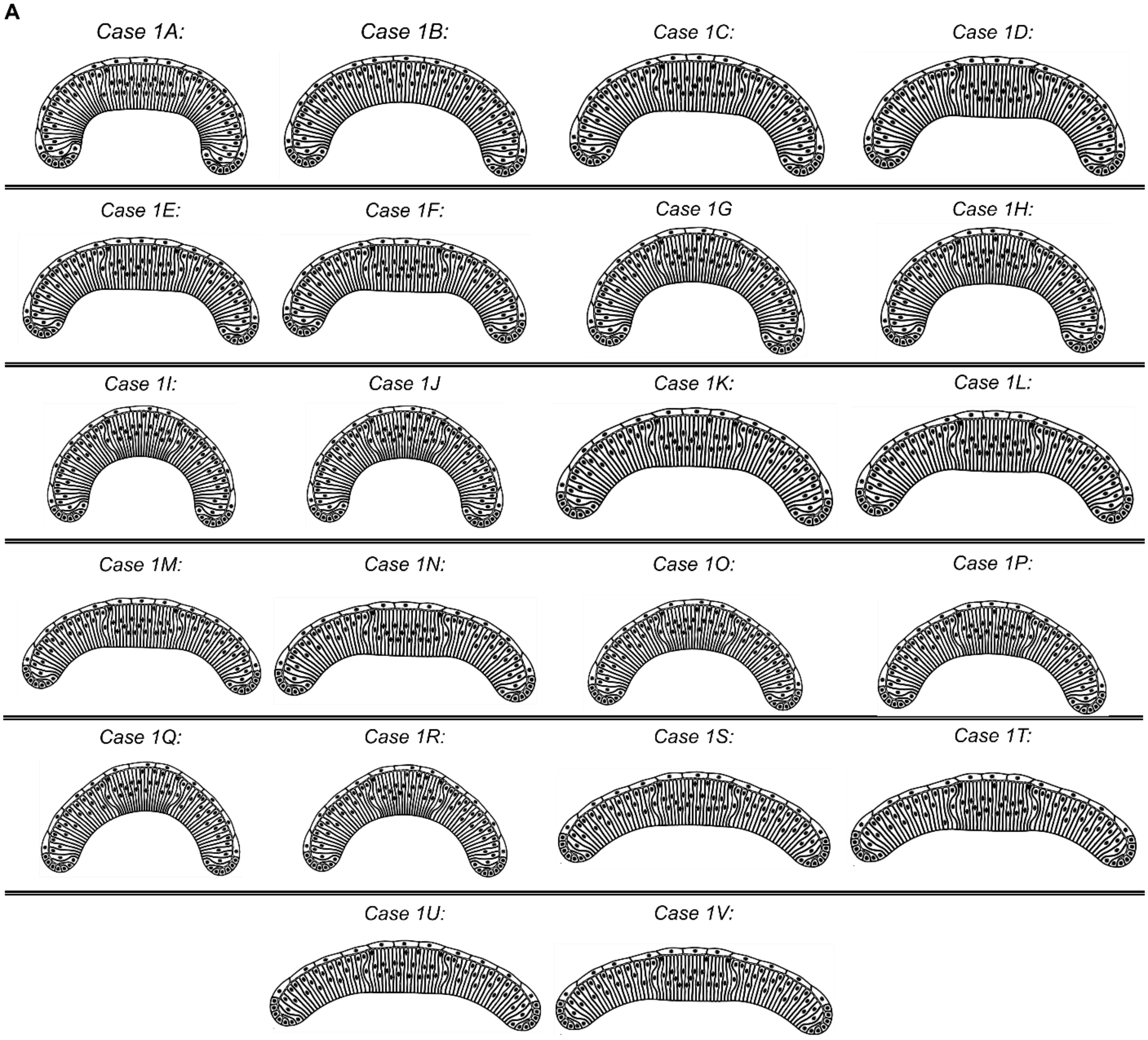

B

Contractility multiplier profile

		Lateral	Medial	Lateral
**Case 1A**	apical	0.0625	**1.333**	0.0625
	basal	1.5	**2.0**	1.5
**Case 1B**	apical	0.0625	**0.0029**	0.0625
	basal	1.5	**1.125**	1.5
**Case 1C**	apical	0.0625	**1.675**	0.0625
	basal	1.5	**2.5**	1.5
**Case 1D**	apical	0.0625	**2.61**	0.0625
	basal	1.5	**3.0**	1.5
**Case 1E**	apical	0.0625	**1.666**	0.0625
	basal	1.5	**2.219**	1.5
**Case 1F**	apical	0.0625	**2.0**	0.0625
	basal	1.5	**2.240**	1.5
**Case 1G**	apical	0.0625	**1.666**	0.0625
	basal	1.5	**3.856**	1.5
**Case 1H**	apical	0.0625	**2.0**	0.0625
	basal	1.5	**3.878**	1.5
**Case 1I**	apical	0.0625	**1.666**	0.0625
	basal	1.5	**4.948**	1.5
**Case 1J**	apical	0.0625	**2.0**	0.0625
	basal	1.5	**4.970**	1.5
**Case 1K**	apical	0.0625	**1.675**	0.0625
	basal	**1.0**	**2.5**	**1.0**

		Lateral	Medial	Lateral
**Case 1L**	apical	0.0625	**2.61**	0.0625
	basal	**1.0**	**3.0**	**1.0**
**Case 1M**	apical	0.0625	**1.666**	0.0625
	basal	**1.0**	**2.219**	**1.0**
**Case 1N**	apical	0.0625	**2.0**	0.0625
	basal	**1.0**	**2.240**	**1.0**
**Case 1O**	apical	0.0625	**1.666**	0.0625
	basal	**1.0**	**3.856**	**1.0**
**Case 1P**	apical	0.0625	**2.0**	0.0625
	basal	**1.0**	**3.878**	**1.0**
**Case 1Q**	apical	0.0625	**1.666**	0.0625
	basal	**1.0**	**4.948**	**1.0**
**Case 1R**	apical	0.0625	**2.0**	0.0625
	basal	**1.0**	**4.970**	**1.0**
**Case 1S**	apical	0.0625	**1.675**	0.0625
	basal	**0.5**	**2.5**	**0.5**
**Case 1T**	apical	0.0625	**2.61**	0.0625
	basal	**0.5**	**3.0**	**0.5**
**Case 1U**	apical	0.0625	**1.666**	0.0625
	basal	**0.5**	**2.219**	**0.5**
**Case 1V**	apical	0.0625	**2.0**	0.0625
	basal	**0.5**	**2.240**	**0.5**

Supplementary Figure 7. Variation of apical to basal actomyosin contractility impacts cell height. (A) Shapes obtained from varying actomyosin contractility in the pouch medial and lateral domains. Cases 1A and 1B are reproduced from Supplementary Fig. 6. In cases 1C and 1D, both apical and basal actomyosin contractility was increased in the medial domain. Linear regression was used to vary actomyosin in the medial region in cases 1E-1J. Finally, in cases 1K-1V, in addition to varying contractility in the medial domain (similar to cases 1E-1J), the effect of decreasing the basal contractility in the lateral domain is tested. **(B)** A table of the apical and basal contractility multipliers for each simulation case. Note that the apical and basal contractility multipliers get multiplied to $k_{api,cont}$ and $k_{bas,cont}$, respectively. The role of these multipliers is to increase or decrease the strength of the actomyosin contractile springs.

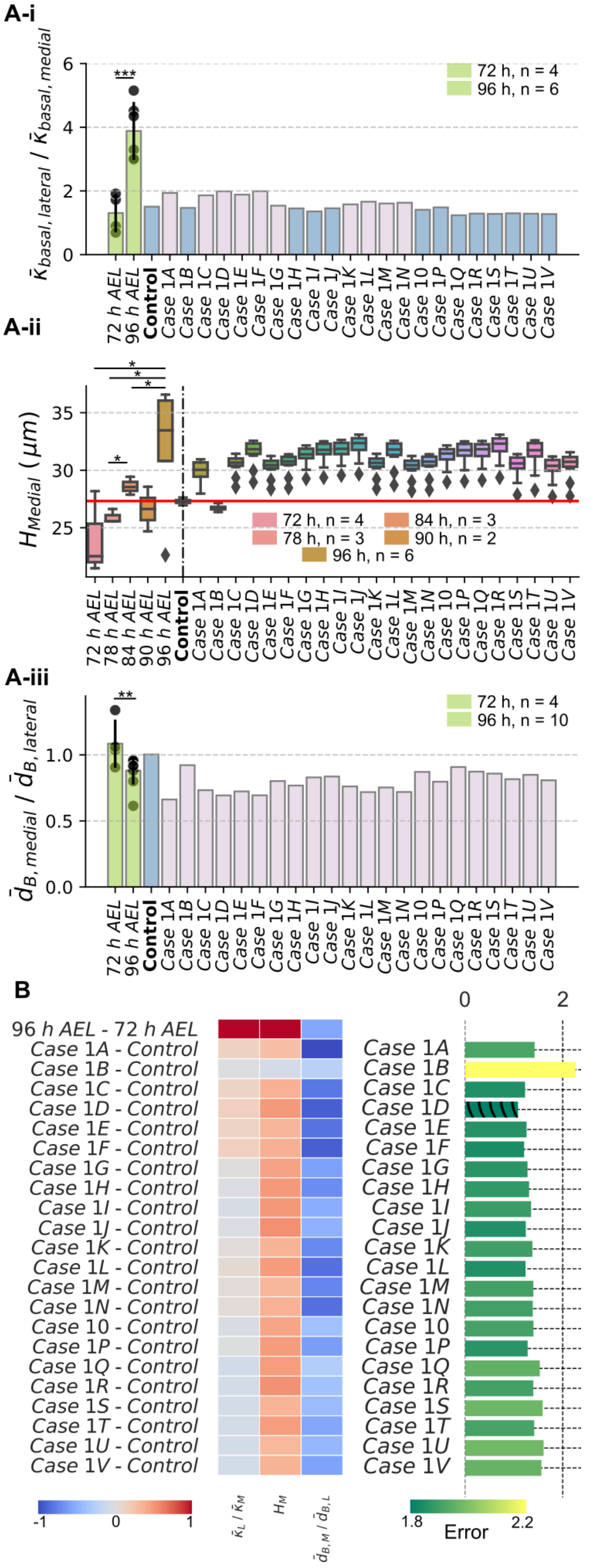

Supplementary Figure 8. Effect of variation of apical-basal contractility on basal curvature, cell height and nuclear positioning. (A) Bar graph visualizing comparison of **(i)** ratio of average lateral

curvature to basal curvature **(ii)** medial tissue height **(iii)** ratio of $\bar{d}_B$ in medial to lateral compartment for wing discs belonging to 72 and 96 h AEL of development and the simulations presented in Supplementary Fig. 7. **(B)** Differences between control and the perturbation simulations (y-axis) were normalized and compared with differences of measured properties (x- axis) between 72 h AEL and 96 h AEL (1st row). The combined errors from the three metrics were calculated through differences between the experimental and simulation data. The error bars are color-coded based on their values.

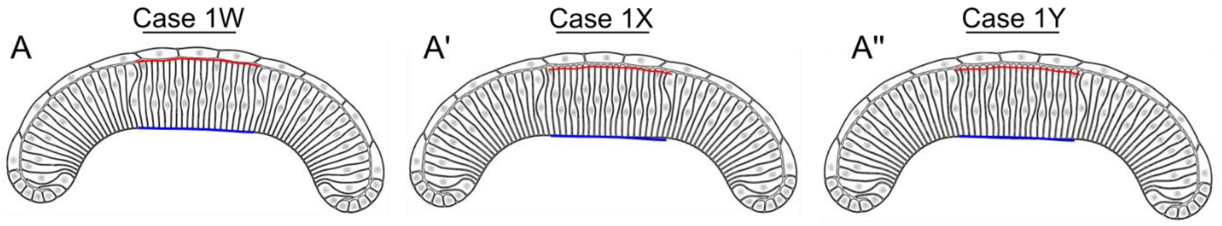

B

Contractility multiplier profile

		L	LM	M	RM	R
W	apical	0.06	1.25	**1.25**	1.25	0.06
	basal	1.5	1.5	**1.88**	1.5	1.5
X	apical	0.06	1.25	**1.56**	1.25	0.06
	basal	1.5	1.5	**2.4**	1.5	1.5
Y	apical	0.06	1.25	**2.19**	1.25	0.06
	basal	1.5	1.5	**2.62**	1.5	1.5

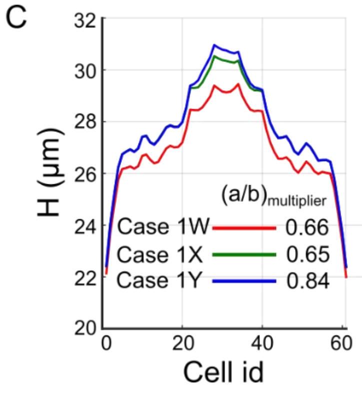

Supplementary Figure 9. Effect of apical-basal contractility profiles on cell height. (A-A") Stable shapes obtained because of varying actomyosin contractility in the pouch medial domain. In cases W-Y the pouch was subdivided into five domains: left (L), left medial (LM), medial (M), right medial (RM), and right (R) where the apical-basal contractility ratio was only varied in the medial (M) domain. **(B)** A table of parameters for the simulations run in this case study was organized according to the five domains (L, LM, M, RM and R). For each case W-Y, the top row represents the apical contractility multiplier that gets multiplied to $k_{api,cont}$ while the bottom row represents the basal contractility multiplier for $k_{bas,cont}$. These multipliers are used to increase the strength of the actomyosin contractile springs. **(C)** Height profiles in the tissue are plotted against the cell id (from left to right of tissue) for the different cases run in this study. The ratio of the apical to basal contractility multiplier $(a/b)_{multiplier}$ for the medial domain associated with each case in (B) has been included as part of the figure legend. Note that a represents the contractility multiplier for $k_{api,cont}$ while b represents the contractility multiplier associated with $k_{bas,cont}$.

S2.8 Knockdown of Integrin increases basal curvature while knockdown of Rho1 reduces basal curvature. *MS1096-Gal4* driver was used to differentially express RNAi against mys and Rho1 (Supplementary Fig. 10A-C). The Gal4 driver allows more inhibition in the dorsal compartment while decreasing its effect as one moves towards the ventral compartment. We used multiple samples to quantify the basal curvature of the pouch across the DV axis (Supplementary Fig. A'-C'). Compared to the control, expression of mys^{RNAi} in the dorsal compartment led to a sharp increase in tissue folding near the lateral ends of the pouch. In contrast, the expression of $Rho1^{RNAi}$ led to a reduction in basal curvature reflected by flattening of

the basal surface in the dorsal compartment (region of perturbation).

We also carried out immunostaining assays to see if the inhibition of mys and Rho in the wing disc effects pMyoII. Analysis of pMyoII fluorescence across the pouch DV axis for the *MS1096>Rho1*^{RNAi} genetic perturbations shows a decrease of pMyoII in the dorsal compartment of pouch where Rho1 activity was suppressed (Supplementary Fig. 10D, D'). Similarly, for *en>mys*^{RNAi}, we also report a reduction in pMyoII fluorescence in the posterior compartment where mys was inhibited (Supplementary Fig. 10E, E'). In summary, we qualitatively show that a loss in both Rho1 and mys can affect accumulation of pMyoII within the pouch.

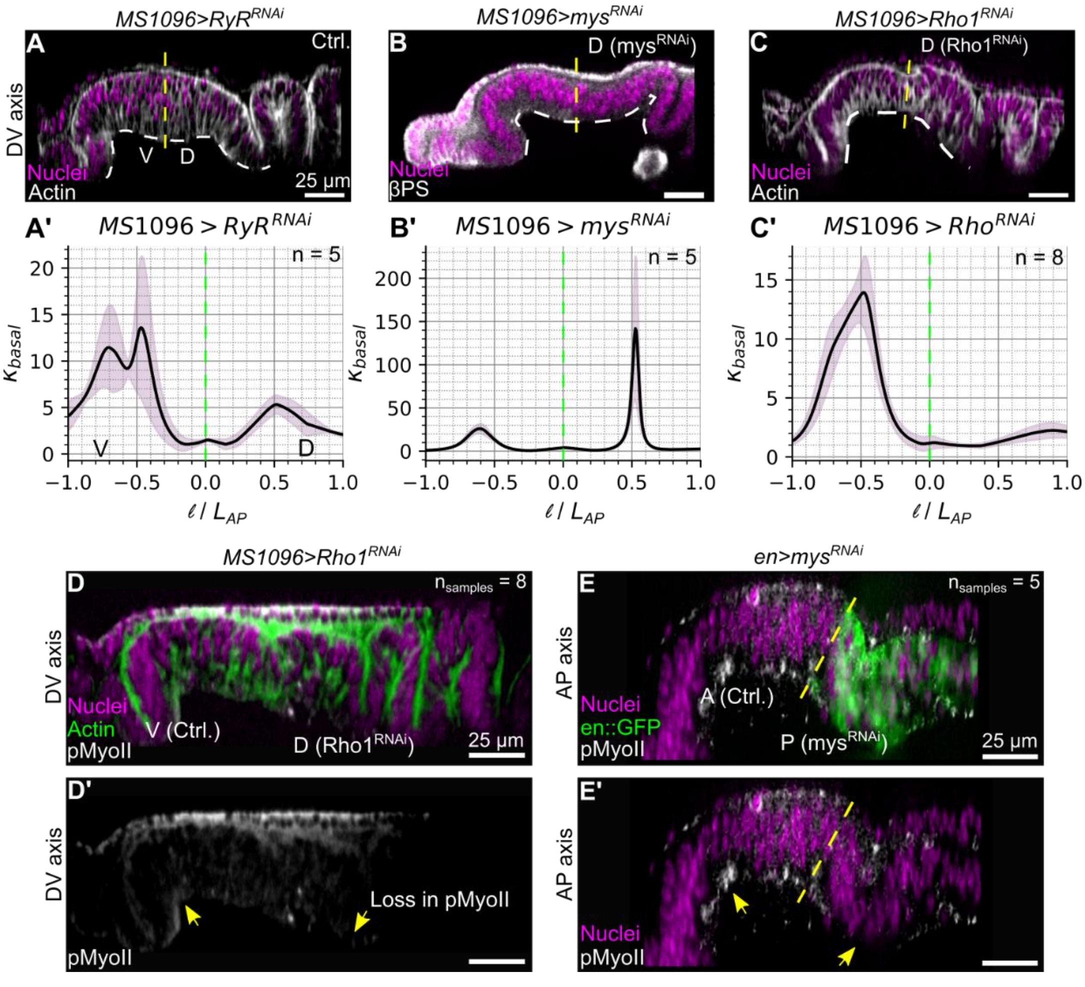

Supplementary Figure 10. Loss of Integrin increases while loss of Rho1 reduces basal curvature. MS1096-Gal4 driver was used to knock down mys and Rho1 in the dorsal compartment of the wing imaginal disc. Comparisons were done using the *MS1096>RyR*^{RNAi} control. **(A-C)** DV cross-sections of the pouch are shown. **(A'-C')** Quantification of basal curvature across the DV axis is shown. Solid line indicates the mean while the shaded region indicates the standard deviation of n predictions. Sample sizes have been indicated on the top-right inset of each plot. **(D, D')** DV cross sections for the *MS1096>Rho1*^{RNAi} genetic perturbations. **(E, E')** AP sections for *en>mys*^{RNAi} genetic perturbations. Fluorescent labels

have been indicated within the plot.

S2.9 A cell-specific increase in control volumes (Ω_0) causes an increase in cell height (H) without changing its gradient along the DV axis. In comparison to Case 8Csimulation listed in Figure 4E, G', we ran an additional simulation where we used a similar gradient to increase the cell volume of each cell by adjusting the maximum and minimum cell volumes such that the overall tissue volume was equal to the control simulation (Supplementary Fig. 11A, E). Compared to Case 8C, the height of cells in Case 8Dwas higher (Supplementary Fig. 11B). However, no qualitative difference in gradients of cell height across the AP axis is reported.

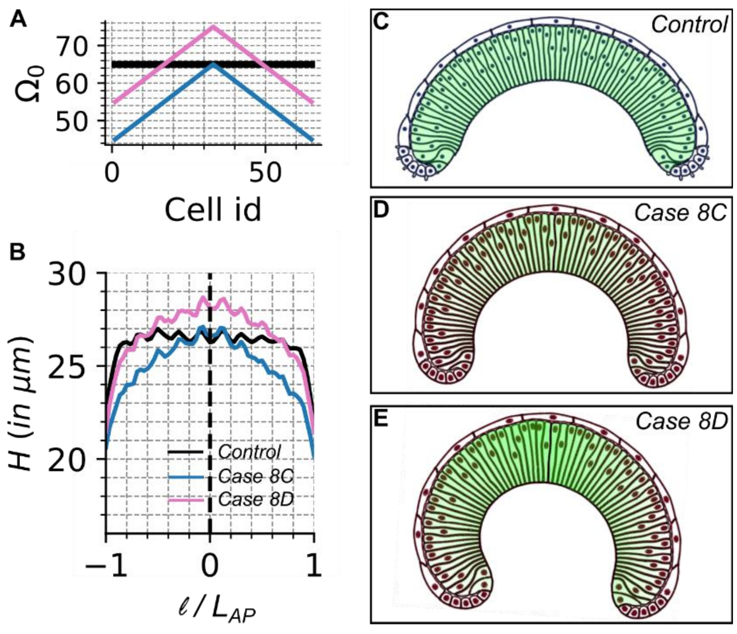

Supplementary Figure 11. Increasing cell volumes globally causes a global increase in the cell height (A) Plot showing variation of Lagrange multiplier (Ω_0) along the pouch AP axis for different case studies. **(B)** Plot showing variation of lateral cell height (H) along the pouch AP axis for the simulated cases. **(C-E)** Model outputs for the simulated case studies. The green shading indicates a qualitative patterning of Ω_0 across the pouch AP axis.

S2.10 Loss of Rho1 pushes the nuclei basally. *MS1096-Gal4* driver was used to inhibit Rho1 in the dorsal compartment of the wing imaginal disc (Supplementary Fig. 12A). Note that the Gal4 driver also leads to inhibition of Rho1 in the ventral compartment, but the degree of perturbation decreases while moving away from the DV compartment boundary. To study how inhibition of Rho1 affects nuclear positioning, we segmented the nuclei in the DV section of the pouch. We next quantified the proximity of nuclei with the basal surface by estimating $\bar{d}_B = \frac{d_B}{d_A + d_B}$ (S-4.3, Supplementary Fig. 25). As shown in Supplementary Fig. 12C, the nuclei are located much closer to the basal surface than the nuclei within the dorsal compartment (internal control).

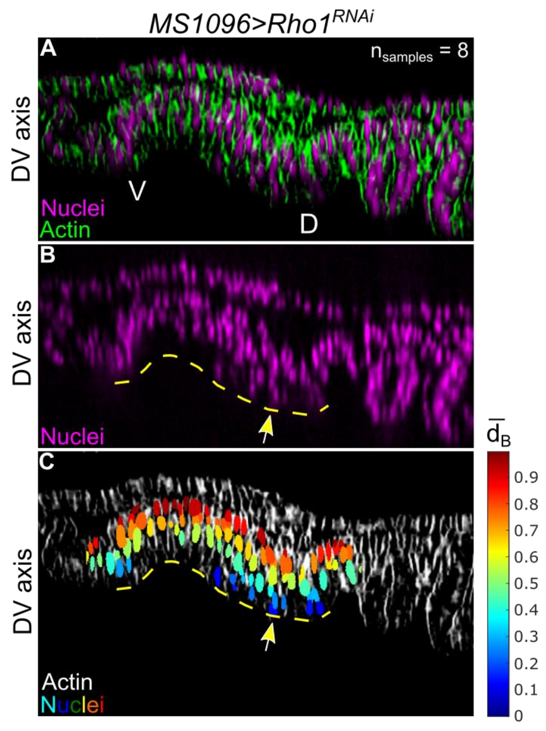

Supplementary Figure 12. Loss of Rho1 pushes the nuclei basally. (A, B) Inhibition of Rho1 in the dorsal compartment of the wing imaginal disc (*MS1096>Rho1^{RNAi}*). Fluorescent labels have been indicated as labels in the left bottom corner**. (C)** Nuclei were segmented and their proximity to the basal surface of epithelia is shown.

S2.11 Compartment-specific expression of InsR and Myc to increase proliferation results in two distinct phenotypes. We used an engrailed-Gal4 driver to inhibit proliferation in the posterior compartment by expressing the dominant-negative (DN) form of the *Drosophila* insulin receptor (InsR^{DN}) in the posterior compartment (Supplementary Fig. 13 A i-ii). We confirmed through a PH3 antibody staining that the downregulation of insulin signaling activity through the expression of InsR^{DN} reduced the number of mitotic cells[9] (Supplementary Fig. 13A i'-ii'). We also expressed the constitutively active form of insulin receptors (InsR^{CA}) in the posterior compartment of the wing imaginal disc (Supplementary Fig. 13A-iii). Expression of InsR^{CA} in the posterior compartment increased the number of mitotic cells in the pouch (Supplementary Fig. 13A-iii'). Wing imaginal discs were physiologically staged, and only 3rd instar wandering larvae were dissected. The *en-Gal4* driver has been used as a control for the comparison (Supplementary Fig. 13A-i, i'). We also examined cross-sections along the DV axis in the anterior (Supplementary Fig. 13C-i, ii) and posterior compartments (Supplementary Fig. 13C-i', ii'). Analysis of the cross-section reveals a similar observed increase in inwards bending upon expression of InsR^{CA} as observed in *Nubbin>InsR^{CA}* (Fig. 7G', H-i). Qualitatively, cross-sections taken in the posterior compartment have an increased inward bending near the pouch lateral domains.

We next overexpressed Myc in the dorsal compartment of the wing imaginal disc (*apterous-Gal4*

x UAS-Myc) (Supplementary Fig. 13D). Myc is a direct regulator of cell cycle and biogenesis[3], hence its overexpression increases in growth and cell proliferation. We next examined the cross-section along the DV axis, parallel to the AP boundary. Interestingly, very similar to *en>Myc* (Fig. 7K, K'), we see a sharp increase in βPS fluorescence level in the dorsal compartment along with a loss in basal curvature (Supplementary Fig. 13E). In summary, the analysis of the biological replicates aligns with the data, and the results are included in the main manuscript in Figure 7.

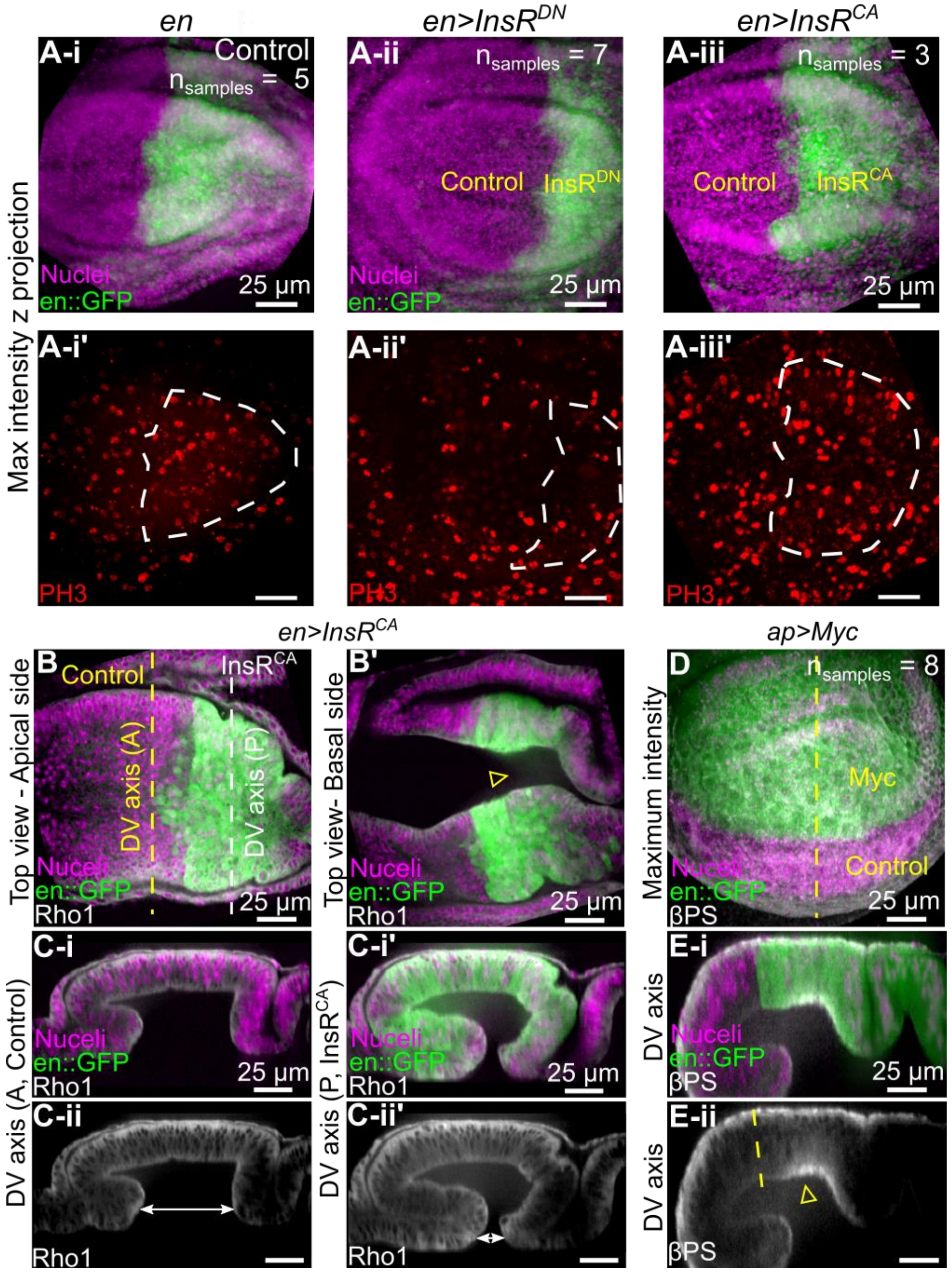

Supplementary Figure 13. Two distinct phenotypes resulting from compartment-specific expression of InsR and Myc to increase proliferation. (A) Maximum intensity projection of **(i-i')** control and **(ii-ii')** samples expressing dominant negative insulin receptor,

InsR^{DN}, and **(iii-iii')** samples expressing constitutively active insulin receptor, *InsR^{CA}* in the posterior compartment driven by en-Gal4. Proliferating cells are marked by PH3. **(B, B')** Samples expressing constitutively active insulin receptor *InsR^{CA}* in the posterior compartment driven by *en-Gal4*. The Gal4 driver is tagged to a GFP to label the compartment boundaries. Additional fluorescent labels have been included within the figure. **(C-i, ii, C-i', ii')** Cross section taken parallel to the DV axis of the pouch in the anterior and posterior pouch compartments, respectively. **(D)** Samples expressing constitutively active Myc in the dorsal compartment driven by *ap-Gal4* tagged with GFP to label the compartment boundaries. Additional fluorescent labels have been included within the figure. **(E-i, ii)** Cross-section taken parallel to DV axis of the pouch with the same fluorescent labels as C. Yellow arrow indicating increase in βPS intensity.

S2.12 Inhibition of *mTOR* reduces cell proliferation and inhibits actomyosin contractility resulting in a decrease in cell height and basal curvature. To test how proliferation impacts tissue geometry, we studied the effect of downregulating *mTOR*, a direct regulator of the cell cycle and cellular growth in the wing disc (Supplementary Fig. 14). First, we expressed the dominant negative form of *mTOR* in the posterior compartment with engrailed-Gal4. However, the progeny was lethal. Hence, we expressed *mTOR^{DN}* in the dorsal compartment of the wing disc with *ap-Gal4* (Supplementary Fig. 14A-ii). The expression of *mTOR^{DN}* in the dorsal compartment resulted in a compartment-specific reduction in basal curvature compared to the control (Supplementary Fig. 14B-i, ii, E-ii). We also report a decrease in cell height upon expression of *mTOR^{DN}* (Supplementary Fig. 14E-i).

Lastly, to study how inhibition of *mTOR* affects cytoskeletal regulation, we carried out IHC assays to measure spatiotemporal changes in βPS, pMyoII and Rho GTPases. Quantification and comparison of fluorescence intensities across both internal and global control revealed that *mTOR* inhibition does not cause any changes in βPS (Supplementary Fig. 14D-i). However, we report a statistically significant decrease in both Rho1 and pMyoII at the pouch basal surface (Supplementary Fig. 14D-ii). Lastly, we also analyzed the AP cross sections within genetically perturbed *ap*> *mTOR ^{DN}* samples in the dorsal and ventral compartments of the wing imaginal disc (Supplementary Fig. 14Fi-iii). A loss of *mTOR* in the dorsal compartment led to qualitatively reduced basal curvature along the AP axis.

This study reveals that apart from a reduction in cell proliferation, *mTOR* inhibition also reduced both basal pMyoII and Rho1. It is noteworthy to mention that basal pMyoII is a key component of interkinetic nuclear migration (IKNM), a process that is required for cell proliferation within pseudostratified epithelia like wing discs. It is quite possible that a reduction in cell proliferation is a by-product of reduction in basal actomyosin contractility inhibiting IKNM.

To corroborate our experimental results, we used our computational model to run additional simulations where we decreased both cell proliferation and basal contractility in the posterior half of the *in silico* pouch (Supplementary Fig. 15C, D). These simulations qualitatively capture the loss in tissue folding (i.e. tissue flattening) observed within the *en*>*InsR^{DN}* (Figure 7) and *ap*>*mTOR^{DN}* samples.

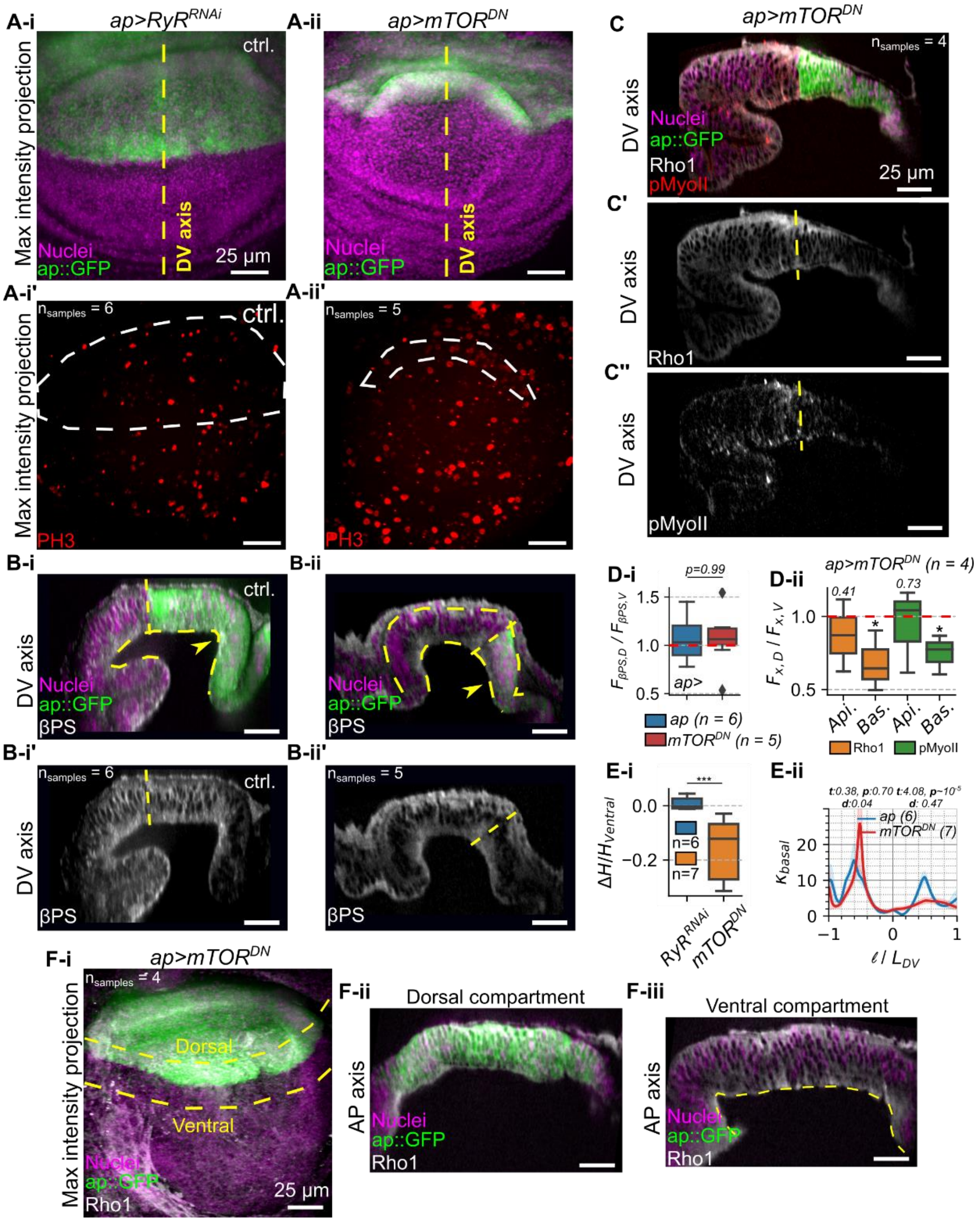

Supplementary Figure 14. (A) Maximum intensity projection of **(i-i')** control and **(ii-ii')** samples expressing dominant negative *mTOR*, $mTOR^{DN}$ in the dorsal compartment using ap::GFP. **(B)** Cross-section along DV-axis for (i) control and (ii) $mTOR^{DN}$. **(C-C")** Immuno staining of Rho1 and pMyoII for *ap>* $mTOR^{DN}$ genetic perturbations **(D)** Quantification of ratio of βPS across the dorsal to ventral compartment for discs expressing $mTOR^{DN}$ (*ap>* $mTOR^{DN}$) in the wing disc dorsal compartment. Similar analysis for (ii) Rho1 and pMyoII across pouch apical and basal surface. **(E-i)** Box plot visualizing the differences in average cell heights between dorsal and ventral compartments of the wing disc for *ap>*RyR^{RNAi} and *ap>* $mTOR^{DN}$

genetic perturbations (ΔH). ΔH was normalized by the average height of the tissue in ventral compartment (internal control). **(E-ii)** Plots quantifying the basal curvature profile for the DV pouch section of *ap>RyR*^{RNAi} and *ap> mTOR*^{DN}. Solid color indicates the mean while the shaded region shows the standard error of mean. The t-statistic (*t*), p-value (*p*) and effect size (*d*) for comparison of mean curvature profiles of control and genetically perturbed samples in dorsal and ventral compartments have been indicated on the top of plot. **(F)** AP cross sections of **(i)** *ap> mTOR*^{DN} mutant taken in the **(ii)** dorsal and **(iii)** ventral compartments of the wing imaginal disc.

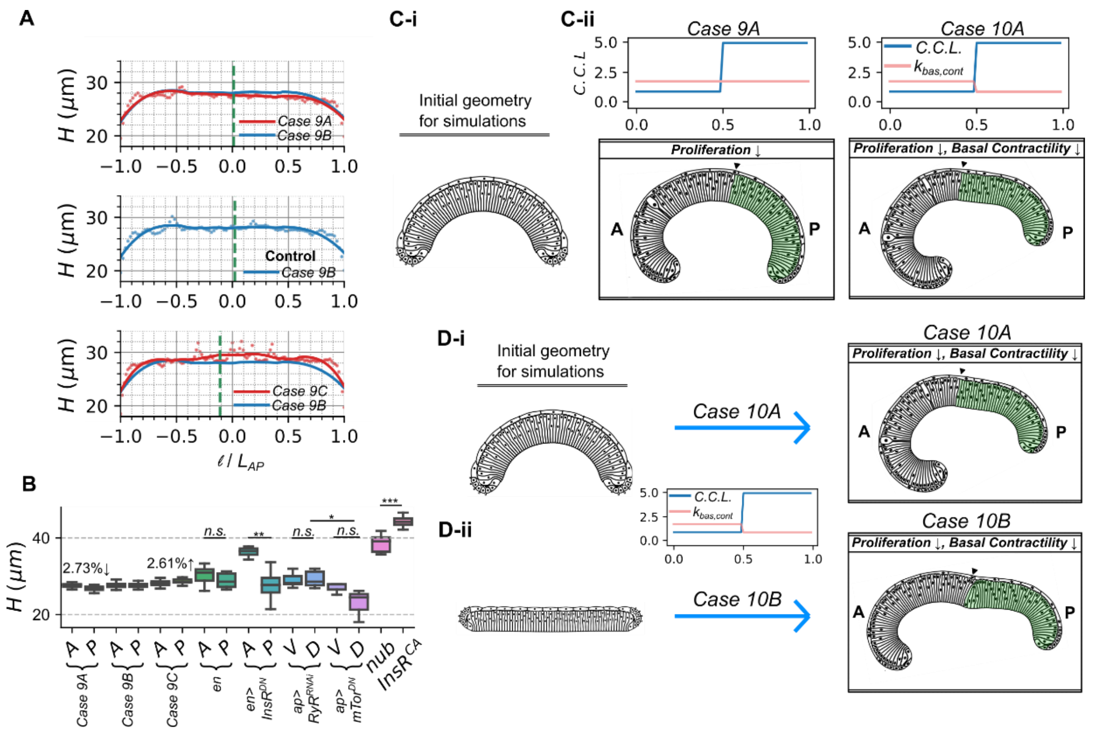

Supplementary Figure 15. (A) Quantification of cell heights for the Simulation Cases 9A-C presented within Fig. 6 of the main manuscript. **(B)** Box plot visualizing the cell heights within the control (anterior~en or ventral~ap) and perturbation (posterior~en or dorsal~ap) compartments of simulations and experimental data presented within Fig. 6 of the main manuscript. **(C) (i)** Initial geometry used for Cases 9A and 10A. **(ii)** Simulation cross sections for cases where cell proliferation was decreased in the posterior compartment. In addition to decreased cell proliferation, basal contractility in the posterior side was also decreased for Case 10A. **(D)** Simulation cross sections in which both cell proliferation and basal contractility were decreased in the posterior compartment. The two cases (Case 10A and 10B) represent shapes arising from different initial geometries (curved or flat) used.

S2.13 Inhibition of *mTOR* does not affect the tissue geometry during the initial stages of development. To investigate morphological data at 72 h AEL for the perturbations reported in the later half of the manuscript, we collected wing discs (n=12) from apterous>white as a control and apterous>*mTOR*^{RNAi} perturbation at 84 ± 1.5 h AEL (Supplementary Fig. 16 A-A') and stained

the discs against βPS (Integrin) and pMyoII antibody (Supplementary Fig. 16 B-B', C-C'). Qualitatively, we find that the shape of the tissue remains consistent between the internal control (Supplementary Fig. 16 A-A'), non-perturbed ventral compartment and the genetically perturbed dorsal compartment (Supplementary Fig. 16 B-C'). We did not observe significant changes in cell height between the dorsal and ventral compartments, indicating an absence of significant early tissue morphological changes. Hence, we can conclude that our simplifying assumptions are appropriate in the current investigation.

We also investigated wing discs at 72 h (figures not shown), but there is apterous expression on the ventral side at 72 h, which should be contained in the dorsal side. This signifies that the D-V boundary is not fully formed at this stage, and we are likely not yet perturbing the gene in the desired spatial pattern at such an early stage. Hence, we proceeded with our experimentation at 84 h AEL. Future analysis of all genetic perturbations accounting for the possibilities of developmental delays and maturation with early-stage wing discs may provide interesting insights but is beyond the scope of the present investigation.

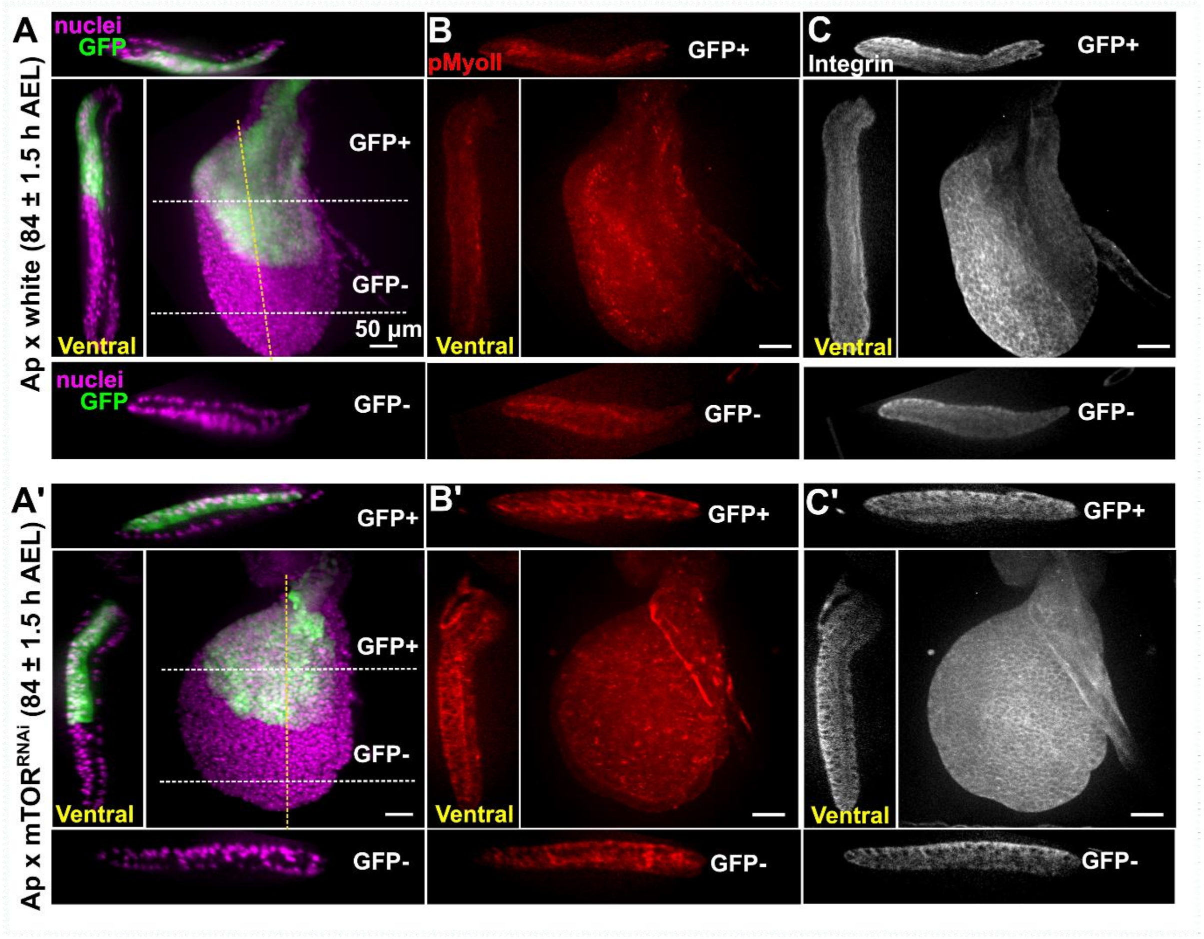

Supplementary Figure 16. The shape of the tissue remains consistent between the control, non-perturbed ventral compartment and the genetically perturbed dorsal compartment at very early stages of development. Wing discs (n=12) from **(A, B, C)** apterous>white (control), and **(A', B', C')** apterous>*mTOR*^{RNAi} at 84 ± 1.5 hours AEL and stained the discs against **(B, B')** pMyoII and **(C, C')** βPS (Integrin) antibody to observe the changes in cell heights.

S2.14 Inhibiting Dpp signaling activity decreases Rho1 expression and reduces inwards bending at the pouch lateral domains. The GAL4/UAS[2] system was used to downregulate the activity of Dpp signaling in the posterior compartment of the wing disc with a cross between an engrailed-Gal4 driver and a commercially available *UAS-*Tkv^{RNAi} line, and AP cross sections of the discs were analyzed (Supplementary Fig. 17B). An anti-PMAD antibody staining was carried out for validation. Expression of Tkv^{RNAi} leads to a reduction in fluorescence intensity of PMAD signals in the posterior half compared to the anterior half (Supplementary Fig. 17B'). Comparing this with the control, a wing imaginal disc for the parental engrailed-Gal4 driver, the fluorescence peaks in both the anterior and posterior half are roughly comparable (Supplementary Fig. 17A'). Next, an anti-Rho1 antibody staining was carried out to measure changes in cytoskeletal regulation (Supplementary Fig. 17B''). Rho1 expression is known to promote the phosphorylation of myosin and, hence, is a regulator of tissue contractility[10]. We report a decrease in the expression of Rho1 in the posterior Tkv^{RNAi} expressing compartment compared to its control anterior half (Supplementary Fig. 17B''). On the other hand, the expression of Rho1 for the control is symmetric for the anterior and posterior halves (Supplementary Fig. 17A''). Lastly, we also report a loss in inward bending at the lateral end of the epithelium in the posterior half, where the Tkv receptors were inhibited (Supplementary Fig. 17B).

To validate the Tkv^{CA} genetic perturbations (Fig. 7), we first expressed Tkv^{CA} only in the posterior compartment of the wing imaginal disc using an *en-Gal4* driver (Supplementary Fig. 17C). A PMAD antibody staining was carried out to validate the mutations. PMAD is downstream of Tkv and expression of constitutively active form of Tkv receptors should increase PMAD expression. As expected, we report an increase in PMAD fluorescence in the wing disc posterior compartment (Supplementary Fig. 17C') where Tkv^{CA} was expressed. In the Tkv^{CA} genetic perturbations, we also observed a non-compartment specific upregulation of Rho1 (Supplementary Fig. 17D-D', F-F') and pMyoII (Supplementary Fig. 17E-E'). We also report the distribution of average cell heights in the anterior and posterior compartments for *en>*Tkv^{CA} and *en>Myc* genetic perturbations. A student t-test was used to compare the statistical significance between population means (***:$p<0.001$, **:$0.001<p<=0.01$, *:$0.01<p<0.05$).

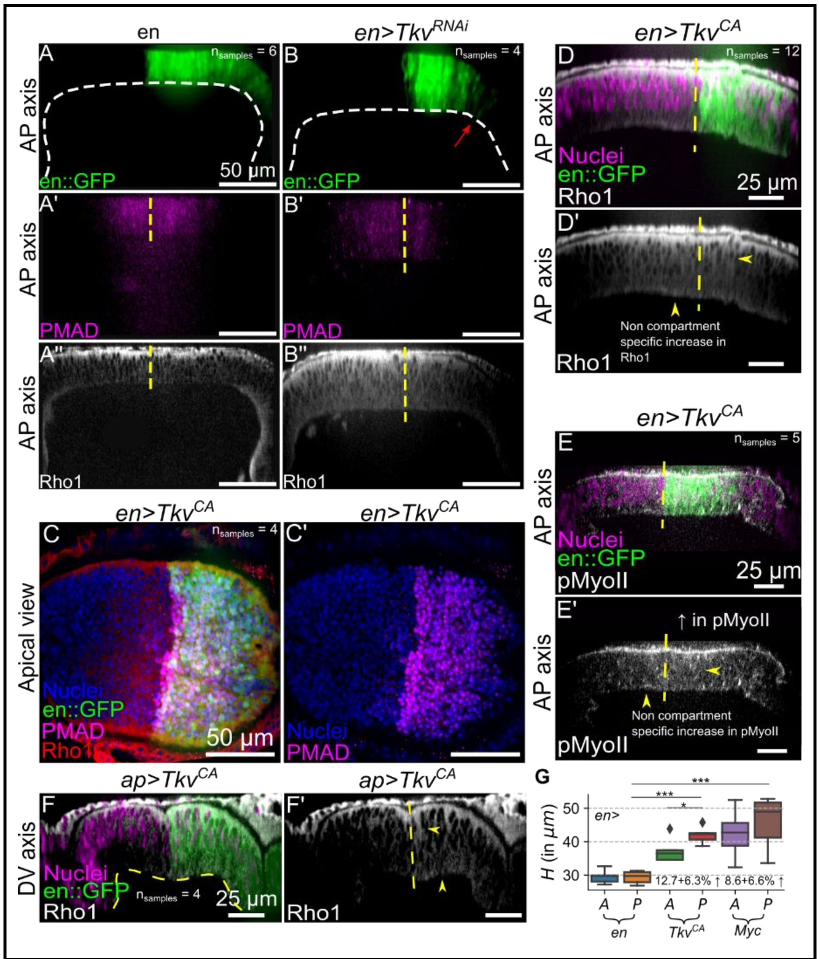

Supplementary Figure 17. Inhibition of Tkv receptors results in a reduction of Rho1 followed by a loss in inwards bending of lateral pouch domains. (A-A'') Cross-section along the DV boundary of a control wing imaginal disc dissected from larvae of an *engrailed-Gal4* driver. Fluorescence labels have been indicated in the lower left panel of each figure. **(B-B'')** Cross-section along the DV boundary of a wing imaginal disc expressing Tkv^{RNAi} in the posterior compartment. The posterior compartment also expresses GFP as indicated in A and B. (**C-C''**) Apical view of discs expressing Tkv^{CA} in the posterior compartment of the wing disc. A PMAD antibody staining was carried out to validate the expression of Tkv^{CA}. (**D-E)** Cross-section along the DV boundary of a wing imaginal disc expressing Tkv^{CA} in the posterior compartment and stained against **(D-D')** Rho1, **(E-E')** pMyoII. **(F-F')** Cross-section along the AP boundary of a wing imaginal disc expressing Tkv^{CA} in the posterior compartment and stained against Rho1. The posterior compartment also expresses GFP as indicated in A and B. **(G)** Box plot visualizing the cell heights within the control and perturbation compartments of experimental data.

S2.15 Overexpression of Myc causes a reduction in pMyoII and Rho1 fluorescence peaks along the AP axis. An *engrailed-Gal4* driver was used to overexpress Myc in the posterior compartment of the wing imaginal disc. An IHC assay was carried out to quantify the spatial expression of Rho1 (Supplementary Fig. 18A, A') and pMyoII (Supplementary Fig. 18A, A") along the pouch AP axis. Quantifications reveal that, unlike the anterior half, the posterior half of the wing imaginal disc does not have the peak in fluorescence near the folds, as shown in Supplementary Fig. 18B, B'.

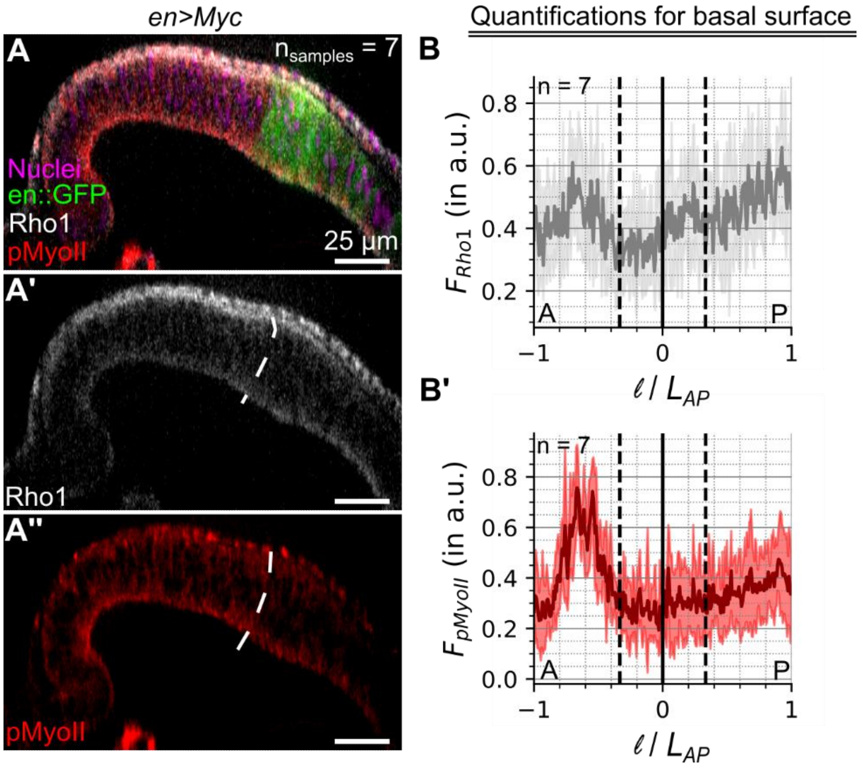

Supplementary Figure 18. Overexpression of Myc reduced pMyoII in the lateral domain and reduced basal curvature. An *engrailed-Gal4* driver was used to overexpress Myc in the posterior compartment of the wing imaginal disc. **(A-A")** AP cross-sections of the pouch were visualized. The fluorescent labels have been indicated as insets in the left bottom corners of each plot. A solid white line in A'-A" indicates the compartment boundary separating the anterior (left) and posterior (right) compartments. **(B-B')** Quantification of Rho and pMyoII across the pouch basal surface. Solid lines indicate the mean, while dashed lines indicate the standard deviation in prediction. Sample sizes have been indicated as the top-left inset of plots.

S2.16 Increasing proliferation beyond the biological limits causes severe morphological changes in the shape of the simulated wing imaginal disc. To understand the role of patterned proliferation in regulating wing disc shape, we decreased the cell cycle length (C.C.L.) of the cell in the medial domain of the pouch to increase proliferation in the pouch central region compared to its lateral counterparts. We increased proliferation in the medial region by 1.4, 2, 10 and 20

times the control proliferation rates, whereas the proliferation rates for the lateral regions of the pouch were kept intact in each case (Supplementary Fig. 19A i-iv). It should be noted that cell proliferation rates are patterned across the pouch AP axis at earlier developmental stages. The central region proliferates nearly 1.4 times more than the lateral region[8].

The proliferation rates decrease with the age of the pouch. Cases II, III and IV were run to test the effect of very high proliferation rates. With increased proliferation, the cells in the medial domain start to deform, with an increase in variability of cell height across both the medial and lateral pouch domains (Supplementary Fig. 19Bi, ii). Interestingly, some of the cells appear to be pushed by their neighbors toward the basal pouch surface (Supplementary Fig. 19Aiii, iv). We also see strong deformations on the pouch basal surface where the basal surface extrudes outward locally. This is a current limitation of our computational model, where we do not model cell death due to overcrowding. It has been shown across other model systems that additional pressure exerted by neighboring cells due to overcrowding can initiate cell death pathways[11–14], often as a result of mechanosensation[15], causing the cell to extrude out of the tissue. Very interestingly, due to instability arising because of this overcrowding, we see nuclei within the model simulations going out of the cell, suggesting that the forces within the tissue are no longer able to hold its position within the tissue. Our results here show how a biologically accurate model, when pushed to its limitations, can result in additional novel biological insights, requiring future experimental validations.

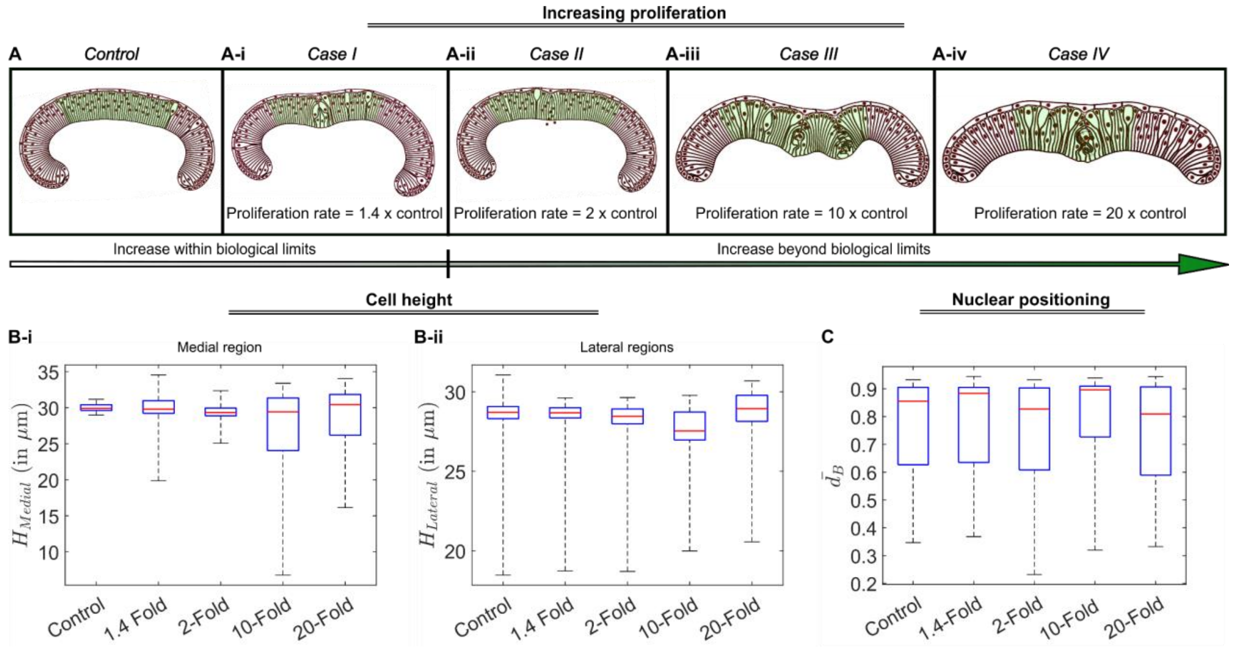

Supplementary Figure 19. Increasing proliferation rate beyond biological limits in silico (A-A iv) Through a series of cases, model parameters were varied across the AP axis with proliferation being increased 1.4, 2, 10, and 20 times the control in the central region of the pouch (shown in green). Case II through Case IV correspond to increasing proliferation beyond biological limits. **(Bi-Bii)** Box plot representing cell heights at the (B) medial and (B') lateral regions corresponding to the cases in A-A iv. **(C)** Boxplot quantifying the nuclear position corresponding to cases in A-A iv.

S2.17 Loss of cell-ECM adhesion causes apical constriction within the tissue. Analysis of βPS genetic perturbations showed severe changes in basal curvature, cell height (Fig. 4B, C, D), and nuclear positioning (Fig. 5D, D') within the tissue. To better understand the shape changes occurring because of inhibiting βPS, we used our computational model to first decrease the cell-ECM adhesion by reducing the model parameter k_{adhB} in the pouch medial domain to mimic the knock down of βPS. We additionally allowed the tissue to grow starting with a flat disc of 86 cells with a constant C.C.L. across the AP axis. With a decrease in k_{adhB} , the tissue starts to form an apical constriction (Supplementary Fig. 20A-ii) similar to what we report in our experimental data (Supplementary Fig. 20B). It is known that cell-ECM adhesion is critical for maintaining the basal surface tension of the cell and that a dysregulation between apical, basal, and lateral tension within pseudostratified epithelium can generate folds. Through this case study, we show how our biophysical growth model can accurately model the experimentally observed tissue shape.

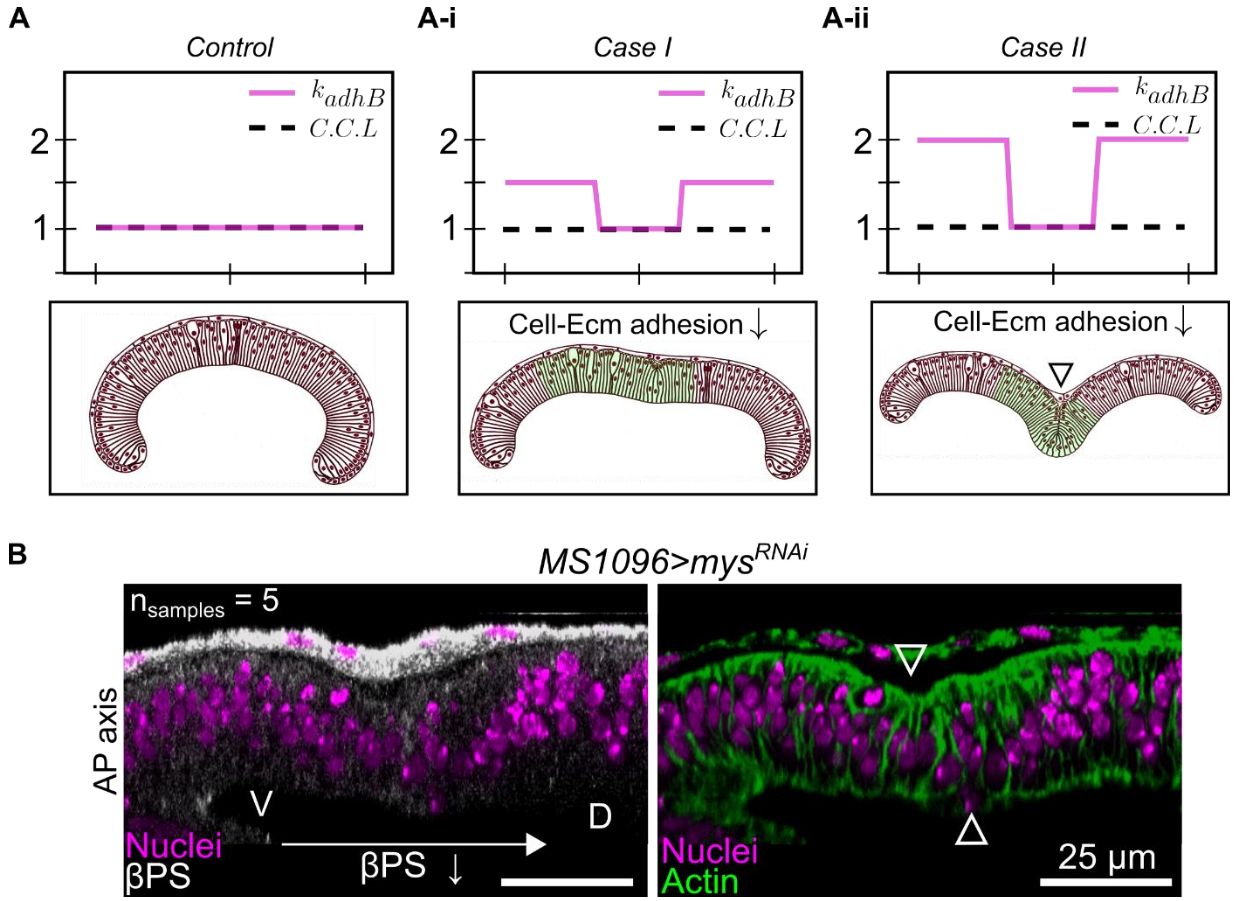

Supplementary Figure 20. Decreasing cell-ECM adhesion with uniform proliferation induces an apical constriction in the medial region of the pouch (A-Aii) Model outputs for simulated cases where proliferation was uniform across the tissue (C.C.L.), but cell-ECM adhesion (k_{adhB}) was patterned. In particular, k_{adhB} was decreased in the medial region by 50% and 100% in Case I and Case II, respectively. **(B)** MS1096-Gal4 driver was used to express mys^{RNAi} in the dorsal compartment of the wing imaginal disc. An optical section along the AP axis has been visualized. Fluorescent labels have been indicated as an inset within the plot. A decrease in βPS leads to basal nuclei migration (left panel) and an apical constriction formation near the central region (right panel).

S2.18 Expression levels of Rho1 quantified using Rho1 Biosensor ANI.RBD-EGFP and Rho1 antibody correlate. We requested and received the ani:RBD[16] reporter fly line developed by Dr. Thomas Lecuit's lab from Dr. Lynn Cooley. We fixed the wing disc from this fly line at 6 days AEL and stained it against the Rho1 antibody used throughout the manuscript. The GFP expression of the Rho1 biosensor qualitatively appears to have positive colocalization with the antibody expression (Supplementary Fig. 21). We also tested a commercially available fly line with Rho1/Rac GTPase activity biosensor (BDSC #52298), and this also showed qualitatively the same expression as the Rho1 antibody accumulation (results not shown). Hence, we conclude that antibody staining of fixed images are roughly equivalent in terms of patterning for both the internal biosensor and antibody staining of fixed tissues.

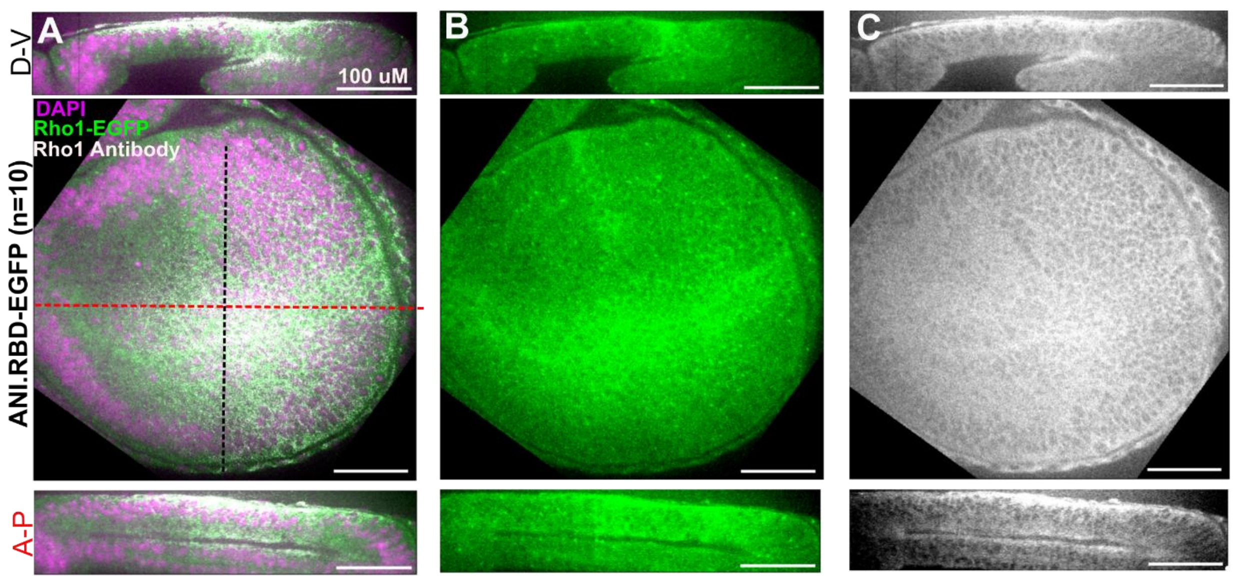

Supplementary Figure 21. Comparison of Rho1 expressions as visualized using a biosensor and an antibody. (A) Composite, **(B)** ANI.RBD-EGFP Rho1 Biosensor, and **(C)** anti-Rho1 antibody staining to observe the expression of Rho1 in the same wing disc. The GFP expression of the Rho1 biosensor (panel B) qualitatively appears to have positive colocalization with the antibody expression (panel C).

S3. Computational modeling methods

S3.1 Overview of computational modeling approaches. Multiple computational modeling approaches varying from continuum to discrete cell-based models have been used to study epithelial tissue mechanics and morphogenesis[17]. For example, in Keller et al., a continuum model employing the finite element method was developed to reproduce deformation experiments on the *Drosophila* wing imaginal disc to investigate how geometry affects its elastic properties[18]. In Levis et al., a compression microfluidic device was developed to compress the wing imaginal disc, and a continuum computational model was used to estimate the elastic modulus of the disc based on the deformations occurring in the wing disc[19]. In particular, Levis et al. used a finite element

model using the structural mechanics module of COMSOL[20] to simulate the wing disc cross section and show that the elasticity of discs increases with age. Recently, Harmansa et al. developed a continuum three-dimensional finite element model to study the bending of the *Drosophila* wing imaginal disc[21] to demonstrate that the domed shape of the wing disc can be driven by the differential growth rate between the columnar cells and their associated basal ECM and not by the difference in growth rates between the columnar and peripodial tissue layers. In general, continuum modeling approaches are more appropriate to capture macroscale tissue-level phenomena and cumulative tissue mechanisms as they usually do not model cell-level properties and biological processes such as individual cell adhesion, division, and intracellular processes.

Unlike continuum models, discrete cell-based models simulate cell-level processes, such as cell-cell adhesion and individual cell division. Cell-based models can be categorized into either lattice or off-lattice modeling approaches (see Osborne et al.[22] and Honda and Nagai[23] for extended reviews). A lattice-based model such as the Cellular Potts model (CPM) represents a cell as a cluster of lattice sites that interact with one another via Monte Carlo rules based on changes in energy potentials. Among other biological problems, CPMs have been used to study morphogenesis, limb formation, cell aggregation, and cell sorting[24–29]. These types of models can efficiently capture a great number of cells and they are easy to implement computationally. In terms of off-lattice models, a well-established modeling approach is the vertex-based model, which represents cells as polygons composed of vertices connected by edges where each vertex moves under the action of the force term acting on it[30]. In Nagai and Honda[31], the vertex model was applied to study wound closure in epithelia and demonstrated that cell basal-lamina adhesion is responsible for proper closure. In other applications, the vertex-based modeling framework was applied to study cell shape formation, tissue morphogenesis, and regulation of tissue size[30,32–38]. For an extensive review of the vertex models and various applications, please see the book by Honda and Nagai[39].

Several previous computational models have been developed to study the impact of changing mechanical properties of epithelial tissue on its structure and shape[40–42]. A common setup in previous models is to describe the epithelial layer as a tightly connected two- or three-dimensional structure composed of polyhedrons. As surveyed by Smallwood, earlier models can be traced back to the paper by Honda that describes the epithelial structure from a top-down view using Dirichlet domains[40,43]. Subsequent models, either two-dimensional or three-dimensional, were developed utilizing geometrical techniques represented by topology dynamics, center dynamics, boundary dynamics, or vertex-based dynamics where an individual cell is depicted as a polyhedron[37,39,44–50].

Physics-based models describing an epithelial cell layer where mechanical stress and elastic deformation are modeled via potential terms, stress-tensor, or continuum approach, are a key area of continued interest[40,51–54]. These computational models have been used to provide insight into important biological questions including the formation of epithelial folding and invagination to identify potential mechanisms driving such processes. For instance, in the work of Marin-Riera et al., an off-lattice center dynamics model was developed to illustrate the ability to capture the

interaction between an epithelial layer with its surrounding extracellular matrix (ECM), and how differential adhesion, cell migration, and cell contraction lead to tissue deformation[55]. A key aspect of these models is allowing different degrees of contraction at the apical and basal surface, regardless of the model dimension, to achieve deformation. However, such models often make simplified assumptions including but not limited to reducing the contacting surfaces between two neighboring cells into a single surface, and therefore reducing the flexibility of the lateral cell surfaces[56,57]. In addition, it is also frequently assumed that the contraction is surface-bound, either apically or basally. For a detailed review of physical models developed to study folding and invagination, please see Rauzi et al[44].

Recently, Tozluoglu et al. developed a 3-dimensional finite element model where each individual cell in the *Drosophila* wing pouch is represented with triangular prisms, and growth is modeled by the increase in the volume of prisms, to study the mechanism for fold formation[58]. This model captures the ability of cells to resist deformation including shear strain. They conclude that external resistance to growth from the ECM is essential for buckling the tissue and the increased apical stiffness can induce the correct number of folds. Ioannou et al. developed a three-dimensional hybrid vertex model that allows a more complex polyhedron representation of the epithelial cells, improving the ability of capturing the packing of cells more realistically[59].

There have also been models developed to identify the links between mechanical behaviors with the underlying chemical signaling pathways. The work by Hughes et al. has delved into how mechanical compaction of the extracellular matrix during mesenchymal condensation leads to tissue folding[60]. In the work of Zmurchok et al., a two-way feedback between signaling and mechanical tension was investigated to observe waves of contraction and relaxation sweeping through a two-dimensional model epithelium[61]. However, it still remains unclear how morphogenesis arises from the interplay between mechanical contraction and signaling networks. To fill this gap of knowledge, we specifically explore the relative contributions of cell proliferation and actomyosin contractility in regulating tissue curvature, cell height, and nuclear positioning.

In this paper, we use the general Subcellular Element (SCE) modeling approach, which was initially developed by Newman et al[62]. Here, we developed a novel multi-scale computational model that allows us to study how changes in local subcellular mechanical properties and cell proliferation regulate the dynamical changes in epithelial tissue geometry. The SCE method provides unparalleled resolution in describing detailed intra- and inter-cellular interactions including cell-cell adhesion and membrane-nucleus interactions (Supplementary Fig. 22). Previously, SCE type models have been utilized to study both the top-down and cross-sectional view of the epithelial layer in the *Drosophila* wing disc[46,47]. While more computationally intensive, the SCE approach represents an interplay between cellular deformation due to contraction and the dynamics of subcellular components including the nucleus, which is often absent in more coarse-grained models. To overcome the computational cost, most models using the SCE approach have been parallelized on GPU computer clusters. One final and key advantage of the SCE model is that the cell mechanical properties can be directly calibrated based on experimental data[46,63].

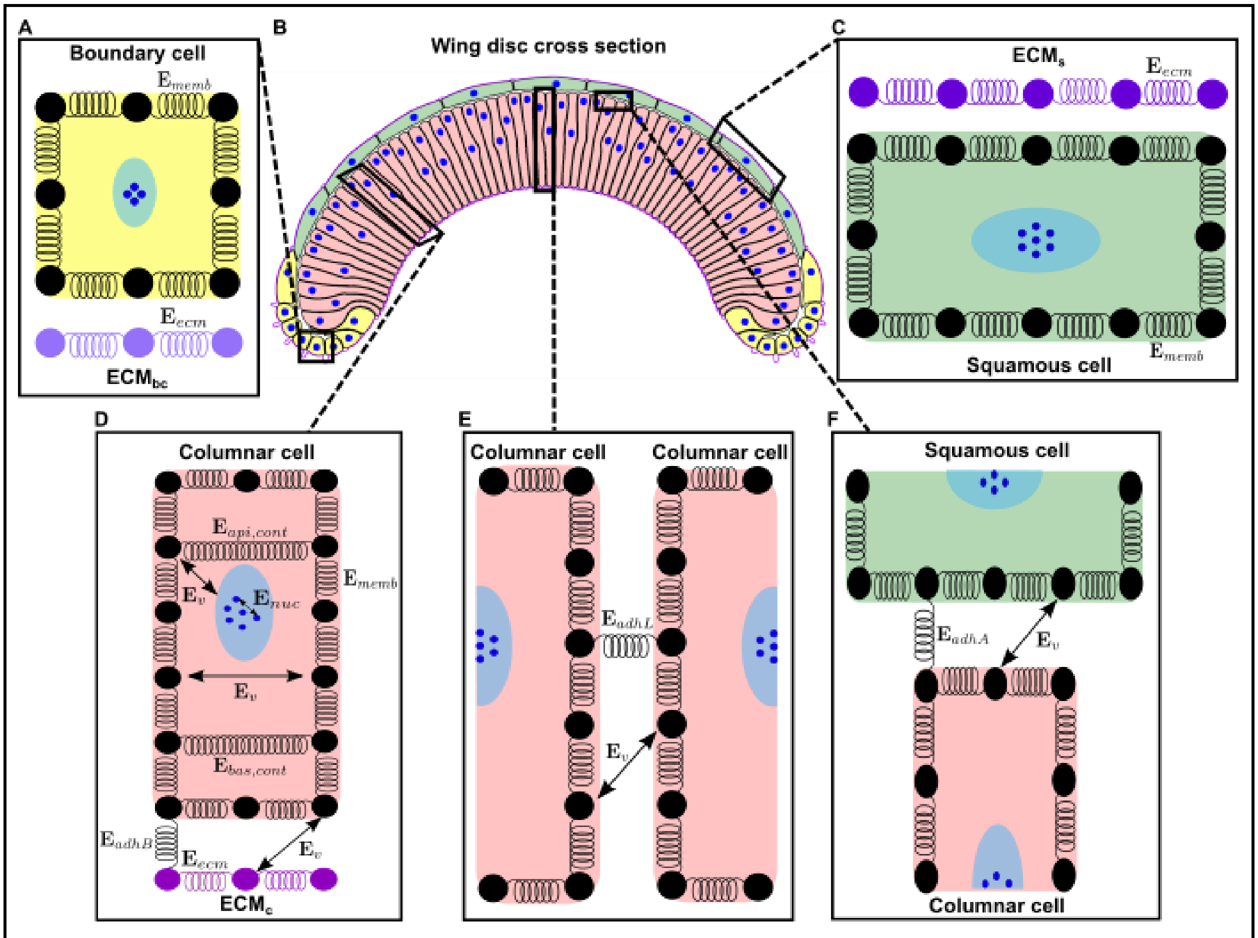

Supplementary Figure 22. Two-dimensional (2D) multi-scale subcellular element (SCE) model of the *Drosophila* wing disc cross section along the anterior-posterior (AP) axis. This model closely resembles the wing disc cross-section by including a representation of the three different cell types (boundary, squamous and columnar cells) that make up the cross-section and of the ECM, which connects to the basal surface of the cells. Simulated cells, nuclei, and ECM are represented by a set of nodes interacting via different potential energy functions. **(A)** Model representation of a boundary cell and its associated ECM denoted by ECM_{bc}. **(B)** Model simulation of the cross-sectional profile of the wing disc along the AP axis includes boundary cells (colored in yellow), columnar cells (colored in light red), squamous cells (colored in green), and the ECM (colored in purple). **(C)** Model representation of a squamous cell and its respective ECM denoted by ECM_s. **(D)** Model representation of a columnar cell with different potential energy functions that capture intracellular interactions, apical and basal actomyosin contractility and cell interactions with the ECM (denoted by ECM_c). Although we only use a single spring to represent the apical ($E_{api,cont}$) and basal ($E_{bas,cont}$) contractility in (D), in the model there are actually several apical and basal contractile springs. See section S-3.3 for details. **(E)** Adjacent columnar cell nodes interact via linear spring E_{memb} and Morse potentials E_v to maintain the cells closely connected without the membranes overlapping. **(F)** The apical membrane of columnar cells is connected to squamous cells.

S-3.2 Initial conditions for the computational model. Starting with the same initial tissue configuration, the quasi-steady states of perturbed model tissues are compared with the reference model tissue presented in the previous publication[47]. Comparisons were drawn at 50,000 AU

(arbitrary unit) in simulation time, where each simulation time step size is 0.002 AU similar to the previous publication[47]. The midsection of the tissue can maintain its relative flatness via balancing the apical and basal contractility (Fig. 3E-i). Furthermore, the initial condition for simulations with cell proliferation is a curved shape representing the 72-hour AEL mark of the wing disc development (Fig. 2B-i). The default value for the spring coefficients of both apical and basal contractile springs is set as $9.0\ \mu N/\mu m$.

S- 3.3 Determination of the number of springs in non-mitotic cells. The number of contractile springs within a non-mitotic cell depends on the height of the cell (H_{cell}) and the local actin intensities. To determine the number of springs, we first need to determine the apical and basal portions of a cell that can be occupied by springs. Measuring from the apical side of a cell, the apical contractile spring height is denoted by ${H_0}^{api}$, which defines the upper region of the cell where apical springs can exist (Fig. 1C, right panel, apical light blue region). Similarly, measuring from the basal point of a cell, we have the basal contractile spring height ${H_0}^{basal}$ (Fig. 1C, right panel, basal dark blue region). The exact values of ${H_0}^{api}$ and ${H_0}^{basal}$ at the current iteration are determined by the local actin intensities. More specifically, we assume that ${H_0}^{api}, {H_0}^{basal} \leq 0.3H_{cell}$ and define ${H_0}^{api} = \alpha_i H_{cell}$ and ${H_0}^{basal} = \beta_i H_{cell}$. The weight constant α_i is defined by $\alpha_i = 0.3(A_i/A_{max})$ where A_i and A_{max} represent the local apical actin intensities and the maximum apical actin intensity, respectively. The calculation for β_i is similar to α_i, except we utilize the local and maximum basal actin intensities. Finally, a contractile spring becomes active if the distance between the apical point of the cell and the nodes connected by the contractile spring is less than ${H_0}^{api}$ or greater than $H_{cell} - {H_0}^{basal}$. If this distance is less than ${H_0}^{api}$, an apical contractile spring will manifest in the upper portion of the cell while if the distance is greater than $H_{cell} - {H_0}^{basal}$, then a basal contractile spring becomes active.

S-3.4 Modeling the extracellular matrix. The ECM is divided into three sections to represent the ECM associated with each cell type: the columnar cells (ECM_c), the boundary/cuboidal cells (ECM_{bc}) and the squamous cells (ECM_s) (Supplementary Fig. 22 A, C, D). The forces applied to the ECM are as follows:

$$F_{ecm,c} = k_{ecm,c}\,(L - L0_{ecm,c}) + c_1 \quad \textbf{(S1)}$$

$$F_{ecm,bc} = k_{ecm,bc}\,(L - L0_{ecm,bc}) + c_2 \quad \textbf{(S2)}$$

$$F_{ecm,s} = k_{ecm,s}\,(L - L0_{ecm,s}) \quad \textbf{(S3)}$$

The constants c_1 and c_2 in the equations (S1) and (S2) are added to represent the pre-strain of the basal ECM. This representation of prestrain is equivalent to the one used in Adam et al.[64]. The values of c_1 and c_2 were chosen based on obtaining simulated cell and tissue shapes that qualitatively reproduce the bent shape of the wing pouch as shown in experimental images (Supplementary Table 1). Previous biological studies also suggest that collagen fibers, key components of ECM in most of the organs, are under tension both prior to and during experiencing loads[65].

S-3.5 Model calibration pipeline. The computational model parameters have been calibrated using experimental data either from this study or from literature (Supplementary Table 1, 2). Experimental data related to the *Drosophila* wing disc cross-sectional profile from our previous work (Nematbakhsh and Levis, et al. 2020[47]) was used to calibrate the cell height, width and number of cells in the model. The model parameters associated with the ECM stiffness (E_{ecm}), volume conservation of the cytoplasm (E_{vol}) and columnar-squamous cell adhesion (E_{adhA}) were calibrated based on measurements found in literature[47]. The wild-type cell membrane stiffness k_{memb}, associated with the potential function E_{memb}, was calibrated in our previous study (Nematbakhsh et al 2017[46]) using the experimentally obtained modulus of elasticity of a single cell[49,50,66]. More specifically, a model simulation was conducted such that a single simulated cell was deformed by applying a linearly increasing force to the membrane nodes on either side of the cell. The deformation of the cell was calculated, and the cell elasticity was determined from the slope of the stress versus strain curve. The parameter k_{memb} was then chosen so that the modulus of elasticity remained within the experimentally measured range[49,50].

A previous study on epithelial cells by Sim et al. showed that the cell-cell and cell-ECM adhesion levels were within the same range[67]. Hence, the wild-type parameters associated with the cell-cell (E_{adhL}) and cell-ECM (E_{adhB}) adhesions were calibrated using experimentally determined cell-cell adhesion forces for epithelial cells[46]. A computational simulation was run where two adhered cells were pulled until detachment by applying equal stretching forces to the membrane nodes on both sides of these attached cells. Next, the force required to detach these cells was calculated. The parameter k_{adhL} was calibrated so that the force needed to rupture the cell-cell adhesion was in accordance with published experimental data[67,68]. k_{adhB} was then calibrated using data from literature[46,67].

In our previous work[47], the Latin hypercube sampling method[69] was used to perform a sensitivity analysis and obtain the range of values for the average nuclei diameter, ECM tension (E_{ECMc}, E_{ECMs}) and actomyosin basal contractility parameters ($k_{bas,cont}$). The derived parameter set was chosen such that it captured the experimentally measured global basal curvature, mean height and mean nuclei positioning the best. The apical and basal actomyosin contractility parameters ($k_{api,cont}$ and $k_{bas,cont}$) were calibrated using experimentally obtained data from this study. More specifically, $k_{api,cont}$ and $k_{bas,cont}$ were determined based on the local p-MyoII fluorescent intensity measured in experiments. The number of contractile springs in the model, which represented actin filaments inside individual cells, was calibrated using the experimentally observed actin intensity (see Supplementary text S-3.3). On the other hand, the membrane-membrane, membrane-nuclei and membrane-ECM volume exclusion (E_v) parameters were calibrated to ensure numerical stability.

The composite ECM spring constant (k_{ecm}) was calibrated based on the fact that the ECM is stiffer than pouch cells, as indicated in Keller et al.[18] and Harmansa et al.[21]. Specifically, k_{ecm} captures the experimentally observed local basal curvature, cell heights, and nuclear positions in Nematbakhsh and Levis et al.[47]. This study uses the same baseline parameter value. Because

the ECM of the pouch cells is stiffer than the pouch cells, we chose a larger value for the ECM spring constant than that of the pouch cell's membrane spring constant.

In this study, we also tested different model scenarios by perturbing parameters *in silico* (Supplementary Fig. 6) and used experimental data to calibrate these parameters (Figure 3-5, Supplementary Table 2) based on three metrics of calibration: (1) local basal curvature, (2) tissue thickness and (3) nuclear positioning (Figure 1B). Then, we determined the parameter values that generated results in agreement with the experimentally quantified metrics (1)-(3).

To capture cell growth and mitotic rounding, a dividing cell in the model undergoes time-dependent increments in cell volume and actomyosin contractile springs. Both of these increments were calibrated to ensure that cell growth and division completed in a timely manner consistent with experimental observation. Additionally, the maximum equilibrium cell volume (Ω_0^{max}) during cell growth was determined according to the expected cell volume prior to cell division. During mitotic rounding, an increase in the actin and myosin concentrations was experimentally observed towards the basal region of cells and represented in the model by a gradual increase in the number of basal contractile springs. The parameter h representing this increment in actomyosin per simulation time step was calibrated using experimental data. In particular, the experimentally determined time duration for the mitotic rounding process and the portion of the mitotic cell with high actomyosin intensity were used to determine h .

Finally, our computational model accounts for in-plane and out-of-plane cell division because during cell division, the new daughter cell can lie within or outside of the wing disc cross-section that we focused on in this study. The probability that a new daughter cell lies in the same cross-section was experimentally determined by calculating the frequency of daughter cells that remain in the same wing disc cross-section as mother cells.

S4. Image analysis and data quantification pipelines

S-4.1 Quantification of local basal curvature. OpenCV[70] in Python was used to get points along the basal surface as a user input (Supplementary Fig. 23A). The *splrep* function within Scipy's[71] interpolation module was used to fit a B-spline[72] curve to the points (Supplementary Figure 23B). Next, 90 equally distant points within the curve were sampled using the fit spline model. The sampled points were then scaled so that the total length of the curve was unity. Lastly the curve was centered around zero (Supplementary Fig. 23C-i). Using points on the normalized curve, a spline with a smoothness factor of 0.1 was fit to smoothen the curve (Supplementary Fig. 23C-ii). The *splev* function within Scipy's interpolation module was used to compute the first and second derivatives using the xy coordinates of the sampled points in order to compute the numerical curvature (Supplementary Fig. 23D) defined by formula below:

$$\kappa = \frac{\frac{dx}{dt}\frac{d^2y}{dt^2} - \frac{dy}{dt}\frac{d^2x}{dt^2}}{\left[\frac{dx}{dt}\frac{dx}{dt} + \frac{dy}{dt}\frac{dy}{dt}\right]^{1.5}}$$

For each case, we assume that the middle 30 points constitute the pouch medial domain while the other 30 points on either end belong to the lateral domains. This is based on the assumption where we define that the medial domain acquires the central third of the tissue at each developmental stage. A similar analysis was also carried out to quantify basal curvature for the cross sections generated during simulations (Supplementary Fig. 23 A'-D').

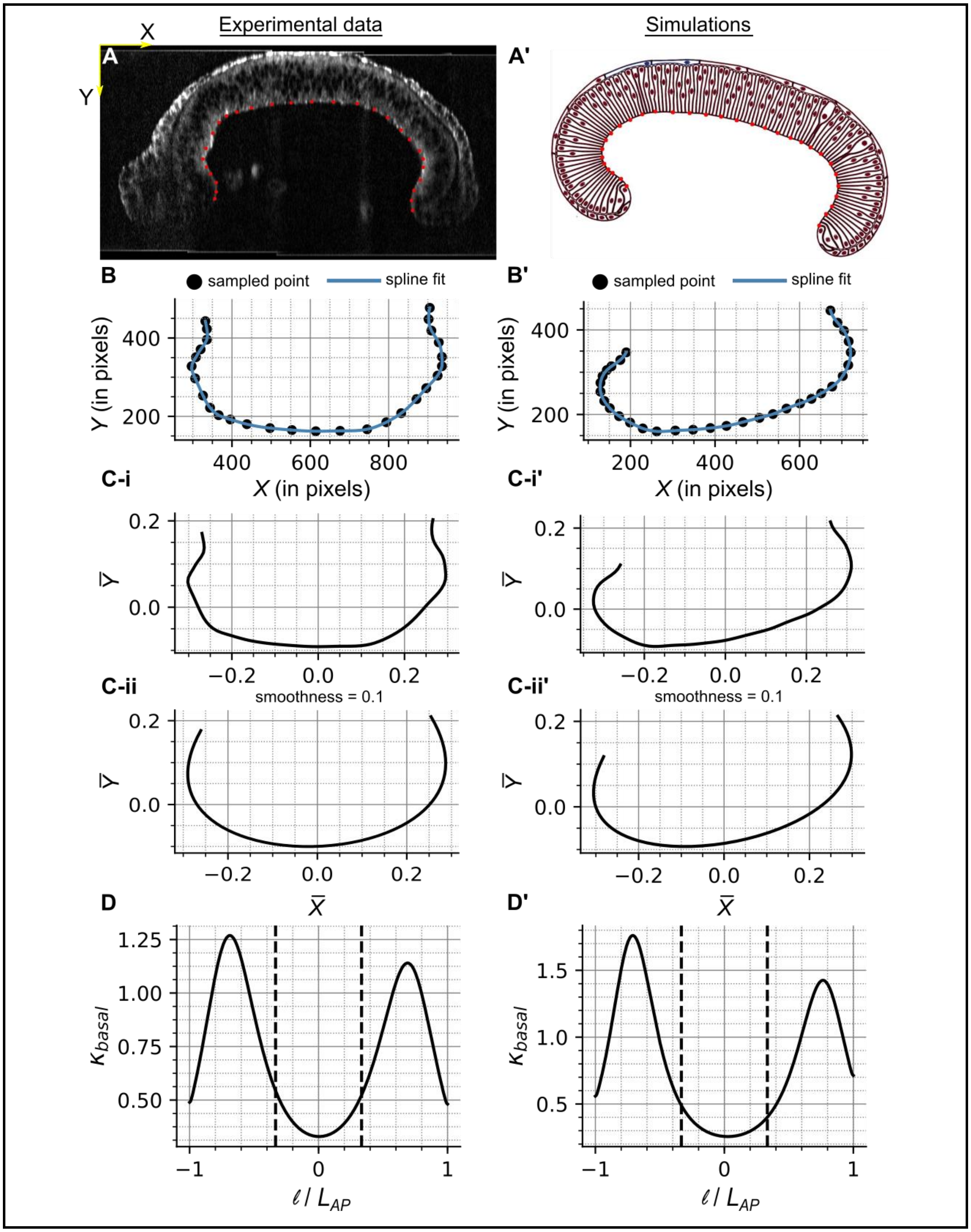

Supplementary Figure 23. Pipeline for quantifying local basal curvature (A) User-defined points along the basal surface is indicated in red over the raw cross section data. **(B)** Plot showing user defined points along the basal surface overlaid with a curve generated by fitting a spline. **(C)** Plot showing the normalized curve representing the basal surface. **(C-ii)** A spline with a smoothness factor of 0.1 is fit to smoothen the normalized curve**. (D)** Plot showing the variation of local basal curvature along the pouch AP axis. The x-axis represents the normalized distance along the basal surface where curvature was calculated. The normalization was done by dividing the distance by half the length of the basal surface. **(A'-D')** A similar analysis of basal curvature for simulation generated cross sections.

S-4.2 Quantification of morphological and signaling-related features from the wing imaginal discs. Using Kappa[73], an ImageJ[7] plugin, user-defined points were used to fit splines along the apical and basal surfaces, respectively. The tool was also used to extract fluorescent intensity of cytoskeletal regulators such as pMyoII along the points on the apical and basal surfaces of the columnar cells (Supplementary Fig. 24 A, B). Apical surface for the columnar epithelia is labeled carefully to avoid including points from the apical surface of the squamous cells.

In a custom MATLAB[1]-based tool, the number of discretization elements is first defined for analysis (N_{cells}). It is first used to split the basal curve into N_{cells} + 1 equidistant nodes. Using the discretized points in the basal surface, corresponding points on the apical surface area are then obtained in a way such that the distances between those points and the apical surface of the pouch are minimized. The methodology allows discretization of pouches into N_{cells} computational elements each mimicking a pouch cell (Supplementary Fig. 24C). A frustum-like shape obtained by joining any two consecutive points on the basal surface and the corresponding distance minimizing apical points is considered as a computational cell for our analysis. For any computational cell, the median intensity of all the points lying between the corresponding nodes on the apical and basal surface is used to approximate local pMyoII intensities across the apical and basal surfaces. Local height is defined as the average distance between the nodes on the basal surface and their counterparts on the apical surface. As an example, for a disc corresponding to 90 h AEL, the pipeline was used to quantify raw intensities of pMyoII in the apical and basal nodes (Supplementary Fig. 24D). The ratio of apical and basal pMyoII intensity across each computational cell (Supplementary Fig. 24E) along with the variation of local tissue thickness (Supplementary Fig. 24F) have also been plotted.

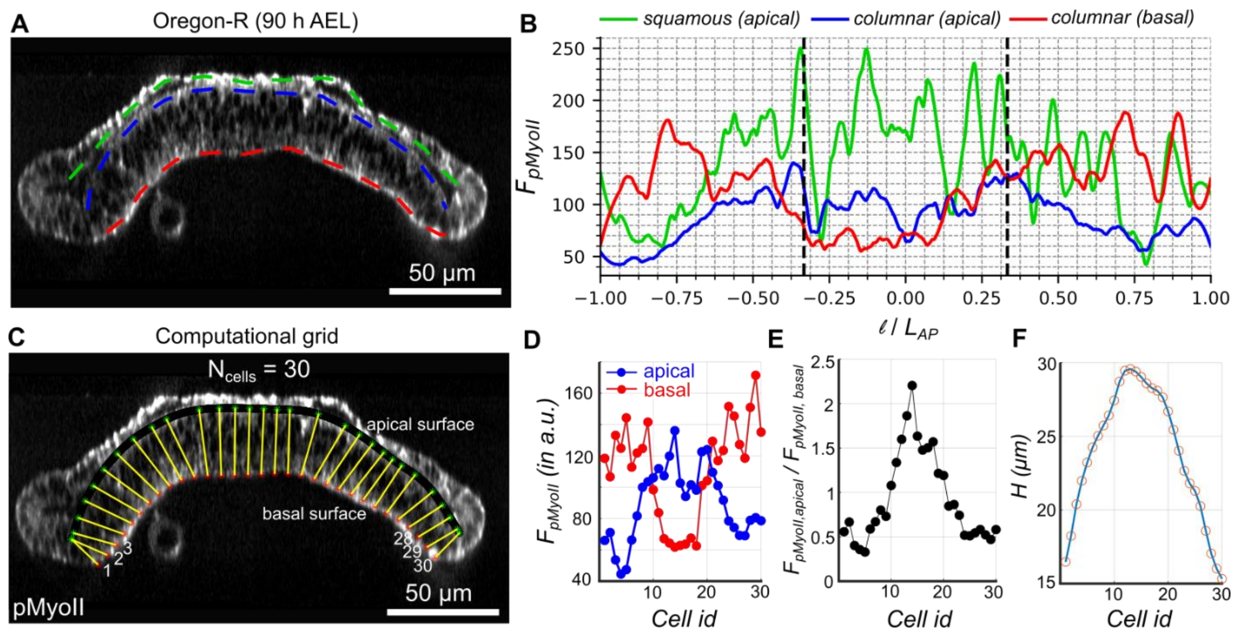

Supplementary Figure 24. Pipeline for extracting local tissue height and the apical-basal ratio of pMyoII. (A) Visualization of apical-basal surfaces for the columnar and apical surface for the squamous epithelia. **(B)** Plot showing fluorescence intensity of pMyoII at the pouch surfaces highlighted in A. **(C)** Visualization of 30 discretized computation cells mimicking columnar cells within the pouch. Cell ids have been indicated in the region below the basal surface of the pouch. **(D)** Plot showing fluorescence intensity of myosin at the apical and basal surfaces of the columnar cells within the pouch. **(E)** Plot showing ratio of apical to basal pMyoII for each computational cell within the pouch. **(F)** Plot showing the variation of local tissue height across the computational cells.

S-4.3 Quantification of nuclear positioning. StarDist[74], an open-source, deep learning-based ImageJ[7] plugin was used to segment nuclei from the background (Supplementary Fig. 25A, A'). The segmentation mask was imported to MATLAB[1] for further post-processing. MATLAB's image labeler was first used to generate a mask defining the pouch region to separate out the nuclei of squamous epithelia from the analysis (Supplementary Fig. 25B). Fluorescence visualizing the Actin cytoskeleton was used for this task. The same image was also used to get user input of points located on the apical and basal surface using MATLAB's *ginput()* function. Splines were next fit on the user-defined points to generate a smooth and continuous representation for the apical and basal surfaces (Supplementary Fig. 25B'). The *Regionprops* command within MATLAB was then used to estimate the centroid of each nucleus. Minimum distances between the centroid of the nuclei and the apical-basal surfaces were calculated using the *distance2curve* MATLAB function (Supplementary Fig. 25C, C'). The proximity of a nucleus from the basal surface is defined as the ratio of its distance from the basal surface over the sum of the distances from both the apical and basal surfaces. This quantity was used to generate a heatmap where each nucleus is color-coded with its proximity to the basal surface (Supplementary Fig. 25D). It can be clearly seen that as one moves towards the apical surface, the color transitions indicate an increase in the quantity.

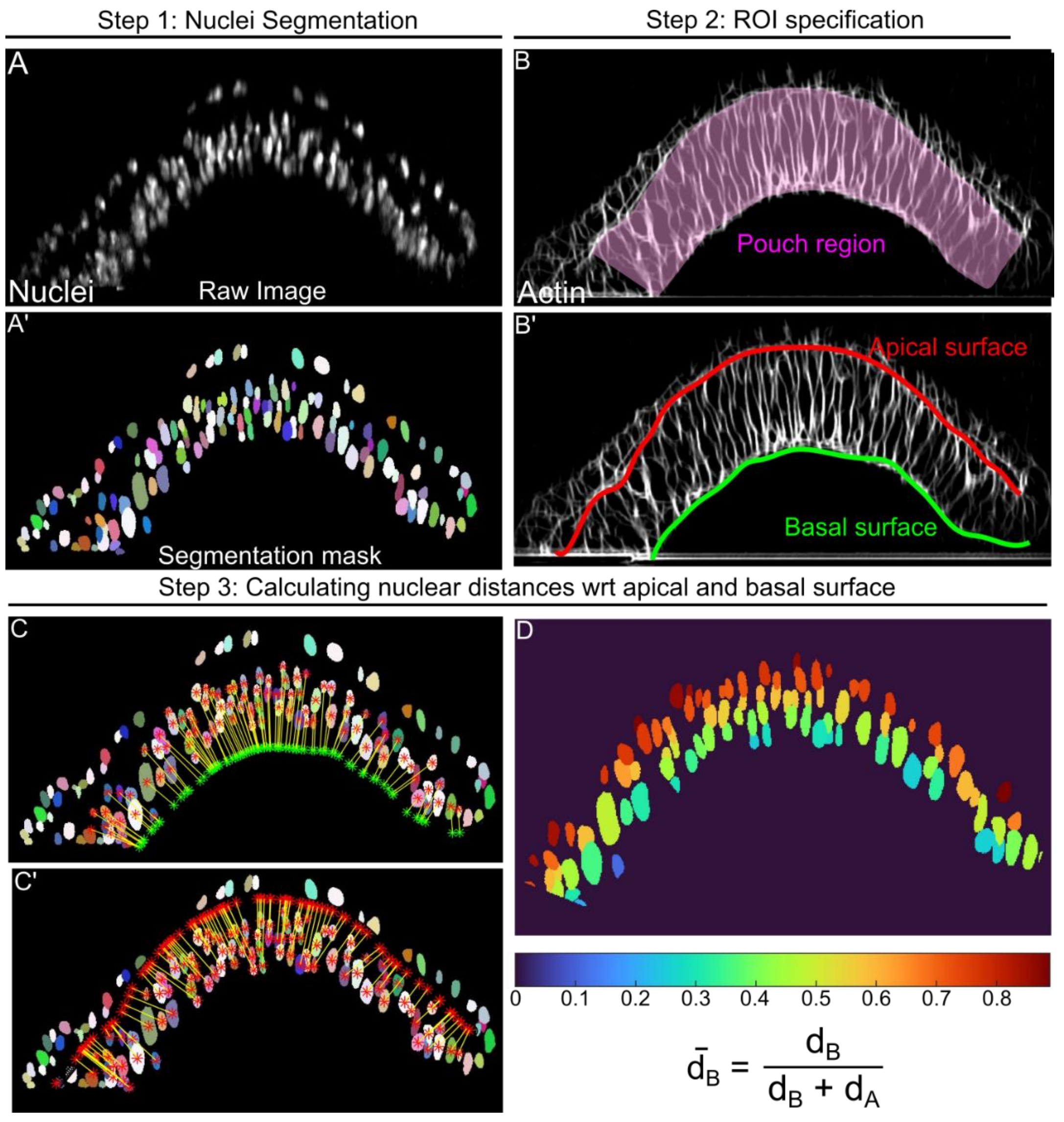

Supplementary Figure 25. Quantification of nuclear positioning. (A) DV cross-section of a 72 h AEL larval wing imaginal disc with fluorescence showing nuclei. **(A')** Segmentation mask generated using StarDist. Each color represents a different nucleus. **(B)** MATLAB's image labeler was used to define the pouch region using the fluorescence channel highlighting the actin cytoskeleton. **(B')** User-defined points on the apical and basal surfaces are used to generate splines. **(C, C')** Visualization of distances of the nuclear centroids from the apical and basal surfaces, respectively. **(D)** Heatmap visualizing relative positioning of nuclei with respect to the basal surface. It is defined as the distance of a nucleus from the basal surface (d_B) over the sum of distances of nuclei from the apical (d_A) and basal (d_B) surfaces.

S5. Experimental methods

S-5.1 Immunohistochemistry: Wing imaginal discs were dissected in phosphate-buffered saline (PBS) from 72 h, 84 h, 96 h, or 120 h AEL larvae in intervals of 20 min. Fixation was performed on dissected wing discs in ice-cold 4% paraformaldehyde solution (PFA) in PBS for 1 h in PCR tubes. Fresh PBT (PBS with 0.03% v/v Triton X-100) was used to rinse the wing discs three times immediately following fixation. PCR tubes containing wing discs in PBT were then placed on a nutator for 10 minutes at room temperature and then rinsed again with fresh PBT; this step was repeated for three nutation/rinsing intervals. PBT from after the third rinse was removed and 250 µL of 5% normal goat serum (NGS) in PBS was added to the PCR tube. Tubes were then agitated on a nutator for 45 minutes at room temperature. NGS was replaced with 250 µL of a primary antibody mixture prepared in a 5% NGS solution. Next, tubes were agitated on a nutator at 4 °C overnight. The following primary antibodies were used: Phospho-Smad1/5 (Ser463/465) (41D10) (1:300, Rabbit, Cell Signaling Technology #9516S), P-Histone H3 (1:500, Rabbit, Cell Signaling Technology #9701S), Phospho-Myosin Light Chain 2 (Ser19) (1:50, Rabbit, Cell Signaling Technology #3671S), α-Rho1 (1:10, Mouse, Developmental Studies Hybridoma Bank p1D9), Integrin betaPS (myospheroid) (1:5, Mouse, Developmental Studies Hybridoma Bank CF.6G11), α-Collagen IV antibody (1:5, Rabbit, Abcam ab6586). Three PBT rinses were performed the next day as was done after fixation. Tubes were then placed on a nutator for 20 minutes with fresh PBT and this was repeated for three nutation/rinsing intervals. After removing the PBT from the final rinse, 250 µL of a secondary antibody mixture corresponding to the primary antibodies along with DAPI, prepared in a 5% NGS solution, was added. Tubes were then agitated on a nutator for 2 hours at room temperature. The following dyes and secondary antibodies were α-Mouse Alexa Fluor™ 568 (1:500, Goat, Thermo Fisher Scientific A-11031), α-Rabbit Alexa Fluor™ 647(1:500, Goat, Thermo Fisher Scientific A32733), DAPI (1:500, Sigma Aldrich D9542), Fluorescein Phalloidin(1:500, Thermo Fisher Scientific F432).Three subsequent quick PBT rinses followed by three 20 minutes of agitation on the nutator with fresh PBT at room temperature were performed. Wing discs in PBT were left in the tube at 4℃ for an overnight wash. Four sets of double-layered scotch tape strips were used as spacers to create a square well. The spacers were positioned on the surface of a glass slide which prevents the coverslip from pressing on the wing discs. The wing discs were mounted within the well using Vectashield mounting medium and a cover slip was placed atop, aligned with the spacers.

S6. Supplementary Videos

Supplementary Movie 1: (Reference to Fig. 3E-i) Simulation showing tissue shape changes on increasing the ratio of apical to basal contractility in the medial domain of the pouch. The patterning of parameters ($k_{api,cont}/k_{bas,cont}$) has been defined according to the top panel in Fig. 3E-i.

Supplementary Movie 2: (Reference to Fig. 3E-ii) Simulation showing tissue shape changes on increasing the extracellular matrix (ECM) stiffness in the medial domain of the pouch. The patterning of the parameter ($k_{ecm,c}$) has been defined according to the top panel in Fig. 3E-ii.

Supplementary Movie 3: (Reference to Fig. 3E-iii) Simulation showing tissue shape changes on increasing the columnar cell-ECM adhesion in the lateral domains of the pouch as compared to the medial domain. The patterning of the parameter (k_{adhB}) has been defined according to the top panel in Fig. 3E-iii.

Supplementary Movie 4: (Reference to Fig. 4G') Simulation showing tissue shape changes upon varying the cell pressure by changing the control volume of cells such that the control volume decreases as one moves away from the center of the pouch. The patterning of the control volume (Ω_0) has been defined as the blue line in Fig 4E.

Supplementary Movie 5: (Reference to Fig. 6A-i) Simulation showing transition in tissue shape upon decreasing proliferation in only the posterior compartment (right hand side) of the wing imaginal disc. Proliferation was decreased by increasing the cell cycle length of epithelial cells in the posterior compartment by 400%.

Supplementary Movie 6: (Reference to Fig. 6A-ii) Simulation showing the "wild type" control simulation where cell proliferation is spatially homogeneous across the tissue. In other words, both the anterior and posterior compartments have the same cell division rate.

Supplementary Movie 7: (Reference to Fig. 6A-iii) Simulation showing transition in tissue shape upon increasing proliferation in only the posterior compartment (right hand side) of the wing imaginal disc. Proliferation was increased by decreasing the cell cycle length of epithelial cells in the posterior compartment by 50%.

Supplementary Movie 8: (Reference to Fig. 8D-ii) Simulation showing transition in tissue shape upon increasing proliferation and actomyosin contractility in only the posterior compartment (right hand side) of the wing imaginal disc. Proliferation of columnar cells was increased by 50% in the posterior compartment. Patterning of the actomyosin contractility parameter ($k_{api,cont}$) has been defined according to the top panel in Fig. 8D-ii.

Supplementary Movie 9: (Reference to Fig. 8D-iii) Simulation showing transition in tissue shape upon increasing proliferation and ECM stiffness in only the posterior compartment (right hand side) of the wing imaginal disc. Proliferation of columnar cells was increased by 50% in the posterior compartment. Patterning of the ECM stiffness parameter ($k_{ecm,c}$) has been defined according to the top panel in Fig. 8D-iii.

Supplementary Movie 10: (Reference to Fig. 8D-iv) Simulation showing transition in tissue shape upon increasing proliferation and cell-ECM adhesion in only the posterior compartment (right-hand side) of the wing imaginal disc. Proliferation of columnar cells was increased by 50% in the posterior compartment. Patterning of the cell-ECM adhesion parameter (k_{adhB}) has been defined according to the top panel in Fig. 8D-iv.

S7. Tables

Supplementary Table 1. Updated model parameters used in the Subcellular Element model.

Energy & model parameters	Definition	Interaction Type	Values	Source
E_v	Volume exclusion, membrane-membrane	Morse	$U_v = -W_v = 14.08\ nN\mu m$	Calibrated for numerical stability
			$\xi_v = 0.375\ \mu m$	Calibrated for numerical stability
			$\gamma_v = 0.094\ \mu m$	Calibrated for numerical stability
	Volume exclusion, membrane-nuclei	Morse	$U_v = -W_v = 14.08\ nN\mu m$	Calibrated for numerical stability
			$\xi_v = 0.32\ \mu m$	Calibrated for numerical stability
			$\gamma_v = 0.039\ \mu m$	Calibrated for numerical stability
	Volume exclusion, membrane-ECM	Morse	$U_v = -W_v = 14.08\ nN\mu m$	Calibrated for numerical stability
			$\xi_v = 0.1375\ \mu m$	Calibrated for numerical stability
			$\gamma_v = 0.033\ \mu m$	Calibrated for numerical stability
E_{nuc}	Size of nucleus	Morse	$U_{nuc} = W_{nuc} = 35.5\ nN\mu m$	Calibrated in this study
			$\xi_{nuc} = 0.392\ \mu m$	Calibrated in this study
			$\gamma_{nuc} = 5.88\ \mu m$	Calibrated in this study
	Interaction range	N/A	$2.1\ \mu m$	Calibrated in this study
E_{memb}	Cell membrane and actomyosin at the cortex of the cell	Spring	$k_{memb} = 1800\ nN/\mu m$	[46,47]
			$L0_{memb} = 0.0625\ \mu m$	[47]
$E_{memb,bend}$	Cell membrane and actomyosin at the cortex of the cell	Bending Spring	$k_{memb,bend} = 9\ nN\ \mu m$	[46,47] This work-based on simulation output as compared to tissue morphology
			$\theta_0 = \pi\ rad$	
$E_{api,cont}$	Apical actomyosin contractility	Spring	$k_{api,cont} = 9\ nN/\ \mu m$	Calibrated in this study
			$L0_{api,cont} = 0.03125\ \mu m$	Calibrated in this study
$E_{bas,cont}$	Basal actomyosin contractility	Spring	$k_{bas,cont} = 9\ nN/\mu m$	[47]
			$L0_{bas,cont} = 0.03125\ \mu m$	[47]
E_{vol}	Cytoplasm volume conservation	Lagrange Multiplier	$k_{vol} = 30\ nN\ /\ \mu m^2$	[47,75]
			$\Omega_{0pouch} = \Omega_{0perip} = 65\ \mu m^2$	[47]
			$\Omega_{0BC} = 20\ \mu m^2$	[47]
E_{adhL}	E-cadherin mediated cell-cell adhesion	Spring	$k_{adhL} = 200\ nN\ /\ \mu m$	[46]
			$L0_{adhL} = 0.0625\ \mu m$	[47]
E_{adhB}	Integrin mediated	Spring	$k_{adhB} = 400\ nN\ /\ \mu m$	[46,67]

	cell-ECM adhesion		$L0_{adhB} = 0.0625\ \mu m$	[47]
E_{adhA}	Columnar-squamous cell adhesion	Spring	$k_{adhA} = 200\ nN / \mu m$	[47]
			$L0_{adhA} = 0.0625\ \mu m$	[47]
E_{ecm}	ECM stiffness	Spring	$k_{ECM} = 4500\ nN / \mu m$	[76]
			$L0_{ecm,c} = 0.06\ \mu m$	[47]
			$L0_{ecm,s} = 0.06\ \mu m$	[47]
			$L0_{ecm,bc} = 0.06\ \mu m$	[47]
$E_{ecm,bend}$	ECM bending stiffness	Bending Spring	$k_{ecm,bend} = 6\ nN\ \mu m$	[46,47] This work- based on simulation output as compared to tissue morphology
			$\theta_0 = \pi\ rad$	
	ECM_C pre-strain	N/A	$c_1 = 540\ nN$	This work- based on simulation output as compared to tissue morphology
	ECM_{bc} pre-strain	N/A	$c_2 = 540\ nN$	This work- based on simulation output as compared to tissue morphology
h	Increment of the height of the portion of a cell with high actomyosin presence during mitotic rounding	N/A	$4.6703 \times 10^{-5} \mu m$ per simulation time step A.U.	Calibrated in this study
Probability of in-plane cell division	The likelihood of a newly created daughter cell resides in the same cross plane of the model wing disc	N/A	0.25	From current experimental measurements
Mitotic event marker	Progression (%) of cell cycle needed to enter mitosis	N/A	95	[77]

Supplementary Table 2. Altered model parameters for the *in-silico* model scenarios.

Case	Energy	Definition	Parameter multiplier for lateral regions	Parameter multiplier for medial regions	Parameter Value
**Case 1A**	$E_{api,cont}$	Apical actomyosin contractility	0.0625	1.333	$k_{api,cont} = 9\ nN/ \mu m$
	$E_{bas,cont}$	Basal actomyosin contractility	1.5	2.0	$k_{bas,cont} = 9\ nN/ \mu m$

**Case 1B**	$E_{api,cont}$	Apical actomyosin contractility	0.0625	0.0029	$k_{api,cont} = 9\, nN/\, \mu m$
	$E_{bas,cont}$	Basal actomyosin contractility	1.5	1.125	$k_{bas,cont} = 9\, nN/\, \mu m$
**Case 2A**	$E_{ecm,c}$	ECM stiffness	0.5	1.0	$k_{ecm,c} = 4500\, nN\, /\, \mu m$
**Case 2B**	$E_{ecm,c}$	ECM stiffness	1.0	0.5	$k_{ecm,c} = 4500\, nN\, /\, \mu m$
**Case 3A**	E_{memb}	Membrane stiffness	0.5	1.0	$k_{memb,lateral} = 1800\, nN/\mu m$
**Case 3B**	E_{memb}	Membrane stiffness	1.0	0.5	$k_{memb,lateral} = 1800\, nN/\mu m$
**Case 3B'**	E_{memb}	Membrane stiffness	1.0	0.1	$k_{memb,lateral} = 1800\, nN/\mu m$
**Case 4A**	E_{memb}	Membrane stiffness	0.5	1.0	$k_{memb,basal} = 1800\, nN/\mu m$
**Case 4B**	E_{memb}	Membrane stiffness	1.0	0.5	$k_{memb,basal} = 1800\, nN/\mu m$
**Case 5A**	E_{memb}	Membrane stiffness	1.0	0.5	$L0_{memb,lateral} = 0.0625\, \mu m$
**Case 5B**	E_{memb}	Membrane stiffness	0.5	1.0	$L0_{memb,lateral} = 0.0625\, \mu m$
**Case 6A**	E_{memb}	Membrane stiffness	1.0	0.5	$L0_{memb,basal} = 0.0625\, \mu m$
**Case 6B**	E_{memb}	Membrane stiffness	0.5	1.0	$L0_{memb,basal} = 0.0625\, \mu m$
**Case 7A**	E_{adhB}	Integrin-mediated cell-ECM adhesion	1.5	1.0	$k_{adhB} = 400\, nN\, /\, \mu m$
**Case 7B**	E_{adhB}	Integrin-mediated cell-ECM adhesion	2.0	1.0	$k_{adhB} = 400\, nN\, /\, \mu m$

S8. Statistical tests

S-8.1 Statistical tests employed in the main manuscript. For all the experiments except for the staged Oregon-R discs, a total number of samples greater than 5 was used, except if indicated. Immunohistochemistry and dissection of discs from younger larvae is challenging and we have indicated the sample sizes as insets. A student t-test[5,6] with unequal variances was used to measure the statistical significance between data from any two experimental groups (*$p \in [0.05, 0.01)$, **$p \in [0.01, 0.001)$, ***$p \leq 0{,}001$). Statistics was carried out using the stats module of Scipy[71]. All the data visualization was carried out using either matplotlib and seaborn[78] libraries of Python or MATLAB[1].

Detailed statistics tables corresponding to specific figures within this manuscript can be found below.

Supplementary Table 3. Statistics table Corresponding to Figure 2E.

Id	Groups Compared	Mean Difference	t-Stat	p-value	95% CI Lower	95% CI Upper
1	72-78 h	5.44	-0.25988	0.81177	-34.05	23.16
2	72-84 h	60.92	-2.77229	0.050213	-107.55	-14.28
3	72-90 h	98.69	-2.60318	0.080156	-191.09	-6.28
4	72-96 h	183.91	-4.96894	0.000771	-252.35	-115.47
5	78-84 h	55.47	-2.16009	0.119565	-110.9	-0.04
6	78-90 h	93.24	-1.93354	0.192855	-236.73	50.24
7	78-96 h	178.46	-3.93141	0.004348	-246.06	-110.87
8	84-90 h	37.77	-0.92768	0.422002	-100.13	24.59
9	84-96 h	122.99	-3.27509	0.009604	-176.13	-69.85
10	90-96 h	85.22	-1.76123	0.116228	-134.79	-35.65

Supplementary Table 4. Statistics Table corresponding to Figure 2E'.

Id	Groups Compared	Mean Difference	t-Stat	p-value	95% CI Lower	95% CI Upper
1	72-78 h	38.04	1.959782	0.107318	8.07	68.02
2	72-84 h	51.28	2.585635	0.049096	16.07	86.5
3	72-90 h	46.79	1.963073	0.121127	7.69	85.89
4	72-96 h	53.02	3.65614	0.008112	25.83	80.21
5	78-84 h	13.24	1.263489	0.275037	-2.15	28.63
6	78-90 h	8.75	0.932211	0.419993	-5.64	23.14
7	78-96 h	14.98	2.12612	0.077624	4.75	25.21
8	84-90 h	4.49	-0.39791	0.717322	-20.14	11.15
9	84-96 h	1.74	0.219975	0.833184	-6.95	10.42
10	90-96 h	6.23	0.879438	0.419416	-1.84	14.3

Supplementary Table 5. Statistics Table Corresponding to Figure 3D-ii.

Id	Groups Compared	Mean Difference	t-Stat	p-value	95% CI Lower	95% CI Upper
1	72-96 h	2.83	4.938096	0.000589	1.77	3.89

Supplementary Table 6. Statistics Table Corresponding to Figure 4A.

Id	Groups Compared	Mean Difference	t-Stat	p-value	95% CI Lower	95% CI Upper
1	72-78 h	1.86	-0.88025	0.428435	-4.72	1.01
2	72-84 h	4.55	-2.13869	0.099234	-8.43	-0.68
3	72-90 h	2.57	-0.84165	0.46178	-7.15	2.01
4	72-96 h	10.16	-4.33319	0.007477	-16.1	-4.22
5	78-84 h	2.7	-4.70331	0.009286	-4.52	-0.88
6	78-90 h	0.71	-0.46818	0.671574	-2.83	1.41

7	78-96 h	8.31	-5.19411	0.003484	-13	-3.61
8	84-90 h	1.99	1.277334	0.291371	-0.62	4.6
9	84-96 h	5.61	-3.47086	0.017833	-9.07	-2.14
10	90-96 h	7.6	-3.27702	0.030586	-12.81	-2.39

Supplementary Table 7. Statistics Table Corresponding to Figure 4A'.

Id	Groups Compared	Mean Difference	t-Stat	p-value	95% CI Lower	95% CI Upper
1	72-78 h	0.06	-0.05885	0.955897	-1.43	1.3
2	72-84 h	1.4	-1.04851	0.353575	-3.27	0.47
3	72-90 h	2.02	-137%	0.265189	-4.57	0.52
4	72-96 h	6.69	-5.21875	0.003413	-10.47	-2.91
5	78-84 h	1.33	-0.99232	0.37721	-3.2	0.53
6	78-90 h	1.96	-1.30901	0.281772	-4.49	0.57
7	78-96 h	6.63	-5.13796	0.003652	-10.38	-2.87
8	84-90 h	0.62	-0.34883	0.750263	-3.08	1.84
9	84-96 h	5.29	-3.68338	0.014243	-8.51	-2.08
10	90-96 h	4.67	-2.79372	0.049125	-8.03	-1.31

Supplementary Table 8. Statistics Table corresponding to Figure 4D

Id	Groups Compared	Mean Difference	t-Stat	p-value	95% CI Lower	95% CI Upper
1	*RyR*^{RNAi}-*mys*^{RNAi}	10.79	2.05386	0.070174	3.99	17.59
2	*RyR*^{RNAi}-*Rho1*^{RNAi}	22.22	-2.94149	0.011458	-32.17	-12.28

Supplementary Table 9. Statistics Table corresponding to Figure 4D'

Id	Groups Compared	Mean Difference	t-Stat	p-value	95% CI Lower	95% CI Upper
1	*RyR*^{RNAi}-*mys*^{RNAi}	26.78	2.810806	0.02035	12.83	40.73
2	*RyR*^{RNAi}-*Rho1*^{RNAi}	21.96	-1.72012	0.10911	-36.39	-7.54

Supplementary Table 10. Statistics Table corresponding to Figure 5B

Id	Groups Compared	Mean Difference	t-Stat	p-value	95% CI Lower	95% CI Upper
1	72-96 h	0.2	-2.687022	0.019778	-0.3	-0.11

Supplementary Table 11. Statistics Table corresponding to Figure 7C-ii.

Id	Groups Compared	Mean Difference	t-Stat	p-value	95% CI Lower	95% CI Upper
1	*en-InsR*^{DN}	0.2	3.503584	0.008037	0.1	0.29

Supplementary Table 12. Statistics Table corresponding to Figure 7Fi-iii.

Fig. No.	Groups Compared	Mean Difference	t-Stat	p-value	95% CI Lower	95% CI Upper

I (βPS)	*en-InsR^{DN}*	0.57	-0.03706	0.97134	-17.05	15.91
ii (Rho1)	*en-InsR^{DN}*	21.68	3.891696	0.004598	11.59	31.78
iii (pMyoII)	*en-InsR^{DN}*	35.85	3.179858	0.008763	19.44	52.25

Supplementary Table 13. Statistics Table corresponding to Figure 7H-ii.

Id	Groups Compared	Mean Difference	t-Stat	p-value	95% CI Lower	95% CI Upper
1	*en-InsR^{CA}*	5.66	-3.70618	0.007593	-8.55	-2.77

Supplementary Table 14. Statistics Table corresponding to Figure 7N.

Id	Groups Compared	Mean Difference	t-Stat	p-value	95% CI Lower	95% CI Upper
1	*en-Tkv^{CA}*	23.04	-1.26442	0.230087	-43.13	-2.94
2	*en-Myc*	58.84	-2.80627	0.014006	-85.14	-32.55

Supplementary Table 15. Statistics Table corresponding to Figure 7O-i

Id	Groups Compared	Mean Difference	t-Stat	p-value	95% CI Lower	95% CI Upper
1	*en-Tkv^{CA}*	77.51	-4.64922	0.001203	-110.37	-44.65
2	*en-Myc*	46.56	5.1125	0.000199	30.45	6268%

Supplementary Table 16. Statistics Table corresponding to Figure 7O-ii

Id	Groups Compared	Mean Difference	t-Stat	p-value	95% CI Lower	95% CI Upper
1	*en-Tkv^{CA}*	54.41	-1.04252	0.324365	-113.46	4.63
2	*en-Myc*	25.91	2.373606	0.033708	12.58	39.24

Supplementary Table 17. Statistics Table corresponding to Figure 8A

Id	Groups Compared	Mean Difference	t-Stat	p-value	95% CI Lower	95% CI Upper
1	*en-Tkv^{CA}*	0.36	2.234634	0.055891	0.14	0.58
2	*en-Myc*	0.43	4.303504	0.001249	0.26	0.6

S-8.2 Statistical test for SI Figure 19. An F-test was calculated using the vartest2 function in MATLAB[1] to evaluate the statistical significance of increasing proliferation. The h and p-values have been listed below. When using this function, if $h = 1$ it means that the null hypothesis has been rejected at the 5% significance level and otherwise when $h = 0$.

Supplementary Table 18. F-test for SI Figure 19.

	control vs. case 1 (1.4 x control)	$h = 1$
		$p = 2.9728e^{-22}$

**Medial Region**	control vs. case 2 (2 x control)	$h = 1$
		$p = 1.0107e^{-07}$
	control vs. case 3 (10 x control)	$h = 1$
		$p = 1.1543e^{-36}$
	control vs. case 4 (20 x control)	$h = 1$
		$p = 1.3591e^{-29}$
**Lateral Regions**	control vs. case 1 (1.4 x control)	$h = 0$
		$p = 0.5555$
	control vs. case 2 (2 x control)	$h = 0$
		$p = 0.9767$
	control vs. case 3 (10 x control)	$h = 0$
		$p = 0.9205$
	control vs. case 4 (20 x control)	$h = 0$
		$p = 0.4146$

S9. SI References

1. The MathWorks Inc. MATLAB 9.9 (2020b). *Natick Mass.* (2020).
2. Duffy, J. B. GAL4 system in drosophila: A fly geneticist's swiss army knife. *genesis* **34**, 1–15 (2002).
3. Johnston, L. A., Prober, D. A., Edgar, B. A., Eisenman, R. N. & Gallant, P. Drosophila myc Regulates Cellular Growth during Development. *Cell* **98**, 779–790 (1999).

4. Kramer, O. Scikit-Learn. in *Machine Learning for Evolution Strategies* (ed. Kramer, O.) 45–53 (Springer International Publishing, Cham, 2016). doi:10.1007/978-3-319-33383-0_5.
5. Montgomery, D. C., Runger, G. C. & Hubele, N. F. *Engineering Statistics*. (Wiley, 2007).
6. Bethea, R. M., Duran, B. S. & Boullion, T. L. *Statistical Methods for Engineers and Scientists*. (M. Dekker, 1975).
7. Schindelin, J. *et al.* Fiji: an open-source platform for biological-image analysis. *Nat. Methods* **9**, 676–682 (2012).
8. Mao, Y. *et al.* Differential proliferation rates generate patterns of mechanical tension that orient tissue growth. *EMBO J.* **32**, 2790–2803 (2013).
9. Straus, D. S. Effects of insulin on cellular growth and proliferation. *Life Sci.* **29**, 2131–2139 (1981).
10. Kimura, K. *et al.* Regulation of myosin phosphatase by Rho and Rho-associated kinase (Rho-kinase). *Science* **273**, 245–248 (1996).
11. Eisenhoffer, G. T. *et al.* Crowding induces live cell extrusion to maintain homeostatic cell numbers in epithelia. *Nature* **484**, 546–549 (2012).
12. Marinari, E. *et al.* Live-cell delamination counterbalances epithelial growth to limit tissue overcrowding. *Nature* **484**, 542–545 (2012).
13. Ohsawa, S., Vaughen, J. & Igaki, T. Cell Extrusion: A Stress-Responsive Force for Good or Evil in Epithelial Homeostasis. *Dev. Cell* **44**, 284–296 (2018).
14. Fernandez-Gonzalez, R. & Zallen, J. A. Feeling the Squeeze: Live-Cell Extrusion Limits Cell Density in Epithelia. *Cell* **149**, 965–967 (2012).
15. Gudipaty, S. A. *et al.* Mechanical stretch triggers rapid epithelial cell division through Piezo1. *Nature* **543**, 118–121 (2017).
16. Munjal, A., Philippe, J.-M., Munro, E. & Lecuit, T. A self-organized biomechanical network drives shape changes during tissue morphogenesis. *Nature* **524**, 351–355 (2015).

17. Velagala, V., Chen, W., Alber, M. & Zartman, J. J. Chapter 4.1 - Multiscale Models Coupling Chemical Signaling and Mechanical Properties for Studying Tissue Growth. in *Mechanobiology* (ed. Niebur, G. L.) 173–195 (Elsevier, 2020). doi:10.1016/B978-0-12-817931-4.00010-8.

18. Keller, A., Lanfranconi, F. & Aegerter, C. M. The influence of geometry on the elastic properties of the Drosophila wing disc. *Phys. Stat. Mech. Its Appl.* **510**, 208–218 (2018).

19. Levis, M. *et al.* Microfluidics on the fly: Inexpensive rapid fabrication of thermally laminated microfluidic devices for live imaging and multimodal perturbations of multicellular systems. *Biomicrofluidics* **13**, 024111 (2019).

20. Pryor, R. W. *Multiphysics Modeling Using COMSOL®: A First Principles Approach*. (Jones & Bartlett Publishers, 2009).

21. Harmansa, S., Erlich, A., Eloy, C., Zurlo, G. & Lecuit, T. Growth anisotropy of the extracellular matrix shapes a developing organ. *Nat. Commun.* **14**, 1220 (2023).

22. Osborne, J. M., Fletcher, A. G., Pitt-Francis, J. M., Maini, P. K. & Gavaghan, D. J. Comparing individual-based approaches to modelling the self-organization of multicellular tissues. *PLOS Comput. Biol.* **13**, e1005387 (2017).

23. Honda, H. & Nagai, T. Cell models lead to understanding of multi-cellular morphogenesis consisting of successive self-construction of cells. *J. Biochem. (Tokyo)* **157**, 129–136 (2015).

24. Chen, N., Glazier, J. A., Izaguirre, J. A. & Alber, M. S. A parallel implementation of the Cellular Potts Model for simulation of cell-based morphogenesis. *Comput. Phys. Commun.* **176**, 670–681 (2007).

25. Popławski, N. J., Swat, M., Gens, J. S. & Glazier, J. A. Adhesion between cells, diffusion of growth factors, and elasticity of the AER produce the paddle shape of the chick limb. *Phys. A* **373**, 521–532 (2007).

26. Chaturvedi, R. *et al.* On multiscale approaches to three-dimensional modelling of morphogenesis. *J. R. Soc. Interface* **2**, 237–253 (2005).

27. Glazier, J. A. & Graner, F. Simulation of the differential adhesion driven rearrangement of biological cells. *Phys. Rev. E Stat. Phys. Plasmas Fluids Relat. Interdiscip. Top.* **47**, 2128–2154 (1993).

28. Graner, F. & Glazier, J. A. Simulation of biological cell sorting using a two-dimensional extended Potts model. *Phys. Rev. Lett.* **69**, 2013–2016 (1992).

29. Izaguirre, J. A. *et al.* CompuCell, a multi-model framework for simulation of morphogenesis. *Bioinforma. Oxf. Engl.* **20**, 1129–1137 (2004).

30. Fletcher, A. G., Osterfield, M., Baker, R. E. & Shvartsman, S. Y. Vertex Models of Epithelial Morphogenesis. *Biophys. J.* **106**, 2291–2304 (2014).

31. Nagai, T. & Honda, H. Computer simulation of wound closure in epithelial tissues: cell-basal-lamina adhesion. *Phys. Rev. E Stat. Nonlin. Soft Matter Phys.* **80**, 061903 (2009).

32. Nagai, T., Honda, H. & Takemura, M. Simulation of Cell Patterning Triggered by Cell Death and Differential Adhesion in Drosophila Wing. *Biophys. J.* **114**, 958–967 (2018).

33. Okuda, S., Inoue, Y. & Adachi, T. Three-dimensional vertex model for simulating multicellular morphogenesis. *Biophys. Physicobiology* **12**, 13–20 (2015).

34. Osterfield, M., Du, X., Schüpbach, T., Wieschaus, E. & Shvartsman, S. Y. Three-dimensional epithelial morphogenesis in the developing Drosophila egg. *Dev. Cell* **24**, 400–410 (2013).

35. Farhadifar, R., Röper, J.-C., Aigouy, B., Eaton, S. & Jülicher, F. The influence of cell mechanics, cell-cell interactions, and proliferation on epithelial packing. *Curr. Biol. CB* **17**, 2095–2104 (2007).

36. Aegerter-Wilmsen, T. *et al.* Integrating force-sensing and signaling pathways in a model for the regulation of wing imaginal disc size. *Dev. Camb. Engl.* **139**, 3221–3231 (2012).

37. Yu, J. C. & Fernandez-Gonzalez, R. Quantitative modelling of epithelial morphogenesis: integrating cell mechanics and molecular dynamics. *Semin. Cell Dev. Biol.* **67**, 153–160 (2017).

38. Sussman, D. M. cellGPU: massively parallel simulations of dynamic vertex models. *Comput. Phys. Commun.* **219**, 400–406 (2017).

39. Honda, H. & Nagai, T. *Mathematical Models of Cell-Based Morphogenesis: Passive and Active Remodeling*. (Springer Nature, Singapore, 2022). doi:10.1007/978-981-19-2916-8.

40. Nagai, T. & Honda, H. Computer simulation of wound closure in epithelial tissues: cell-basal-lamina adhesion. *Phys. Rev. E Stat. Nonlin. Soft Matter Phys.* **80**, 061903 (2009).

41. Nagai, T., Honda, H. & Takemura, M. Simulation of Cell Patterning Triggered by Cell Death and Differential Adhesion in Drosophila Wing. *Biophys. J.* **114**, 958–967 (2018).

42. Okuda, S., Inoue, Y. & Adachi, T. Three-dimensional vertex model for simulating multicellular morphogenesis. *Biophys. Physicobiology* **12**, 13–20 (2015).

43. Smallwood, R. Computational modeling of epithelial tissues. *WIREs Syst. Biol. Med.* **1**, 191–201 (2009).

44. Rauzi, M., Hočevar Brezavšček, A., Ziherl, P. & Leptin, M. Physical Models of Mesoderm Invagination in Drosophila Embryo. *Biophys. J.* **105**, 3–10 (2013).

45. Tozluoğlu, M. *et al.* Planar Differential Growth Rates Initiate Precise Fold Positions in Complex Epithelia. *Dev. Cell* **51**, 299-312.e4 (2019).

46. Nematbakhsh, A. *et al.* Multi-scale computational study of the mechanical regulation of cell mitotic rounding in epithelia. *PLOS Comput. Biol.* **13**, e1005533 (2017).

47. Nematbakhsh, A. *et al.* Epithelial organ shape is generated by patterned actomyosin contractility and maintained by the extracellular matrix. *bioRxiv* 2020.01.22.915272 (2020) doi:10.1101/2020.01.22.915272.

48. Micoulet, A., Spatz, J. P. & Ott, A. Mechanical response analysis and power generation by single-cell stretching. *Chemphyschem Eur. J. Chem. Phys. Phys. Chem.* **6**, 663–670 (2005).

49. Kuznetsova, T. G., Starodubtseva, M. N., Yegorenkov, N. I., Chizhik, S. A. & Zhdanov, R. I. Atomic force microscopy probing of cell elasticity. *Micron Oxf. Engl. 1993* **38**, 824–833 (2007).

50. Laurent, V. M. *et al.* Gradient of Rigidity in the Lamellipodia of Migrating Cells Revealed by Atomic Force Microscopy. *Biophys. J.* **89**, 667–675 (2005).

51. Sim, J. Y. *et al.* Spatial distribution of cell-cell and cell-ECM adhesions regulates force balance while main-taining E-cadherin molecular tension in cell pairs. *Mol. Biol. Cell* **26**, 2456–2465 (2015).

52. Chu, Y.-S. *et al.* Force measurements in E-cadherin–mediated cell doublets reveal rapid adhesion strengthened by actin cytoskeleton remodeling through Rac and Cdc42. *J. Cell Biol.* **167**, 1183–1194 (2004).

53. Marino, S., Hogue, I. B., Ray, C. J. & Kirschner, D. E. A methodology for performing global uncertainty and sensitivity analysis in systems biology. *J. Theor. Biol.* **254**, 178–196 (2008).

54. Fazeli, E. *et al.* Automated cell tracking using StarDist and TrackMate. *F1000Research* **9**, 1279 (2020).

55. Marin-Riera, M., Brun-Usan, M., Zimm, R., Välikangas, T. & Salazar-Ciudad, I. Computational modeling of development by epithelia, mesenchyme and their interactions: a unified model. *Bioinformatics* **32**, 219–225 (2016).

56. Pivkin, I. V. & Karniadakis, G. E. Accurate coarse-grained modeling of red blood cells. *Phys. Rev. Lett.* **101**, 118105 (2008).

57. Kirkland, N. J. *et al.* Tissue Mechanics Regulate Mitotic Nuclear Dynamics during Epithelial Development. *Curr. Biol.* **30**, 2419-2432.e4 (2020).

58. Tozluoğlu, M. *et al.* Planar Differential Growth Rates Initiate Precise Fold Positions in Complex Epithelia. *Dev. Cell* **51**, 299-312.e4 (2019).

59. Ioannou, F., Dawi, M. A., Tetley, R. J., Mao, Y. & Muñoz, J. J. Development of a New 3D Hybrid Model for Epithelia Morphogenesis. *Front. Bioeng. Biotechnol.* **8**, (2020).

60. Hughes, A. J. *et al.* Engineered Tissue Folding by Mechanical Compaction of the Mesenchyme. *Dev. Cell* **44**, 165-178.e6 (2018).

61. Zmurchok, C., Bhaskar, D. & Edelstein-Keshet, L. Coupling mechanical tension and GTPase signaling to generate cell and tissue dynamics. *Phys. Biol.* **15**, 046004 (2018).

62. Newman, T. J. Modeling multicellular systems using subcellular elements. *Math. Biosci. Eng. MBE* **2**, 613–624 (2005).

63. Sandersius, S. A., Weijer, C. J. & Newman, T. J. Emergent cell and tissue dynamics from subcellular modeling of active biomechanical processes. *Phys. Biol.* **8**, 045007 (2011).

64. Adam, I., Bagnoli, F., Fanelli, D., Mahadevan, L. & Paoletti, P. Prestrain-induced contraction in one-dimensional random elastic chains. *Phys. Rev. E* **105**, 065002 (2022).

65. Freeman, J. W. & Silver, F. H. The Effects of Prestrain and Collagen Fibril Alignment on In Vitro Mineralization of Self-Assembled Collagen Fibers. *Connect. Tissue Res.* **46**, 107–115 (2005).

66. Micoulet, A., Spatz, J. P. & Ott, A. Mechanical response analysis and power generation by single-cell stretching. *Chemphyschem Eur. J. Chem. Phys. Phys. Chem.* **6**, 663–670 (2005).

67. Sim, J. Y. *et al.* Spatial distribution of cell-cell and cell-ECM adhesions regulates force balance while main-taining E-cadherin molecular tension in cell pairs. *Mol. Biol. Cell* **26**, 2456–2465 (2015).

68. Chu, Y.-S. *et al.* Force measurements in E-cadherin–mediated cell doublets reveal rapid adhesion strengthened by actin cytoskeleton remodeling through Rac and Cdc42. *J. Cell Biol.* **167**, 1183–1194 (2004).

69. Marino, S., Hogue, I. B., Ray, C. J. & Kirschner, D. E. A methodology for performing global uncertainty and sensitivity analysis in systems biology. *J. Theor. Biol.* **254**, 178–196 (2008).

70. Bradski, G. & Kaehler, A. OpenCV. *Dr. Dobb's journal of software tools* (2000).

71. Virtanen, P. *et al.* SciPy 1.0: fundamental algorithms for scientific computing in Python. *Nat. Methods* **17**, 261–272 (2020).

72. Prautzsch, H., Boehm, W. & Paluszny, M. *Bézier and B-Spline Techniques*. (Springer Berlin Heidelberg, Berlin, Heidelberg, 2002). doi:10.1007/978-3-662-04919-8.

73. Mary, H. & Brouhard, G. J. Kappa (κ): Analysis of Curvature in Biological Image Data using B-splines. 14.

74. Fazeli, E. *et al.* Automated cell tracking using StarDist and TrackMate. *F1000Research* **9**, 1279 (2020).

75. Pivkin, I. V. & Karniadakis, G. E. Accurate coarse-grained modeling of red blood cells. *Phys. Rev. Lett.* **101**, 118105 (2008).

76. Keller, A., Lanfranconi, F. & Aegerter, C. M. The influence of geometry on the elastic properties of the Drosophila wing disc. *Phys. Stat. Mech. Its Appl.* **510**, 208–218 (2018).

77. Kirkland, N. J. *et al.* Tissue Mechanics Regulate Mitotic Nuclear Dynamics during Epithelial Development. *Curr. Biol. CB* **30**, 2419-2432.e4 (2020).

78. Bisong, E. Matplotlib and Seaborn. in *Building Machine Learning and Deep Learning Models on Google Cloud Platform: A Comprehensive Guide for Beginners* (ed. Bisong, E.) 151–165 (Apress, Berkeley, CA, 2019). doi:10.1007/978-1-4842-4470-8_12.
