# Supplementary material for: Balancing competing effects of tissue growth and cytoskeletal regulation during Drosophila wing disc development
Source: Nat Commun. 2024 Mar 20;15:2477. doi: 10.1038/s41467-024-46698-7 (PMC10954670; doi:10.1038/s41467-024-46698-7)
Supplement: Supplementary file 2 — Description of Additional Supplementary Files [file 41467_2024_46698_MOESM2_ESM.pdf]

Description of Additional Supplementary Files:

Supplementary Movie 1: (Reference to Fig. 3E-i) Simulation showing tissue shape changes on increasing the ratio of apical to basal contractility in the medial domain of the pouch. The patterning of parameters ($k_{api,cont}/ k_{bas,cont}$) has been defined according to the top panel in Fig. 3E-i.

Supplementary Movie 2: (Reference to Fig. 3E-ii) Simulation showing tissue shape changes on increasing the extracellular matrix (ECM) stiffness in the medial domain of the pouch. The patterning of the parameter ($k_{ecm,c}$) has been defined according to the top panel in Fig. 3E-ii.

Supplementary Movie 3: (Reference to Fig. 3E-iii) Simulation showing tissue shape changes on increasing the columnar cell-ECM adhesion in the lateral domains of the pouch as compared to the medial domain. The patterning of the parameter (k_{adhB}) has been defined according to the top panel in Fig. 3E-iii.

Supplementary Movie 4: (Reference to Fig. 4G') Simulation showing tissue shape changes upon varying the cell pressure by changing the control volume of cells such that the control volume decreases as one moves away from the center of the pouch. The patterning of the control volume (Ω_0) has been defined as the blue line in Fig 4E.

Supplementary Movie 5: (Reference to Fig. 6A-i) Simulation showing transition in tissue shape upon decreasing proliferation in only the posterior compartment (right hand side) of the wing imaginal disc. Proliferation was decreased by increasing the cell cycle length of epithelial cells in the posterior compartment by 400%.

Supplementary Movie 6: (Reference to Fig. 6A-ii) Simulation showing the “wild type” control simulation where cell proliferation is spatially homogeneous across the tissue. In other words, both the anterior and posterior compartments have the same cell division rate.

Supplementary Movie 7: (Reference to Fig. 6A-iii) Simulation showing transition in tissue shape upon increasing proliferation in only the posterior compartment (right hand side) of the wing imaginal disc. Proliferation was increased by decreasing the cell cycle length of epithelial cells in the posterior compartment by 50%.

Supplementary Movie 8: (Reference to Fig. 8D-ii) Simulation showing transition in tissue shape upon increasing proliferation and actomyosin contractility in only the posterior compartment (right hand side) of the wing imaginal disc. Proliferation of columnar cells was increased by 50% in the

posterior compartment. Patterning of the actomyosin contractility parameter ($kapi,cont$) has been defined according to the top panel in Fig. 8D-ii.

Supplementary Movie 9: (Reference to Fig. 8D-iii) Simulation showing transition in tissue shape upon increasing proliferation and ECM stiffness in only the posterior compartment (right hand side) of the wing imaginal disc. Proliferation of columnar cells was increased by 50% in the posterior compartment. Patterning of the ECM stiffness parameter ($kecm,c$) has been defined according to the top panel in Fig. 8D-iii.

Supplementary Movie 10: (Reference to Fig. 8D-iv) Simulation showing transition in tissue shape upon increasing proliferation and cell-ECM adhesion in only the posterior compartment (right-hand side) of the wing imaginal disc. Proliferation of columnar cells was increased by 50% in the posterior compartment. Patterning of the cell-ECM adhesion parameter ($kadhB$) has been defined according to the top panel in Fig. 8D-iv.
