# Supplementary material for: Balancing competing effects of tissue growth and cytoskeletal regulation during Drosophila wing disc development
Source: Nat Commun. 2024 Mar 20;15:2477. doi: 10.1038/s41467-024-46698-7 (PMC10954670; doi:10.1038/s41467-024-46698-7)
Supplement: Supplementary file 13 — Reporting Summary [file 41467_2024_46698_MOESM13_ESM.pdf]

nature portfolio

Corresponding author(s): Jeremiah J. Zartman
Mark Alber

Last updated by author(s): Feb 29, 2024

Reporting Summary

Nature Portfolio wishes to improve the reproducibility of the work that we publish. This form provides structure for consistency and transparency in reporting. For further information on Nature Portfolio policies, see our Editorial Policies and the Editorial Policy Checklist.

Statistics

For all statistical analyses, confirm that the following items are present in the figure legend, table legend, main text, or Methods section.

n/a	Confirmed	
☐	☒	The exact sample size (*n*) for each experimental group/condition, given as a discrete number and unit of measurement
☐	☒	A statement on whether measurements were taken from distinct samples or whether the same sample was measured repeatedly
☐	☒	The statistical test(s) used AND whether they are one- or two-sided<br>*Only common tests should be described solely by name; describe more complex techniques in the Methods section.*
☒	☐	A description of all covariates tested
☒	☐	A description of any assumptions or corrections, such as tests of normality and adjustment for multiple comparisons
☐	☒	A full description of the statistical parameters including central tendency (e.g. means) or other basic estimates (e.g. regression coefficient) AND variation (e.g. standard deviation) or associated estimates of uncertainty (e.g. confidence intervals)
☐	☒	For null hypothesis testing, the test statistic (e.g. *F*, *t*, *r*) with confidence intervals, effect sizes, degrees of freedom and *P* value noted<br>*Give P values as exact values whenever suitable.*
☒	☐	For Bayesian analysis, information on the choice of priors and Markov chain Monte Carlo settings
☒	☐	For hierarchical and complex designs, identification of the appropriate level for tests and full reporting of outcomes
☐	☒	Estimates of effect sizes (e.g. Cohen's *d*, Pearson's *r*), indicating how they were calculated

Our web collection on statistics for biologists contains articles on many of the points above.

Software and code

Policy information about availability of computer code

Data collection	The wing imaginal discs were imaged using a Nikon Eclipse Ti confocal microscope with a Yokogawa spinning disc and MicroPoint laser ablation system, and a Nikon A1R-MP laser scanning confocal microscope. For the two confocal microscopes, image data were collected on an IXonEM+colled CCD camera (Andor Technology, South Windsor, CT) using MetaMorph v7.7.9 software (Molecular Devices, Sunnyvale, CA) and NIS-Elements software, respectively. Discs were imaged throughout the entire depth of z-planes with a step size of 0.8-1 µm, depending on sample thickness, with a 40x and 60x oil objective with 200 ms exposure time, and 50 nW, 405 nm, 488 nm, 561 nm, and 640 nm laser exposure at 44% laser intensity. The imaging was performed from apical to basal surface so that peripodial cells were imaged first followed by the columnar cells of the wing disc. Optical slices were taken at distances equalling half the compartment length. Tiling was performed on images to get the entire sample in the field of view, and QuickStich was utilized to stitch individual tiles.
Data analysis	As image preprocessing steps, we primarily used rolling ball algorithm for background subtraction. Deep learning based trained models were employed using CSBDeep and StarDist for denoising and segmenting nuclei respectively. All the preprocessing steps were carried out using ImageJ (v1.53t). In house pipelines were developed using Matlab (R2021b) and Python (3.6.5) for quantification of morphological and signaling features of the wing imaginal disc. All the data analysis and visualization codes have been uploaded as source data files and included in the data availability section. The entire data analysis and visualization was done in Python using the following packages: Matplotlib (v2.2.2), seaborn (v 0.8.1), numpy (v1.19.5), pandas (v0.23.0), scipy (v1.5.4), scikit-learn (v0.21.3), OpenCV (v3.4.1).

For manuscripts utilizing custom algorithms or software that are central to the research but not yet described in published literature, software must be made available to editors and reviewers. We strongly encourage code deposition in a community repository (e.g. GitHub). See the Nature Portfolio guidelines for submitting code & software for further information.

Data

Policy information about availability of data

All manuscripts must include a data availability statement. This statement should provide the following information, where applicable:

- Accession codes, unique identifiers, or web links for publicly available datasets
- A description of any restrictions on data availability
- For clinical datasets or third party data, please ensure that the statement adheres to our policy

The processed data and codes for experimental data analysis used in this study are provided in the Supplementary Information/Source Data file. The 3D confocal microscopy data is available on request from the corresponding author (jzartman@ned.edu). All the codes are available as open access through the source data provided within this manuscript.

Research involving human participants, their data, or biological material

Policy information about studies with human participants or human data. See also policy information about sex, gender (identity/presentation), and sexual orientation and race, ethnicity and racism.

Reporting on sex and gender	N/A
Reporting on race, ethnicity, or other socially relevant groupings	N/A
Population characteristics	N/A
Recruitment	N/A
Ethics oversight	N/A

Note that full information on the approval of the study protocol must also be provided in the manuscript.

Field-specific reporting

Please select the one below that is the best fit for your research. If you are not sure, read the appropriate sections before making your selection.

[x] Life sciences [ ] Behavioural & social sciences [ ] Ecological, evolutionary & environmental sciences

For a reference copy of the document with all sections, see nature.com/documents/nr-reporting-summary-flat.pdf

Life sciences study design

All studies must disclose on these points even when the disclosure is negative.

Sample size	For wing imaginal disc samples collected during the staging of wild-type Oregon-R, a sample size >=4 was kept for earlier stages of development due to technical challenges. The sample sizes are additionally included as insets within the plots (Fig. 1 & 2). For all other experiments involving fixed tissue imaging a sample size of N>5 was kept consistent.
Data exclusions	Any sample that was either torn during the immunohistochemistry assay or suffered excessive secondary antibody accumulation was removed from analysis. Qualitatively out of focus images were also removed from the data quantification.
Replication	The Gal4-UAS system was used to generate perturbations within the wing imaginal disc in our studies. Multiple Gal4 drivers were used to carry out perturbations in different compartments of the wing imaginal disc. Data was analyzed to make sure that the observed traits were replicated irrespective of the choice of Gal4 driver used. All the data for replicates has been included within the manuscript as part of supplementary figures.
Randomization	Within our experimental design, the use of vials for growing Drosophila serves as a critical step for randomization. First flies are grown in multiple vials and data is collected from randomly selected and properly staged vial. Further when selecting a larvae for wing disc dissection, we randomly choose larvae from the staged vials without any predetermined criteria. Lastly each larvae contains a pair of wing discs. We chose one of the two without a left-right bias making the process of randomization robust.
Blinding	Blinding in our experiments was achieved by making sure that the experimentalists were unaware of the phenotypic outcomes during dissection and sample preparation. This helped in removing bias by removing selection of larvae based on known phenotypic outcomes.

Reporting for specific materials, systems and methods

We require information from authors about some types of materials, experimental systems and methods used in many studies. Here, indicate whether each material, system or method listed is relevant to your study. If you are not sure if a list item applies to your research, read the appropriate section before selecting a response.

Materials & experimental systems

n/a	Involved in the study
☐	☒ Antibodies
☒	☐ Eukaryotic cell lines
☒	☐ Palaeontology and archaeology
☐	☒ Animals and other organisms
☒	☐ Clinical data
☒	☐ Dual use research of concern
☒	☐ Plants

Methods

n/a	Involved in the study
☒	☐ ChIP-seq
☒	☐ Flow cytometry
☒	☐ MRI-based neuroimaging

Antibodies

Antibodies used

The following primary antibodies were used: Phospho-Smad1/5 (Ser463/465) (41D10) (1:300, Rabbit, Cell Signaling Technology #9516S), P-Histone H3 (1:500, Rabbit, Cell Signaling Technology #9701S), Phospho-Myosin Light Chain 2 (Ser19) (1:50, Rabbit, Cell Signaling Technology #3671S), α-Rho1 (1:10, Mouse, Developmental Studies Hybridoma Bank p1D9), Integrin betaPS (myospheroid) (1:5, Mouse, Developmental Studies Hybridoma Bank CF.6G11), α-Collagen IV antibody (1:5, Rabbit, Abcam ab6586).

The following dyes and secondary antibodies were α-Mouse Alexa Fluor™ 568 (1:500, Goat, Thermo Fisher Scientific A-11031), α-Rabbit Alexa Fluor™ 647(1:500, Goat, Thermo Fisher Scientific A32733), DAPI (1:500, Sigma Aldrich D9542), Fluorescein Phalloidin (1:500, Thermo Fisher Scientific F432).

Validation

All the acquired antibodies are commercially acquired and previously validated and used in specific to studies related to Drosophila wing imaginal disc.
1. Phospho-Smad1/5 (Ser463/465) Refer supplementary figure 17 for validation of the antibody.
2. P-Histone H3: https://www.cellsignal.com/products/primary-antibodies/phospho-histone-h3-ser10-antibody/9701
3: Phospho-Myosin Light Chain 2 (Ser19): Cell Signaling Technology's Phospho-Myosin Light Chain 2 (Ser19) Antibody is a Rabbit Polyclonal antibody. This antibody has been shown to work in applications such as: Immunocytochemistry, Immunofluorescence, and Western Blot. The Phospho-Myosin Light Chain 2 (Ser19) Antibody was generated using MYL9, and myosin light chain 9 as the antigen and it reacts with Homo Sapiens, Human, Mouse, Rat, and Drosophila/Arthropod. (Source:)https://www.biocompare.com/9776-Antibodies/118126-PhosphoMyosin-Light-Chain-2-Ser19-Antibody/#citations
4. α-Rho1: (Refer reactivity in the attached link) https://dshb.biology.uiowa.edu/p1D9-anti-rho1
5. Integrin betaPS: (Refer reactivity in the attached link) https://dshb.biology.uiowa.edu/CF-6G11
6. Collagen IV antibody: (Refer the online database) https://www.biocompare.com/Product-Reviews/331834-A-Collagen-IV-antibody-that-works-for-Drosophila/

Animals and other research organisms

Policy information about studies involving animals; ARRIVE guidelines recommended for reporting animal research, and Sex and Gender in Research

Laboratory animals

Drosophila melanogater was used as the model organism for this study. The wild-type Oregon-R fly line is a long-standing stock in the Zartman lab acquired from the Niryakobi lab. Several transgenic lines were commercially acquired from Bloomington Drosophila Stock Center (BDSC). The following transgenic stocks were obtained: UAS-RyRRNAi (BDSC#31540), UAS-InsRDN(BDSC#8253), UAS-InsRCA(BDSC#8263), UAS-TkvRNAi (BDSC#31041), UAS-TkvCA(BDSC#36537), Nubbin-Gal4 (BDSC#25754), Engrailed-Gal4 (BDSC#25752), UAS-Myc (BDSC#9674), UAS-mTorRNAi (BDSC#33951), UAS-MycRNAi (BDSC#25783), and UAS-mTorDN (BDSC#7013). We requested and received the ani:RBD reporter fly line (developed by Dr. Thomas Lecuit's lab) from Dr. Lynn Cooley. Wing imaginal discs, the model organ for this study, was dissected from staged larvae. The larval age has been indicated for all the experiments presented within manuscript.

Wild animals

No wild animals were used in te study.

Reporting on sex

N/A

Field-collected samples

No field collected samples were used in the study.

Ethics oversight

No ethical approval or guidance was required.

Note that full information on the approval of the study protocol must also be provided in the manuscript.

Plants

Seed stocks	N/A
Novel plant genotypes	N/A
Authentication	N/A
